# Predictive validity of admission tests and educational attainment on preclinical academic performance – a multisite study

**DOI:** 10.1186/s12909-025-07974-2

**Published:** 2025-09-23

**Authors:** Malvin Jaehn, Johanna Hissbach, Madita Frickhoeffer, Daniel Weppert, Alexander Zimmerhofer, Wolfgang Hampe, Martina Kadmon, Nicolas Becker

**Affiliations:** 1 Department of Psychology, Heidelberg University, Heidelberg, Germany; 2 Medical Faculty, Heidelberg University, Heidelberg, Germany; 3 Department of Biochemistry and Molecular Cell Biology, University Medical Center Hamburg Eppendorf (UKE), Hamburg, Germany; 4 Institute for Test Development and Talent Research, ITB Consulting GmbH, Bonn, Germany; 5 Faculty of Medicine, University of Augsburg, Augsburg, Germany; 6 Department of Individual Differences and Psychodiagnostics, Saarland University, Saarbrucken, Germany

**Keywords:** Medical school selection, Cognitive ability, Educational attainment, Admission testing, Predictive validity, Range restriction, Undergraduate entry

## Abstract

**Background:**

Educational attainment and admission tests have a longstanding history in the selection of medical students and are often used simultaneously in selection processes. Their value in the admission process is most frequently assessed by their ability to predict academic performance in medical school. However, their simultaneous use may overlook an overlap in their predictive validity. The present study aims to assess the predictive validity of both educational attainment and admission tests, as well as their incremental validities. In addition, subtest analyses are conducted to gain a more profound understanding of admission tests’ predictive power.

**Methods:**

A survey amongst test-takers of the German admission tests was conducted in 2022 and 2023. Self-reported preclinical performance was matched with admission test scores (i.e., TMS and HAM-Nat). Educational attainment was assessed by high-school grade point average (GPA). Based on *n* = 2113 medical students, hierarchical multiple regression analyses were conducted. Pearson’s correlations were used to assess the relationship of subtests with academic performance. For all analyses, the effects of range restriction were diminished using a multivariate correction formula.

**Results:**

TMS and HAM-Nat as well as high-school GPA predicted academic performance separately. However, while both admission tests demonstrate substantial incremental validity over high-school GPA, the reverse is true to a far lesser extent. High-school GPA exhibits only small predictive power whilst controlling for admission test scores. Subtests containing elements of both crystallized and fluid intelligence proved to be of moderate effect size.

**Conclusions:**

The findings of this study suggest that both admission tests and high-school GPA are well-suited as selection criteria in the admission process. Given the growing concerns regarding high-school GPA, admission tests emerge as a compelling alternative, particularly because of their stronger predictive power. Within each examined admission test, content-rich subtests containing elements of both crystallized and fluid intelligence demonstrated the strongest association with academic performance in preclinical years, in line with the test-criterion content match hypothesis.

**Supplementary Information:**

The online version contains supplementary material available at 10.1186/s12909-025-07974-2.

## Introduction

Selection into medical school has primarily been based on previous educational attainment, typically evaluated during an initial screening process [1]. This practice is driven by the notion that high-school grade point average (GPA), as a measure of educational attainment, serves as a reliable individual predictor of academic performance [2] and the convenience of obtaining applicants’ high-school GPA. Research consistently supports the predictive validity of high-school GPA in selecting medical students [3, 4]. However, there is significant variability in the extent to which high-school GPA predicts outcomes. While on a construct level, for example, Mc Manus et al. [5] report high validities of GPA of about 65% of true variance in first-year medical school performance, other findings suggest a lesser degree of prediction of academic performance with about 23% of explained variance in undergraduate medical training performance and 6% in postgraduate competency [3]. For many years, however, there has been an ongoing debate about the use of high-school GPA as a selection criterion, which centers on issues of grade inflation [6–8], doubts regarding its comparability between federal states and school types, as well as its consistency between different teachers (e.g [9, 10]). Critics also argue that high-school GPA lacks specificity, especially regarding the requirements of individual study courses like medicine or specific universities [11].

Alongside high-school GPA, admission tests have a long-standing history as a selection tool. A review of the most recent findings on the predictive validity of admission tests reveals inconclusive evidence, amongst other factors mostly due to different tests being looked at [1]. In their most recent meta-analysis on the previous version of the Medical College Admission Test (MCAT) based on 23 studies, Donnon et al. [12] infer that the MCAT only exhibits small to medium values for predictive validity for academic performance in both preclinical and clinical years. This is in line with the review on the University Clinical Aptitude Test (UCAT) by Bala et al. [13], who conclude that cognitive and verbal reasoning tests only weakly predict academic performance in medical school. Recent findings from Busche et al. [14] and Hanson et al. [15], however, show strong predictive capabilities of the revised MCAT. Similarly, a review by Greatrix et al. [16] suggests that the UCAT maintains strong predictive power throughout medical training, with indications of the predictive power even increasing with time, which may be explained by a sustained cognitive performance impact. Another reason for the heterogeneous findings might be that the type of correction used and whether any correction was applied at all for the issue of range restriction vary considerably between studies and add to the difficulty of comparing research results, as the omission of any correction results in underestimation of predictive validity.

The coexistence of high-school GPA and admission tests as selection tools is also evident in the selection processes of medical faculties in Germany. A judgment of the Constitutional Court [17] led to a fundamental reform of the selection process. Since 2020, all German medical faculties, which only admit students through undergraduate entry, must consider at least one additional selection measure with significant weight in addition to high-school GPA (cf. to the Supplementary Material 1 Information 1 for a further description of the selection processes in Germany). In practice, admission tests turn out to be the measure of choice. Following the court ruling, a state-funded student admission research consortium (“Studierendenauswahlverbund” (*stav)*) was established. As part of the stav’s research program, a thorough examination on the existing cognitive admission tests was conducted. German medical schools use either the *Test for Medical Studies* (TMS) or the *Hamburg Assessment for Medical Studies*,* Natural Sciences* (HAM-Nat) in their admission process. Both tests are subject-specific tests assessing applicants’ aptitude, though with different emphases. The TMS was developed as a focused assessment of academic aptitude, integrating elements from conventional intelligence test batteries into a context relevant to medical training. In contrast, the HAM-Nat primarily encompasses a knowledge section covering natural sciences for medical studies and tests related to numerical, verbal, and figural reasoning. A comprehensive overview of prior research on the predictive validity of both tests is given in the meta-analysis by Schult et al. [18], revealing a validity coefficient of *ρ* = 0.47 based on 12 studies for a pooled analysis of the TMS and HAM-Nat. However, only single-site studies have so far been conducted on both tests [19–22], which do not consider the variety of medical schools in Germany and, thus, limit the generalizability of previous results [23]. Furthermore, the aforementioned studies may be outdated due to changes in study conditions, specifically the change in selection processes and the introduction of reform curricula. The shift towards higher high-school GPA scores over the past years in Germany (according to the Federal Agency for Civic Education, 17 out of 1000 high-school graduates received a perfect grade of 1.0 in 2017 – marking an increase of nearly 70% compared to 2007) further call for a reevaluation of both admission tools regarding their predictive validity. Therefore, this multisite study with data from all German medical faculties, aims to answer the following research questions:

1. Do TMS and HAM-Nat total scores predict academic performance in preclinical years?
2. Do TMS and HAM-Nat total scores predict academic performance in preclinical years over high-school GPA and vice versa?
3. Does the relationship with academic performance in preclinical years differ between specific subtests within TMS and HAM-Nat?

On a broader scale, this study provides pertinent evidence in light of the upcoming consolidation of the TMS and the HAM-Nat to a nationwide admission test in Germany.

## Methods

### Procedure and participants

An online survey (cf. to Supplementary Material 2) asking participants about their academic performance in medical training was conducted in two waves in May 2022 and May 2023. Participants were former test-takers of the TMS and HAM-Nat who had consented to being contacted for research purposes (*N* = 10727). A total of *n* = 5464 participants completed the survey amounting to a response rate of 50.9%. Among these, *n* = 2113 were deemed valid cases representing individuals who reported enrollment in medicine and met inclusion criteria (i.e., report of predictor variables and at least one measure of academic performance). Exclusion was defined by non-enrollment of applicants (*n* = 2336), non-relevant admission quotas to this study (*n* = 107), for example, admission via lawsuit, and missing data on admission tests (*n* = 306), high-school GPA (*n* = 27), outcome variables (*n* = 566) and age (*n* = 9). Data of all former test-takers was used to correct for effects of range restriction. Admission test scores were provided directly from the test organizers. All other data were gathered through self-reports. Overall, data from six cohorts (year of test participation: 2017–2022) from all 38 medical schools in Germany were included, with 36 schools utilizing the TMS and two utilizing the HAM-Nat. The cohorts of 2020–2022 represented the predominant proportion with 26.3%, 40.3%, and 27.9% of participants, respectively.

### Predictor variables

Predictor variables were high-school GPA and admission test scores (either TMS or HAM-Nat scores). Demographic variables (age and gender) were included in the analyses to control for potential effects on high-school GPA, admission test scores, and outcome variables (e.g., [24]). High-school GPA is derived from the German matriculation examination (*Abitur)* held at the end of secondary school and is comparable to A-levels or exit examinations in other countries. They range from 1.0 to 4.0, with 1.0 being the best grade.

#### Admission tests

The TMS is designed to measure different cognitive abilities relevant to the medical field and is comprised of eight subtests. The overall test demonstrates an excellent internal consistency (Cronbach’s Alpha ranging between 0.91 − 0.92; cf. to Supplementary Material 1 Table 1) and has shown to predict preclinical academic performance in single-site studies [22, 25]. It is based on the classical test theory. To calculate the total test score as well as individual subtest scores, the number of correctly solved items (of the overall test or a subtest, respectively) is first added up to a raw score. Raw scores are then standardized with a mean of 100 and a standard deviation of 10. The HAM-Nat consisted of three subtests during the time of the study. The core of the HAM-Nat is a knowledge test related to pertinent natural science, which has shown high internal consistency as well (Cronbach’s Alpha ranging between 0.88 − 0.91; cf. to Supplementary Material 1 Table 1) and predictive validity [20, 26]. The other two subtests are a verbal task and a numerical reasoning task. Test scores are expressed in theta values (with a mean of 0 and a standard deviation of 1) as the HAM-Nat is based on the item response theory framework. The HAM-Nat total score is the sum of the knowledge test being weighted 75% and both reasoning tests being weighted 12.5%. Both tests (i.e., TMS and HAM-Nat) exclusively consist of items in a single-response multiple-choice format. Descriptions of both TMS and HAM-Nat subtests are presented in Table 1. Further details are given in the Supplementary Material 1 Information 2 and 3. In regard to the testing procedures, both tests are similar as they are on-site, proctored paper-pencil tests and utilize item banks from which items are assembled for each test version. While the TMS consists exclusively of unreleased items unknown to test-takers, approximately 55% of the HAM-Nat´s knowledge test items have been previously published, prompting test-takers to engage in targeted preparation.

**Table 1:** Tasks, number of items and duration of the subtests of HAM-Nat and TMS

Test	Subtest (abbreviation)	Task (short description)	Number of Items	Duration in Min
HAM-Nat	KT	medicine-specific knowledge test	60	90
	VRT	verbal reasoning task	16	15
	NRT	numerical reasoning task	16	15
TMS	BMS	comprehension of basic medical or natural scientific contents presented in short texts	24	60
	QFP	solving short quantitative and formal problems	24	60
	TC	analysis and comprehension of longer textbook-like texts	24	60
	DT	analysis and interpretation of diagrams and tables within a medical and scientific context	24	60
	VST	visual search task	24	30
	MRT	mental rotation task of three-dimensional objects	24	15
	FMT	memory task of figural information	20	9
	VMT	memory task of verbal information	20	13

A more detailed description of the tasks including sample items can be found in the Supplementary Material 1 Information 2 and 3

#### Outcome variables

The study design defined two outcome variables to measure academic performance: (i) the grade point average over the assessments during the first two preclinical years of undergraduate training (PCGPA), a commonly used outcome measure in studies of predictive validity in student selection and (ii) the result of the first part of the medical licensing examination (M1), which is taken at the end of the second study year. Participants reported their PCGPA based on their progress in the study program until the survey. The M1 is a standardized examination throughout Germany and comprehensively tests the contents of the pre-clinical study program (i.e., physics, physiology, chemistry, biochemistry, biology, anatomy, and medical psychology/sociology). A few universities in Germany offer a different study program that integrates pre-clinical and clinical teaching. Students from these universities did not participate in the M1 but received a substitutional grade on the basis of examinations provided by the individual medical school instead. For reasons of simplicity, the substitutional grade was treated as M1. The final grade of the M1 is the average of an equally weighted written part in a multiple-choice format and an oral part. The written part shows an excellent reliability with Cronbach´s Alpha ranging from 0.91 to 0.96 [27]. Both the results of PCGPA and M1 range from 1.0 to 4.0, with 1.0 being the best grade.

### Correction for range restriction

Due to the selection of test-takers via high-school GPA *and* admission test scores, both direct and indirect effects of range restriction in the predictor variables are inherently present [28], reducing predictor-outcome correlations. To diminish this distortion, correction for range restriction is recommended by many scholars (e.g [29]), and, for example, included in the *Standards for educational and psychological testing* [30]. As the selection scenario of this study includes multiple variables in the selection process, performing multivariate correction [31, 32] is most adequate as multivariate correction has been shown to outperform univariate correction by a substantial amount [33]. More concretely, we applied the multivariate correction by Lawley [32], which is based on variance-covariance matrices. Following the theorem, a variance-covariance matrix of an unrestricted sample (i.e., medical school applicants) is used to estimate the unknown unrestricted variances and covariances of a restricted sample (i.e., applicants who reported enrollment and provided academic performance data). The corrected variance-covariance matrix of the restricted sample can then be used for further analyses (i.e., regression analyses that account for range restriction). A more technical description of the correction formula is given, for example, by Held and Foley [33] and Ree et al. [34].

### Data analyses

To examine the predictive and incremental validity of TMS and HAM-Nat over high-school GPA and vice versa, we conducted hierarchical multiple regression analyses. As the vast majority of test-takers solely participated in one of the admission tests, analyses were conducted in separate samples for TMS and HAM-Nat (hereafter referred to as the TMS and HAM-Nat sample, respectively). In a first step, we established a baseline model consisting of the control variables age and gender (model 1). Next, we added either high-school GPA (model 2a) or the respective admission test (model 2b) to the baseline model. In model 3, both admission variables are added simultaneously. The series of regression analyses were conducted at the nationwide level across all universities, and separately, by categorizing universities based on the admission test utilized in their selection process (i.e., universities with selection via TMS or HAM-Nat, respectively). For all analyses, gender-diverse participants were excluded due to an insufficient sample size. To determine whether the relationship with academic performance differs between subtests, we calculated Pearson´s correlation coefficients (*r*). Results of regression analyses were interpreted based on *R*^*2*^, the incremental *R*^*2*^ (*∆R*^*2*^), and predictors’ standardized coefficients (β). All values are presented after the correction of range restriction using the multivariate correction formula by Lawley [32]. For transparency, the uncorrected values are presented in parentheses. Results of the analyses without adjustment for age and gender (e.g., for review and meta-analytic purposes) are shared in the Supplementary Material 1 Table 3. Statistical analyses were carried out with the statistics software R (v4.2.3 [35]). The R code is shared in the Supplementary Material 3.

## Results

Descriptive statistics of both TMS and HAM-Nat test-takers and incumbents (i.e., test-takers who reported enrollment in medicine) are reported in Table 2. The ratio of test-takers to incumbents reflects the ratio in the respective total population well [36], amounting to 27.3% in case of the TMS and 17% in case of the HAM-Nat. Accordingly, the imperative effect of admission on sample characteristics can be found in the underlying data. Both TMS and HAM-Nat incumbents showed a significantly higher admission test score (*d*_*TMS*_ = 0.78 and *d*_*HAM−Nat*_ = 0.68) and numerically lower high-school GPA (*d*_*TMS*_ = 0.55 and *d*_*HAM−Nat*_ = 0.46) than test-takers who did not report enrollment. Demographic variables did not differ meaningfully between incumbents and test-takers showing negligible effect sizes for age (*d*_*TMS*_ = − 0.18 and *d*_*HAM−Nat*_ = − 0.04) and gender (*V*_*TMS*_ = 0.04 and *V*_*HAM−Nat*_ = 0.04). The degree of range restriction of each predictor variable is shown in the Supplementary Material 1 Table 2. For age and gender, it ranges between 0.98 and 1.06, whereas for high-school GPA and the admission tests, it ranges from 0.85 to 1.06.

**Table 2:** Descriptive statistics of demographic, admission, and outcome variables of TMS and HAM-Nat test-takers and incumbents

	TMS		HAM-Nat	
	Test-takers (n = 8796)	Incumbents (n = 1880)	Test-takers (n = 5020)	Incumbents (n = 706)
Variable	*n (%)*	*n (%)*	*n (%)*	*n (%)*
Gender				
female	6447 (73.29)	1310 (69.68)	3486 (69.44)	455 (64.45)
male	2327 (26.46)	563 (29.95)	1525 (30.38)	251 (35.55)
gender-diverse	22 (< 0.01)	7 (< 0.01)	9 (< 0.01)	
Variable	*M (SD)*	*M (SD)*	*M (SD)*	*M (SD)*
Age	20.77 (2.37)	20.48 (2.52)	21.31 (2.64)	21.21 (2.57)
High-school GPA	1.70 (0.47)	1.52 (0.45)	1.80 (0.48)	1.63 (0.43)
TMS	102.47 (9.43)	107.43 (7.97)		
HAM-Nat			0.28 (0.87)	0.77 (0.92)
PCGPA^a^		2.17 (0.64)		2.14 (0.64)
M1^b^		2.39 (0.81)		2.28 (0.84)

*n* = sample size; *M* = mean; *SD* = standard deviation

^a^PCGPA was available for *n*_*TMS*_ = 1867 and *n*_*HAM−Nat*_ = 699

^b^M1 grades were available for *n*_*TMS*_ = 377 and *n*_*HAM−Nat*_ = 233

Correlations among demographics, admission criteria, and outcome variables are shown in Table 3. For both outcome variables, correlations with admission test scores were higher than with high-school GPA. Outcome variables PCGPA and M1 correlated strongly in both the TMS and HAM-Nat incumbents (*r*_*TMS*_ = 0.64 and *r*_*HAM−Nat*_ = 0.69). Notably, the effect of using multiple compensating selection criteria (e.g., as described by Zimmermann et al. [37]) can be observed in the underlying data: In the population of both TMS and HAM-Nat *incumbents*, high-school GPA and admission test scores do not show a significant correlation (*r*_*TMS*_ = − 0.10 and *r*_*HAM−Nat*_ − 0.08). In the population of *test-takers*, however, a significant correlation was found (*r*_*TMS*_ = − 0.28 and *r*_*HAM−Nat*_ − 0.28), which differed significantly from the correlation within the sample of incumbents (*z*_*TMS*_ = 7.37; *p* <.001 and *z*_*HAM−Nat*_ = 5.15; *p* <.001).

**Table 3:** Pearson correlations of demographic variables, admission criteria, and outcome variables of TMS and HAM-Nat incumbents

Test	Variable	Gender	Age	High-school GPA	Admission test	PCGPA
TMS^a^	Age	0.05** (0.06**)	-			
	High-school GPA	0.03** (0.10**)	0.46** (0.59**)	-		
	Admission test	0.09** (0.06*)	− 0.17** (−0.16**)	− 0.28** (−0.10**)	-	
	PCGPA^b^	− 0.10** (−0.09**)	0.11** (0.12**)	0.19** (0.14**)	− 0.23** (−0.18**)	-
	M1^c^	− 0.15** (−0.12*)	0.17** (0.18**)	0.26** (0.21**)	− 0.26** (−0.18**)	(0.64**)^d^
HAM-Nat^a^	Age	0.02 (0.04)	-			
	High-school GPA	0.03 (0.09*)	0.41** (0.55**)	-		
	Admission test	0.20** (0.20**)	− 0.05** (−0.06)	− 0.28** (−0.08*)	-	
	PCGPA^b^	− 0.04 (−0.03)	0.05 (0.07)	0.22** (0.15**)	− 0.36** (−0.35**)	-
	M1^c^	− 0.11 (−0.10)	0.19** (0.24**)	0.35** (0.28**)	− 0.48** (−0.46**)	(0.69**)^d^

Values in parentheses are not corrected for range restriction

^a^*N*_*TMS*_ = 1873 and *N*_*HAM−Nat*_ = 706

^b^PCGPA was available for *n*_*TMS*_ = 1860 and *n*_*HAM−Nat*_ = 699

^c^M1 grades were available for *n*_*TMS*_ = 375 and *n*_*HAM−Nat*_ = 233

^d^Reporting the corrected correlation of the outcome variables PCGPA and M1 was not feasible. PCGPA and M1 were available for for *n*_*TMS*_ = 362 and *n*_*HAM−Nat*_ = 226

*indicates *p* <.05. **indicates *p* <.01

### Regression analyses

Results of hierarchical regression analyses testing the associations of high-school GPA and admission test scores with academic performance across all medical schools in Germany while controlling for gender and age are depicted in Table 4. In the TMS sample, the baseline model with age and gender (model 1) explained 2.3% of the variance in PCGPA and 5.4% of the variance in M1. In the HAM-Nat sample, the proportion of variance explained amounted to 0.4% and 5.0%, respectively. Adding high-school GPA to the baseline models (cf. models 2a) resulted in a significant improvement with between 2.4% and 9.3% of additional variance explained. Similarly, adding admission test scores to the baseline models (cf. models 2b) resulted in another increment with between 4.1% and 21.2% of variance explained. Adding both high-school GPA *and* admission test scores to the prediction of academic performance (cf. models 3) resulted in a significant improvement from models 2a with between 2.9% and 15.0% of additional variance explained. Overall, independent variables predicted M1 better than PCGPA in both the TMS sample (*R*^*2*^_*PCGPA*_ = 0.08 and *R*^*2*^_*M1*_ = 0.13) and the HAM-Nat sample (*R*^*2*^_*PCGPA*_ = 0.14 and *R*^*2*^_*M1*_ = 0.29).

**Table 4:** Results of hierarchical regression analyses of TMS and HAM-Nat incumbents

Test	Model	Predictor	PCGPA			M1		
			β	*R* ^2^	∆*R*^2^	β	*R* ^2^	∆*R*^2^
TMS^a^	1	Gender	− 0.106** (−0.097**)			− 0.159** (−0.129*)		
		Age	0.114** (0.123**)	0.023 (0.023)	-	0.178** (0.181**)	0.054 (0.048)	-
	2a	Gender	− 0.108** (−0.106**)			− 0.161** (−0.144**)		
		Age	0.034 (0.048)			0.068 (0.067)		
		GPA	0.173** (0.127**)	0.047 (0.033)	0.024** (0.010**)	0.239** (0.181**)	0.099 (0.067)	0.045** (0.019**)
	2b	Gender	− 0.087** (−0.087**)			− 0.138** (−0.137**)		
		Age	0.077** (0.097**)			0.138** (0.164**)		
		TMS	− 0.206** (−0.155**)	0.064 (0.046)	0.041** (0.023**)	− 0.225** (−0.172**)	0.103 (0.077)	0.049** (0.029**)
	3	Gender	− 0.090** (−0.095**)			− 0.143** (−0.151**)		
		Age	0.023 (0.024)			0.056 (0.059)		
		GPA	0.128** (0.125**)		0.012** (0.010**)^c^	0.192** (0.167*)		0.027** (0.016*)^c^
		TMS	− 0.180** (−0.154**)	0.076 (0.056)	0.029** (0.023**)^d^	− 0.186** (−0.164**)	0.130 (0.093)	0.031** (0.026**) ^d^
HAM-Nat^b^	1	Gender	− 0.036 (−0.033)			− 0.114 (−0.120)		
		Age	0.052 (0.072)	0.004 (0.006)	-	0.195** (0.248**)	0.050 (0.070)	-
	2a	Gender	− 0.041 (−0.044)			− 0.120 (−0.132*)		
		Age	− 0.047 (−0.014)			0.059 (0.117)		
		GPA	0.242** (0.158**)	0.053 (0.023)	0.049** (0.017**)	0.333** (0.230**)	0.143 (0.106)	0.093** (0.036**)
	2b	Gender	0.036 (0.038)			− 0.019 (−0.023)		
		Age	0.032 (0.046)			0.169** (0.197**)		
		HAM-Nat	− 0.361** (−0.351**)	0.129 (0.124)	0.125** (0.118**)	− 0.471** (−0.439**)	0.262 (0.253)	0.212** (0.183**)
	3	Gender	0.026 (0.027)			− 0.034 (−0.036)		
		Age	− 0.022 (−0.022)			0.091 (0.092)		
		GPA	0.139** (0.127**)		0.014** (0.011**)^c^	0.201** (0.186**)		0.031** (0.023**)^c^
		HAM-Nat	− 0.323** (−0.343**)	0.143 (0.135)	0.090** (0.112**) ^d^	− 0.415** (−0.426**)	0.293 (0.276)	0.150** (0.170**) ^d^

Values in parentheses are not corrected for range restriction

*∆R*^*2*^ = change in *R*^2^. β = standardized regression coefficient

^a^*N*_*PCGPA*_ = 1860 and *N*_*M1*_ = 375

^b^*N*_*PCGPA*_ = 699 and *N*_*M1*_ = 233

^c, d^*∆R*^*2*^ of model 3 is calculated over model 2a (^d^) and model 2b (^c^)

*indicates *p* <.05. ** indicates *p* <.01

Given Germany´s selection situation, where universities either employ the TMS *or* HAM-NAT for admission, additional regression analyses were conducted, dividing universities by the admission test used in their selection process. Results are presented in Table 5. For the TMS, the pattern of significance and variance explained was similar across universities, regardless of whether the TMS was used for selection or not. However, the absolute values of variance explained were considerably higher for universities where the TMS was not utilized for selection (*R*^*2*^_*PCGPA*_ = 0.281 and *R*^*2*^_*M1*_ = 0.371). For the HAM-Nat, results were similar across all universities, regardless of whether the HAM-Nat was used for selection or not. However, the amount of variance explained in M1 by adding high-school GPA to the baseline model (2a) was considerably higher for universities that used the HAM-Nat for selection (*∆R*^*2*^ = 0.129) compared to those that did not (*∆R*^*2*^ = 0.023).

**Table 5:** Results of hierarchical regression analyses divided by universities with selection via TMS and HAM-Nat

Test	Outcome	Model	Universities with selection via TMS			Universities with selection via HAM-Nat		
			*n*	*R* ^2^	∆*R*^2^	*n*	*R* ^2^	∆*R*^2^
TMS	PCGPA	1	1673	0.024 (0.025)		**187**	**0.050 (0.034)**	
		2a		0.047 (0.035)	0.023** (0.010**)		**0.130 (0.066)**	0.080** (0.032**)
		2b		0.065 (0.047)	0.041** (0.022**)		**0.245 (0.174)**	0.195** (0.140**)
		3		0.076 (0.057)	0.029** (0.022**) ^c^		**0.281 (0.195)**	0.151** (0.129**) ^c^
	M1	1	285	0.052 (0.050)		**90**	**0.039 (0.017)**	
		2a		0.079 (0.059)	0.027** (0.009)		**0.264 (0.127)**	0.225** (0.110**)
		2b		0.112 (0.088)	0.060** (0.038**)		**0.219 (0.130)**	0.180** (0.113**)
		3		0.124 (0.096)	0.045** (0.037**) ^c^		**0.371 (0.217)**	0.107** (0.090**) ^c^
HAM-Nat	PCGPA	1	**393**	**0.004 (0.001)**		306	0.019 (0.027)	
		2a		**0.034 (0.011)**	0.030** (0.010*)		0.115 (0.045)	0.096** (0.018*)
		2b		**0.106 (0.088)**	0.102** (0.087**)		0.224 (0.132)	0.205** (0.105**)
		3		**0.113 (0.094)**	0.079** (0.083**) ^c^		0.257 (0.159)	0.142** (0.114**) ^c^
	M1	1	**110**	**0.024 (0.035)**		123	0.082 (0.117)	
		2a		**0.047 (0.035)**	**0.023 (< 0.001)**		0.211 (0.181)	0.129** (0.064**)
		2b		**0.230 (0.208)**	0.206** (0.173**)		0.202 (0.173)	0.120** (0.056**)
		3		**0.231 (0.209)**	0.184** (0.174**) ^c^		0.274 (0.231)	0.063** (0.050**) ^c^

Results for universities where the respective test was not used for selection are highlighted in bold

*∆R*^*2*^ = change in *R*^2^

^c^*∆R*^*2*^ of model 3 is calculated over model 2a

*indicates *p* <.05. ** indicates *p* <.01

### Subtest analyses

Associations of TMS and HAM-Nat subtests with academic performance are presented in Table 6. Overall, associations with both PCGPA and M1 proved to be small to moderate, although minor differences were noticeable. For the TMS, subtests consisting of reasoning tasks and text comprehension showed a stronger association (−0.17 ≤ *r* ≤ −0.35) than memory tasks, the visual search task, and the pattern recognition task (−0.04 ≤ *r* ≤ −0.15). For the HAM-Nat, the knowledge test showed the highest association (−0.35 ≤ *r* ≤ −0.47), whereas associations of the HAM-Nat´s reasoning tests were considerably lower (−0.11 ≤ *r* ≤ −0.25).

**Table 6:** Pearson correlations of TMS and HAM-Nat subtests with outcome variables

Test	Subtest	PCGPA	M1
TMS^a^	BMS	− 0.21** (−0.17**)	− 0.35** (−0.30**)
	QFP	− 0.25** (−0.21**)	− 0.27** (−0.20**)
	DT	− 0.18** (−0.14**)	− 0.30** (−0.24**)
	TC	− 0.17** (−0.12**)	− 0.29** (−0.22**)
	MRT	− 0.13** (−0.09**)	− 0.15** (−0.08)
	FMT	− 0.05 (−0.01)	− 0.05 (0.02)
	VMT	− 0.11** (−0.07**)	− 0.04 (0.03)
	VST	− 0.12** (−0.09**)	− 0.05 (−0.02)
HAM-Nat^b^	KT	− 0.35** (−0.34**)	− 0.47** (−0.46**)
	NRT	− 0.17** (−0.15**)	− 0.25** (−0.23**)
	VRT	− 0.11 (−0.09)	− 0.15** (−0.09)

Values in parentheses are not corrected for range restriction

^a^*N *_*PCGPA*_ = 1860 and *N*_*M1*_ = 375

^b^*N *_*PCGPA*_ = 699 and *N*_*M1 *_= 233

*indicates *p* < .05. ** indicates *p*< .01

## Discussion

Against the backdrop of Germany’s unique medical student selection situation involving two different admission tests as well as recent changes in the selection procedure, the present study represents the first nationwide multisite investigation into the predictive validity of admission tests and high-school GPA in Germany. Academic performance was assessed by the self-reported grade point average in preclinical years (PCGPA) and the self-reported results of a nationally standardized examination (M1). Our research is distinguished by a substantial and representative sample of test-takers from all German public medical schools. This provides us with a robust foundation for the correction formula employed, which is designed to accommodate direct and indirect range restriction within a multivariate framework and allows us to adequately compare high-school GPA and admission test scores regarding their predictive power.

In our study, we found significant associations of admission test scores with academic performance in preclinical years. The magnitude of correlation coefficients ranged between 0.23 and 0.48 and is thereby in line with results of the meta-analysis by Schult et al. [18]. While the observed correlations in this study are slightly lower, it should be noted that no correction was made for criterion unreliability in this study. High-school GPA showed similar associations with academic performance though of slightly less strength with a range of 0.19 to 0.35. Both admission tests (i.e., TMS and HAM-Nat) exhibit significant added predictive value beyond high-school GPA, which is similar to Niessen et al. [38] in the context of an undergraduate psychology program. Notably, the reverse is true to a far lesser extent. High-school GPA does not contribute substantially to the prediction when controlling for admission test results. That being said, we observe wide ranges of absolute values of explained variance, from 4.1 to 20.6% for admission test scores and from 2.3 to 9.6% for high-school GPA, which vary depending on the subsample and criterion. The pattern of fluctuations between these values suggests that the M1 grade can be predicted more accurately than PCGPA. A result, that is likely attributed to a higher standardization, uniformity, and increased objectivity of the M1. Moreover, both admission tests and high-school GPA demonstrate stronger performance at individual sites compared to their performance when multiple locations are aggregated. These varying study conditions may introduce noise into the data, diminishing the predictive information. However, the pattern of variance explained by the predictors remains unchanged in all of the study conditions. It is important to note that the results of this study demonstrate (incremental) predictive validity under the assumption that admission test scores and high-school GPA are combined using optimal regression weights, and that these results apply specifically to this cohort. In practice, medical schools apply different - and varying – weighting schemes, inevitably reducing the (incremental) validity. While the implications of alternative weighting schemes lie beyond the scope of this study, they offer a promising direction for future research.

Besides the direct comparison of the predictive power of high-school GPA and admission tests, another aim of this study was to gain a more profound understanding of an admission test´s predictive power by investigating its association with academic performance for each subtest individually. The results of the subtest analyses for both tests suggest a stronger correlation with academic performance for subtests covering a substantial amount of medical or scientific content and containing elements of both crystallized and fluid intelligence, such as the knowledge test *KT* (from the HAM-Nat), and the reasoning tests *BMS*,* QFP*,* TC*, and *DT* (from the TMS). Correlations were of small to medium effect sizes. This outcome is in accordance with previous findings on the validity of curriculum-sampling tests in medicine and in other domains [39, 40] and may be explained by the better match of predictor and criterion of subtests with a medical focus, which was concluded by Sackett et al. [41].

Lastly, the results of this study may guide further analyses on the optimal combination of subtests of the TMS and HAM-Nat in the context of the endeavor to merge both tests into a nationally standardized test. This is particularly intriguing given that the predictive validity of the TMS is diminished by the inclusion of certain subtests with little or no predictive value, while other subtests outperform the total test score. A direct comparison of the TMS and HAM-Nat by examining the predictive validity of one test over the other was not advisable in this study, however, as analyses with participants that have undergone both tests would be required. Sample sizes for these analyses were quite low and, more importantly, analyses would be susceptible to substantial bias. This is because applicants who obtained an insufficient score in the TMS, often proceed with their goal to study medicine by taking the HAM-Nat (and vice-versa). Therefore, the correlation between TMS and HAM-Nat is close to zero when in fact both tests should be moderately associated. A comparable case is observed regarding the correlation between admission test scores and high-school GPA, highlighting the importance of an appropriate correction.

### Limitations

One limitation of this study is the self-reported nature of data, introducing potential biases, like, for example, motivated distortion, which describes the motivation of participants to purposely provide an inaccurate report of data (e.g., [42]). The meta-analysis of Kuncel et al. [43] on the validity of self-reported grades, however, indicated that the accuracy of self-reported data is satisfactorily met – particularly in the case of participants with a high GPA and high cognitive ability scores, which is generally the case in studies on medical students. Still, we advise readers to interpret the results of this study with this limitation in mind. The self-reported nature of data likely leads to an underestimation of correlations and regression coefficients which affects high-school GPA but not admission test scores and, therefore, limits the comparability of predictors. Another limiting factor of the present study is the uneven distribution of medical schools within the sample, as well as the fact that the HAM-Nat is only used by two medical schools for selection. Predictive validities of the TMS were considerably higher at medical schools where the HAM-Nat was used for selection. A possible explanation is the effect of range restriction, which was at least partially corrected for by the multivariate correction used, but was further reinforced by the sample distribution. Incumbents of universities, where the HAM-Nat was used for selection, were overrepresented in our final sample compared to the total population of medical students. Consequently, a higher percentage of the cohort and possibly a more representative sample from these universities was included in our study likely contributing to the increase in prediction compared to the more restricted cohorts from other universities. Due to the sample sizes per medical school, conducting hierarchical linear models to account for non-independence of students within medical schools was not feasible but should be considered in future research on these admission tests. Further exploration of this finding, including factors like location dependency, randomness, or other potential causes, fell outside the scope of this study.

## Conclusions

In conclusion, both, admission tests and high-school GPA predicted academic performance in preclinical years separately. Within each examined admission test, content-rich subtests containing elements of both crystallized and fluid intelligence demonstrated the strongest association with academic performance in preclinical years, in line with the test-criterion content match hypothesis by Sackett et al. [41].

## Supplementary Information

Supplementary Material 1.

Supplementary Material 2.

Supplementary Material 3.

## Data Availability

The datasets generated and analyzed during the current study are not publicly available due to privacy restrictions of the student admission research consortium (“Studierendenauswahlverbund” (*stav*)). Requests to access these datasets should be directed to kontakt@projekt-stav.de.

## Abbreviations

TMS: Test for Medical Studies
HAM-Nat: Hamburg Assessment for Medical Studies, Natural Sciences
GPA: grade point average
MCAT: Medical College Admission Test
UCAT: University Clinical Aptitude Test
stav: student admission research consortium (in German: Studierendenauswahlverbund)
PCGPA: grade point average over the assessments during the first two preclinical years of undergraduate training
M1: first part of the medical licensing examination
BMS: Basic understanding of medicine and the sciences
TC: Text comprehension
DT: Diagrams and tables
QFP: Quantitative and formal problems
MRT: Mental rotation task
FMT: Figural memory task
VMT: Verbal memory task
VST: Visual search task
KT: Knowledge test
VRT: Verbal reasoning test
NRT: Numerical reasoning test
