# Supplementary material for: Predictive validity of admission tests and educational attainment on preclinical academic performance – a multisite study
Source: BMC Med Educ. 2025 Sep 23;25:1255. doi: 10.1186/s12909-025-07974-2 (PMC12455761; doi:10.1186/s12909-025-07974-2)
Supplement: Supplementary file 1 — Supplementary Material 1. [file 12909_2025_7974_MOESM1_ESM.docx]

**Supplementary Material**

**Supplementary Information 1**

*Selection into medical schools in Germany.*

The selection process of medical schools in Germany is a quota system, that considers state regulations on the one hand and gives medical schools freedom in their selection on the other. In the case of each medical school, 30 percent of all admission slots are constrained for the GPA quota, in which solely the GPA is used to rank and admit applicants. Besides the GPA quota, two other quotas exist divided into a 60 percent quota (Auswahlverfahren der Hochschule, AdH; en. selection quota of the medical school) and a 10 percent quota (Zusätzliche Eignungsquote, ZEQ; en. additional eligibility quota). Each medical school decides which selection criteria are used in both of these quotas and how they are weighted, although certain constraints apply. In the 10 percent quota, selection criteria measuring aptitude to study medicine are generally allowed but considering GPA is explicitly prohibited. In the 60 percent quota, medical schools are again free in their choice of selection criteria but are obligated to use two selection criteria besides GPA, one of them being an admission test.

**Supplementary Information 2**

*Description of tasks in the subtests of TMS.*

*Basic understanding of medicine and the sciences* (BMS) assesses the comprehension of medical or natural scientific contents presented in short texts and the ability to think deductively. This reasoning task focuses on verbal thinking and nuanced language comprehension. It requires participants to understand and integrate information, establish causal relationships, and construct mental representations.

*Text comprehension* (TC) assesses the analysis and comprehension of longer textbook-like texts. This reasoning task requires nuanced language comprehension and the application of external text-processing techniques such as note-taking and highlighting, as well as the ability to organize information and draw conclusions.

*Diagrams and tables* (DT) assesses the ability to analyze and interpret graphs and tables within a medical and scientific context. Participants are required to understand relationships, draw conclusions, and reason within complex task material combining verbal, numerical, and figural information. Emphasis is placed on critical thinking and understanding the significance and limitations of data representation.

*Quantitative and formal problems* (QFP) assesses the ability to correctly handle numbers, quantities, units, and formulas. It involves solving short computational problems embedded in a medical or scientific context, focusing on the comprehension of formulas and variables rather than on actual calculation skills.

*Mental rotation task* (MRT) assesses spatial visualization and mental rotation abilities. Each task presents a transparent cube containing a twisted cable from the front view. In a second image, the same cube is shown from a different angle. Participants must recognize how the cube has been rotated.

*Figural memory task* (FMT) assesses the memorization of figural information in a short period of time. During a learning phase, similar but different figures are presented, each divided into five areas, one of which is colored. Following a retention period, participants are expected to recall and identify the colored area of the different figures from memory.

*Verbal memory task* (VMT) assesses the memorization of verbal information within a short period of time. During the learning phase, information about fictitious patients is presented, including their characteristics and diagnoses. Following a retention period, multiple-choice questions regarding the fictitious patients are to be answered from memory.

*Visual search task* (VST) assesses visual perception abilities and visual search. Multiple excerpts of a complex image are presented, each containing a small error, except for one. This task requires perceiving small details and differences in visual material.

**Supplementary Information 3**

*Description of tasks in the subtests of the HAM-Nat.*

*Knowledge test* (KT): This subtest contains knowledge questions of biology, chemistry, physics, and mathematics relevant to the medical field (e.g., In which part of the eye is the light refracted most strongly?).

*Verbal reasoning test* (VRT): In this task, participants are given a set of premises (e.g., City A is larger than city C; City B is smallest; City D is smaller than city A) and they have to deduce the correct answer to a corresponding question (e.g., Which is the biggest city?).

*Numerical reasoning test* (NRT): In this subtest respondents receive short descriptions of arithmetic relations (e.g., After a price reduction of 20 percent, product A costs four times as much as product B, which costs 20 euros. How much did product A cost before the price reduction?).

**Table 1**

*Reliability of the TMS and the HAM-Nat assessed by Cronbach´s alpha.*

|  |  | Test year | | | | |
| --- | --- | --- | --- | --- | --- | --- |
|  | Subtest | 2017 | 2018 | 2019 | 2020 | 2021 |
| TMS | VST | .58 | .52 | .54 | .49 - .61 | .61 - .69 |
|  | BMS | .71 | .70 | .67 | .68 - .73 | .70 - .73 |
|  | MRT | .74 | .72 | .75 | .72 - .76 | .77 - .80 |
|  | QFP | .75 | .75 | .70 | .67 - .77 | .73 - .76 |
|  | TC | .74 | .70 | .74 | .69 - .77 | .68 - .77 |
|  | FMT | .63 | .66 | .66 | .69 - .74 | .69 - .77 |
|  | VMT | .75 | .79 | .82 | .78 - .80 | .73 - .77 |
|  | DT | .72 | .70 | .68 | .65 - .75 | .64 - .69 |
|  | **Overall** | **.92** | **.91** | **.92** | **.91 - .93** | **.91** |
| HAM-Nat | **KT** | **-** | **-** | **-** | **.88 - .91** | **.90 - .91** |
|  | NRT | - | - | - | .40 - .43 | .36 - .55 |
|  | VRT | - | - | - | .40 - .44 | .49 - .62 |

*Note*. HAM-Nat test-takers prior to the test year of 2020 were not included in this study. Sample sizes of the TMS: *N_2017_* = 10449; *N_2018_* = 10067; *N_2019_* = 10864; *N_2020_* = 12913; *N_2021_* = 16126. Sample sizes of the HAM-Nat: *N_2020_* = 2772; *N_2021_* = 2404. Since 2020, both admission tests are administered on multipe days using different test versions. For the TMS, the reliability of the overall test score is of primary importance, as this score is used in the selection process. For the HAM-Nat, no overall test reliability is reported, as the test does not represent a homogeneous construct. For interpretation, particular attention should be given to the KT, which accounts for 75% of the total score.

**Table 2**

*Degree of range restriction in predictor variables.*

|  |  | Age | Gender | High-school GPA | Admission Test |
| --- | --- | --- | --- | --- | --- |
| TMS | PCGPA | 1.06 | 1.04 | 0.96 | 0.85 |
|  | M1 | 1.03 | 1.03 | 0.85 | 0.86 |
| HAM-Nat | PCGPA | 0.98 | 1.04 | 0.91 | 1.06 |
|  | M1 | 1.00 | 1.06 | 0.92 | 1.01 |

*Note*. The degree of range restriction was quantified by calculating the ratio of the standard deviation in the restricted sample to the standard deviation in the unrestricted sample per variable within the respective variance-covariance matrices.

**Table 3**

*Results of hierarchical regression analyses of TMS and HAM-Nat incumbents not controlling for age and gender.*

| Test | Model | Predictor | PCGPA | | | M1 | | |
| --- | --- | --- | --- | --- | --- | --- | --- | --- |
|  |  |  | β | *R^2^* | *∆R^2^* | β | *R^2^* | *∆R^2^* |
| TMS^a^ | 1a | GPA | .185** (.145**) | .034 (.021) |  | .265** (.207**) | .070 (.043) |  |
|  | 1b | TMS | -.227** (-.176**) | .051 (.031) |  | -.261** (-.181**) | .068 (.033) |  |
|  | 2 | GPA | .133** (.128**) |  | .017** (.016**)^c^ | .209** (.188*) |  | .040** (.035**)^c^ |
|  |  | TMS | -.190** (-.163**) | . 068 (.047) | .034** (.026**)^d^ | -.204** (-.159**) | .108 (.068) | .038** (.025**) ^d^ |
| HAM-Nat^b^ | 1a | GPA | .222** (.146**) | .049 (.021) |  | .354** (.283**) | .126 (.080) |  |
|  | 1b | HAM-Nat | -.355** (-.346**) | .126 (.120) |  | -.483** (-.463**) | .234 (.215) |  |
|  | 2 | GPA | .131** (.118**) |  | .016** (.014**)^c^ | .236** (.234**) |  | .051** (.054**)^c^ |
|  |  | HAM-Nat | -.318** (-.337**) | .142 (.134) | .093** (.113**) ^d^ | -.416** (-.437**) | .285 (.269) | .159** (.189**) ^d^ |

*Note*. Values in parentheses are not corrected for range restriction. *∆R^2^* = change in *R*^2^. β = standardized regression coefficient. ^a^*N_PCGPA_* = 1860 and *N_M1_* = 375. ^b^*N_PCGPA_* = 699 and *N_M1_* = 233. * indicates *p* < .05. ** indicates *p* < .01. ^c, d^ *∆R^2^* of model 2 is calculated over model 1a (^d^) and model 1b (^c^).
