# Supplementary material for: Predictive validity of admission tests and educational attainment on preclinical academic performance – a multisite study
Source: BMC Med Educ. 2025 Sep 23;25:1255. doi: 10.1186/s12909-025-07974-2 (PMC12455761; doi:10.1186/s12909-025-07974-2)
Supplement: Supplementary file 2 — Supplementary Material 2. [file 12909_2025_7974_MOESM2_ESM.html]

PredictiveValidity\_030625.knit


# Predictive Validity of Aptitude Tests and Educational Attainment on Preclinical Academic Performance – A Multisite Study

# 1 Introduction

For reasons of transparency and collaborative opportunities, the analysis code is shared and annotated briefly. We highly recommend reading the manuscript before checking or using the analysis code. However, the most important, non-self-explanatory terms are defined below.

Study participants are divided into *test-takers* (of either the TMS or HAM-Nat) and *incumbents*, which refers to test-takers who reported enrollment and provided at least one measure of study success. Two outcome variables were used. First, *PCGPA* which refers to the grade point average over the assessments during the first two preclinical years of undergraduate training. Second, *M1* which is the result of the first part of the medical licensing examination.

Due to data privacy restrictions of the student admission research consortium *stav*, data cannot be shared with external researchers. Requests to access this dataset should be directed to kontakt(at)projekt-stav.de

# 2 Descriptive statistics

## 2.1 Descriptive statistics of the total sample

```
#Number of participants
study_participants <- data %>% filter(!is.na(Gender) & !is.na(Age) & !is.na(GPA) & (!is.na(PCGPA) | !is.na(M1)) & (!is.na(TMS_total) | !is.na(HAMNat_total)))
nrow(study_participants)
```

```
## [1] 2113
```

```
#Year of application
table(study_participants$year_of_application)
```

```
## 
## 2017 2018 2019 2020 2021 2022 
##    6   44   66  556  851  590
```

```
round(prop.table(table(study_participants$year_of_application)), 4)
```

```
## 
##   2017   2018   2019   2020   2021   2022 
## 0.0028 0.0208 0.0312 0.2631 0.4027 0.2792
```

## 2.2 Descriptive statistics separated by TMS and HAM-Nat test-takers and incumbents

### 2.2.1 TMS

```
#Gender is coded with 1 = female, 2 = male, and 3 = gender-diverse.
psych::describe(data_TMS_Incumbents[, c('Gender', 'Age', 'GPA','TMS_total', 'PCGPA', 'M1')], fast = TRUE)
```

```
##           vars    n   mean   sd  min max range   se
## Gender       1 1880   1.31 0.47  1.0   3   2.0 0.01
## Age          2 1880  20.48 2.52 16.0  43  27.0 0.06
## GPA          3 1880   1.52 0.45  0.9   4   3.1 0.01
## TMS_total    4 1880 107.43 7.97 82.0 129  47.0 0.18
## PCGPA        5 1867   2.17 0.64  1.0   4   3.0 0.01
## M1           6  377   2.39 0.81  1.0   5   4.0 0.04
```

```
table(data_TMS_Incumbents$Gender)
```

```
## 
##    1    2    3 
## 1310  563    7
```

```
round(prop.table(table(data_TMS_Incumbents$Gender)), 4)
```

```
## 
##      1      2      3 
## 0.6968 0.2995 0.0037
```

```
psych::describe(data_TMS_Testtakers[, c('Gender', 'Age', 'GPA','TMS_total')], fast = TRUE)
```

```
##           vars    n   mean   sd  min max range   se
## Gender       1 8796   1.27 0.45  1.0   3   2.0 0.00
## Age          2 8796  20.77 2.37 16.0  52  36.0 0.03
## GPA          3 8796   1.70 0.47  0.9   4   3.1 0.00
## TMS_total    4 8796 102.47 9.43  0.0 129 129.0 0.10
```

```
table(data_TMS_Testtakers$Gender)
```

```
## 
##    1    2    3 
## 6447 2327   22
```

```
round(prop.table(table(data_TMS_Testtakers$Gender)), 4)
```

```
## 
##      1      2      3 
## 0.7329 0.2646 0.0025
```

```
#Excluding gender-diverse participants due to a insufficient sample size.
data_TMS_Incumbents <-(subset(data_TMS_Incumbents, Gender != 3))
data_TMS_Testtakers <-(subset(data_TMS_Testtakers, Gender != 3))

#Correlation matrices
rcorr(as.matrix(data_TMS_Incumbents[ , c('Gender', 'Age', 'GPA','TMS_total', 'PCGPA', 'M1')]))
```

```
##           Gender   Age   GPA TMS_total PCGPA    M1
## Gender      1.00  0.06  0.10      0.06 -0.09 -0.12
## Age         0.06  1.00  0.59     -0.16  0.12  0.18
## GPA         0.10  0.59  1.00     -0.10  0.14  0.21
## TMS_total   0.06 -0.16 -0.10      1.00 -0.18 -0.18
## PCGPA      -0.09  0.12  0.14     -0.18  1.00  0.64
## M1         -0.12  0.18  0.21     -0.18  0.64  1.00
## 
## n
##           Gender  Age  GPA TMS_total PCGPA  M1
## Gender      1873 1873 1873      1873  1860 375
## Age         1873 1873 1873      1873  1860 375
## GPA         1873 1873 1873      1873  1860 375
## TMS_total   1873 1873 1873      1873  1860 375
## PCGPA       1860 1860 1860      1860  1860 362
## M1           375  375  375       375   362 375
## 
## P
##           Gender Age    GPA    TMS_total PCGPA  M1    
## Gender           0.0071 0.0000 0.0157    0.0001 0.0183
## Age       0.0071        0.0000 0.0000    0.0000 0.0006
## GPA       0.0000 0.0000        0.0000    0.0000 0.0000
## TMS_total 0.0157 0.0000 0.0000           0.0000 0.0004
## PCGPA     0.0001 0.0000 0.0000 0.0000           0.0000
## M1        0.0183 0.0006 0.0000 0.0004    0.0000
```

```
rcorr(as.matrix(data_TMS_Testtakers[ , c('Gender', 'Age', 'GPA','TMS_total')]))
```

```
##           Gender   Age   GPA TMS_total
## Gender      1.00  0.05  0.03      0.09
## Age         0.05  1.00  0.46     -0.17
## GPA         0.03  0.46  1.00     -0.28
## TMS_total   0.09 -0.17 -0.28      1.00
## 
## n= 8774 
## 
## 
## P
##           Gender Age    GPA    TMS_total
## Gender           0.0000 0.0018 0.0000   
## Age       0.0000        0.0000 0.0000   
## GPA       0.0018 0.0000        0.0000   
## TMS_total 0.0000 0.0000 0.0000
```

### 2.2.2 HAM-Nat

```
#Gender is coded with 1 = female, 2 = male, and 3 = gender-diverse.
psych::describe(data_HAMNat_Incumbents[, c('Gender', 'Age', 'GPA','HAMNat_total', 'PCGPA', 'M1')], fast = TRUE)
```

```
##              vars   n  mean   sd   min  max range   se
## Gender          1 706  1.36 0.48  1.00  2.0  1.00 0.02
## Age             2 706 21.21 2.57 17.00 37.0 20.00 0.10
## GPA             3 706  1.63 0.43  0.90  3.4  2.50 0.02
## HAMNat_total    4 706  0.77 0.92 -2.63  2.9  5.52 0.03
## PCGPA           5 699  2.14 0.64  1.00  4.0  3.00 0.02
## M1              6 233  2.28 0.84  1.00  4.0  3.00 0.06
```

```
table(data_HAMNat_Incumbents$Gender)
```

```
## 
##   1   2 
## 455 251
```

```
round(prop.table(table(data_HAMNat_Incumbents$Gender)), 4)
```

```
## 
##      1      2 
## 0.6445 0.3555
```

```
psych::describe(data_HAMNat_Testtakers[, c('Gender', 'Age', 'GPA','HAMNat_total')], fast = TRUE)
```

```
##              vars    n  mean   sd   min  max range   se
## Gender          1 5020  1.31 0.47  1.00  3.0  2.00 0.01
## Age             2 5020 21.31 2.64 15.00 51.0 36.00 0.04
## GPA             3 5020  1.80 0.48  0.90  3.6  2.70 0.01
## HAMNat_total    4 5020  0.28 0.87 -2.99  2.9  5.89 0.01
```

```
table(data_HAMNat_Testtakers$Gender)
```

```
## 
##    1    2    3 
## 3486 1525    9
```

```
round(prop.table(table(data_HAMNat_Testtakers$Gender)), 4)
```

```
## 
##      1      2      3 
## 0.6944 0.3038 0.0018
```

```
#Excluding gender-diverse participants due to a insufficient sample size.
data_HAMNat_Incumbents <-(subset(data_HAMNat_Incumbents, Gender != 3))
data_HAMNat_Testtakers <-(subset(data_HAMNat_Testtakers, Gender != 3))

#Correlation matrices
rcorr(as.matrix(data_HAMNat_Incumbents[ , c('Gender', 'Age', 'GPA','HAMNat_total', 'PCGPA', 'M1')]))
```

```
##              Gender   Age   GPA HAMNat_total PCGPA    M1
## Gender         1.00  0.04  0.09         0.20 -0.03 -0.10
## Age            0.04  1.00  0.55        -0.06  0.07  0.24
## GPA            0.09  0.55  1.00        -0.08  0.15  0.28
## HAMNat_total   0.20 -0.06 -0.08         1.00 -0.35 -0.46
## PCGPA         -0.03  0.07  0.15        -0.35  1.00  0.69
## M1            -0.10  0.24  0.28        -0.46  0.69  1.00
## 
## n
##              Gender Age GPA HAMNat_total PCGPA  M1
## Gender          706 706 706          706   699 233
## Age             706 706 706          706   699 233
## GPA             706 706 706          706   699 233
## HAMNat_total    706 706 706          706   699 233
## PCGPA           699 699 699          699   699 226
## M1              233 233 233          233   226 233
## 
## P
##              Gender Age    GPA    HAMNat_total PCGPA  M1    
## Gender              0.3127 0.0140 0.0000       0.4283 0.1386
## Age          0.3127        0.0000 0.0917       0.0627 0.0003
## GPA          0.0140 0.0000        0.0317       0.0001 0.0000
## HAMNat_total 0.0000 0.0917 0.0317              0.0000 0.0000
## PCGPA        0.4283 0.0627 0.0001 0.0000              0.0000
## M1           0.1386 0.0003 0.0000 0.0000       0.0000
```

```
rcorr(as.matrix(data_HAMNat_Testtakers[ , c('Gender', 'Age', 'GPA','HAMNat_total')]))
```

```
##              Gender   Age   GPA HAMNat_total
## Gender         1.00  0.02  0.03         0.20
## Age            0.02  1.00  0.41        -0.05
## GPA            0.03  0.41  1.00        -0.28
## HAMNat_total   0.20 -0.05 -0.28         1.00
## 
## n= 5011 
## 
## 
## P
##              Gender Age    GPA    HAMNat_total
## Gender              0.1478 0.0622 0.0000      
## Age          0.1478        0.0000 0.0004      
## GPA          0.0622 0.0000        0.0000      
## HAMNat_total 0.0000 0.0004 0.0000
```

## 2.3 Differences in the sample of test-takers and incumbents

### 2.3.1 TMS

```
#Enrollment is coded with 1 = true (i.e., incumbents) and 2 = false (i.e., test-takers).
#Age
leveneTest(data_TMS_Testtakers$Age, data_TMS_Testtakers$Enrollment)
```

```
## Levene's Test for Homogeneity of Variance (center = median)
##         Df F value Pr(>F)
## group    1  2.0183 0.1554
##       8257
```

```
t.test(Age ~ Enrollment, var.equal = TRUE, alternative = "less", data = data_TMS_Testtakers)
```

```
## 
##  Two Sample t-test
## 
## data:  Age by Enrollment
## t = -6.9767, df = 8257, p-value = 1.628e-12
## alternative hypothesis: true difference in means between group 1 and group 2 is less than 0
## 95 percent confidence interval:
##        -Inf -0.3318706
## sample estimates:
## mean in group 1 mean in group 2 
##        20.47037        20.90464
```

```
data_TMS_Testtakers %>% cohens_d(Age ~ Enrollment, var.equal = TRUE)
```

```
## # A tibble: 1 × 7
##   .y.   group1 group2 effsize    n1    n2 magnitude 
## * <chr> <chr>  <chr>    <dbl> <int> <int> <ord>     
## 1 Age   1      2       -0.183  1873  6386 negligible
```

```
#High-school GPA
leveneTest(data_TMS_Testtakers$GPA, data_TMS_Testtakers$Enrollment)
```

```
## Levene's Test for Homogeneity of Variance (center = median)
##         Df F value Pr(>F)
## group    1  1.9626 0.1613
##       8257
```

```
t.test(GPA ~ Enrollment, var.equal = TRUE, alternative = "less", data = data_TMS_Testtakers)
```

```
## 
##  Two Sample t-test
## 
## data:  GPA by Enrollment
## t = -20.841, df = 8257, p-value < 2.2e-16
## alternative hypothesis: true difference in means between group 1 and group 2 is less than 0
## 95 percent confidence interval:
##        -Inf -0.2290912
## sample estimates:
## mean in group 1 mean in group 2 
##        1.523011        1.771735
```

```
data_TMS_Testtakers %>% cohens_d(GPA ~ Enrollment, var.equal = TRUE)
```

```
## # A tibble: 1 × 7
##   .y.   group1 group2 effsize    n1    n2 magnitude
## * <chr> <chr>  <chr>    <dbl> <int> <int> <ord>    
## 1 GPA   1      2       -0.548  1873  6386 moderate
```

```
#TMS score
leveneTest(data_TMS_Testtakers$TMS_total, data_TMS_Testtakers$Enrollment)
```

```
## Levene's Test for Homogeneity of Variance (center = median)
##         Df F value   Pr(>F)    
## group    1  47.773 5.14e-12 ***
##       8257                     
## ---
## Signif. codes:  0 '***' 0.001 '**' 0.01 '*' 0.05 '.' 0.1 ' ' 1
```

```
t.test(TMS_total ~ Enrollment, var.equal = FALSE, alternative = "greater", data = data_TMS_Testtakers)
```

```
## 
##  Welch Two Sample t-test
## 
## data:  TMS_total by Enrollment
## t = 31.028, df = 3480.4, p-value < 2.2e-16
## alternative hypothesis: true difference in means between group 1 and group 2 is greater than 0
## 95 percent confidence interval:
##  6.391978      Inf
## sample estimates:
## mean in group 1 mean in group 2 
##        107.4170        100.6671
```

```
data_TMS_Testtakers %>% cohens_d(TMS_total ~ Enrollment, var.equal = FALSE)
```

```
## # A tibble: 1 × 7
##   .y.       group1 group2 effsize    n1    n2 magnitude
## * <chr>     <chr>  <chr>    <dbl> <int> <int> <ord>    
## 1 TMS_total 1      2        0.782  1873  6386 moderate
```

```
#Gender
chisq.test(data_TMS_Testtakers$Gender, data_TMS_Testtakers$Enrollment)
```

```
## 
##  Pearson's Chi-squared test with Yates' continuity correction
## 
## data:  data_TMS_Testtakers$Gender and data_TMS_Testtakers$Enrollment
## X-squared = 14.71, df = 1, p-value = 0.0001254
```

```
cramerV(data_TMS_Testtakers$Gender, data_TMS_Testtakers$Enrollment)
```

```
## Cramer V 
##  0.04126
```

### 2.3.2 HAM-Nat

```
#Enrollment is coded with 1 = true (i.e., incumbents) and 2 = false (i.e., test-takers).
#Age
leveneTest(data_HAMNat_Testtakers$Age, data_HAMNat_Testtakers$Enrollment)
```

```
## Levene's Test for Homogeneity of Variance (center = median)
##         Df F value Pr(>F)
## group    1  1.0734 0.3002
##       4862
```

```
t.test(Age ~ Enrollment, var.equal = TRUE, alternative = "less", data = data_HAMNat_Testtakers)
```

```
## 
##  Two Sample t-test
## 
## data:  Age by Enrollment
## t = -1.0637, df = 4862, p-value = 0.1438
## alternative hypothesis: true difference in means between group 1 and group 2 is less than 0
## 95 percent confidence interval:
##       -Inf 0.0618495
## sample estimates:
## mean in group 1 mean in group 2 
##        21.21105        21.32419
```

```
data_HAMNat_Testtakers %>% cohens_d(Age ~ Enrollment, var.equal = TRUE)
```

```
## # A tibble: 1 × 7
##   .y.   group1 group2 effsize    n1    n2 magnitude 
## * <chr> <chr>  <chr>    <dbl> <int> <int> <ord>     
## 1 Age   1      2      -0.0433   706  4158 negligible
```

```
#High-school GPA
leveneTest(data_HAMNat_Testtakers$GPA, data_HAMNat_Testtakers$Enrollment)
```

```
## Levene's Test for Homogeneity of Variance (center = median)
##         Df F value    Pr(>F)    
## group    1  11.741 0.0006163 ***
##       4862                      
## ---
## Signif. codes:  0 '***' 0.001 '**' 0.01 '*' 0.05 '.' 0.1 ' ' 1
```

```
t.test(GPA ~ Enrollment, var.equal = FALSE, alternative = "less", data = data_HAMNat_Testtakers)
```

```
## 
##  Welch Two Sample t-test
## 
## data:  GPA by Enrollment
## t = -11.778, df = 1014.6, p-value < 2.2e-16
## alternative hypothesis: true difference in means between group 1 and group 2 is less than 0
## 95 percent confidence interval:
##       -Inf -0.181961
## sample estimates:
## mean in group 1 mean in group 2 
##        1.628754        1.840284
```

```
data_HAMNat_Testtakers %>% cohens_d(GPA ~ Enrollment, var.equal = FALSE)
```

```
## # A tibble: 1 × 7
##   .y.   group1 group2 effsize    n1    n2 magnitude
## * <chr> <chr>  <chr>    <dbl> <int> <int> <ord>    
## 1 GPA   1      2       -0.464   706  4158 small
```

```
#HAM-Nat score
leveneTest(data_HAMNat_Testtakers$HAMNat_total, data_HAMNat_Testtakers$Enrollment)
```

```
## Levene's Test for Homogeneity of Variance (center = median)
##         Df F value    Pr(>F)    
## group    1   24.89 6.282e-07 ***
##       4862                      
## ---
## Signif. codes:  0 '***' 0.001 '**' 0.01 '*' 0.05 '.' 0.1 ' ' 1
```

```
t.test(HAMNat_total ~ Enrollment, var.equal = FALSE, alternative = "greater", data = data_HAMNat_Testtakers)
```

```
## 
##  Welch Two Sample t-test
## 
## data:  HAMNat_total by Enrollment
## t = 16.135, df = 912.23, p-value < 2.2e-16
## alternative hypothesis: true difference in means between group 1 and group 2 is greater than 0
## 95 percent confidence interval:
##  0.5329499       Inf
## sample estimates:
## mean in group 1 mean in group 2 
##       0.7747497       0.1812355
```

```
data_HAMNat_Testtakers %>% cohens_d(HAMNat_total ~ Enrollment, var.equal = FALSE)
```

```
## # A tibble: 1 × 7
##   .y.          group1 group2 effsize    n1    n2 magnitude
## * <chr>        <chr>  <chr>    <dbl> <int> <int> <ord>    
## 1 HAMNat_total 1      2        0.679   706  4158 moderate
```

```
#Gender
chisq.test(data_HAMNat_Testtakers$Gender, data_HAMNat_Testtakers$Enrollment)
```

```
## 
##  Pearson's Chi-squared test with Yates' continuity correction
## 
## data:  data_HAMNat_Testtakers$Gender and data_HAMNat_Testtakers$Enrollment
## X-squared = 9.2987, df = 1, p-value = 0.002293
```

```
cramerV(data_HAMNat_Testtakers$Gender, data_HAMNat_Testtakers$Enrollment)
```

```
## Cramer V 
##   0.0437
```

# 3 Regression Analyses

## 3.1 Regression analyses without correction for effects of range restriction

### 3.1.1 TMS

```
#Criterion: PCGPA

#Models
lm_TMS_1 <- lm(PCGPA ~ Gender + Age, data = data_TMS_Incumbents)
lm_TMS_2a <- lm(PCGPA ~ Gender + Age + GPA, data = data_TMS_Incumbents)
lm_TMS_2b <- lm(PCGPA ~ Gender + Age + TMS_total, data = data_TMS_Incumbents)
lm_TMS_3 <- lm(PCGPA ~ Gender + Age + GPA + TMS_total, data = data_TMS_Incumbents)

#Results
summary(lm_TMS_1)
```

```
## 
## Call:
## lm(formula = PCGPA ~ Gender + Age, data = data_TMS_Incumbents)
## 
## Residuals:
##      Min       1Q   Median       3Q      Max 
## -1.72981 -0.43379 -0.09653  0.40937  1.86621 
## 
## Coefficients:
##              Estimate Std. Error t value Pr(>|t|)    
## (Intercept)  1.705443   0.125536  13.585  < 2e-16 ***
## Gender      -0.136305   0.032150  -4.240 2.35e-05 ***
## Age          0.031369   0.005866   5.347 1.00e-07 ***
## ---
## Signif. codes:  0 '***' 0.001 '**' 0.01 '*' 0.05 '.' 0.1 ' ' 1
## 
## Residual standard error: 0.6351 on 1857 degrees of freedom
##   (13 Beobachtungen als fehlend gelöscht)
## Multiple R-squared:  0.02309,    Adjusted R-squared:  0.02204 
## F-statistic: 21.95 on 2 and 1857 DF,  p-value: 3.797e-10
```

```
summary(lm_TMS_2a)
```

```
## 
## Call:
## lm(formula = PCGPA ~ Gender + Age + GPA, data = data_TMS_Incumbents)
## 
## Residuals:
##      Min       1Q   Median       3Q      Max 
## -1.72401 -0.45387 -0.09312  0.41270  1.84397 
## 
## Coefficients:
##              Estimate Std. Error t value Pr(>|t|)    
## (Intercept)  1.832711   0.128116  14.305  < 2e-16 ***
## Gender      -0.147989   0.032095  -4.611 4.28e-06 ***
## Age          0.012371   0.007224   1.712    0.087 .  
## GPA          0.181734   0.040725   4.462 8.59e-06 ***
## ---
## Signif. codes:  0 '***' 0.001 '**' 0.01 '*' 0.05 '.' 0.1 ' ' 1
## 
## Residual standard error: 0.6319 on 1856 degrees of freedom
##   (13 Beobachtungen als fehlend gelöscht)
## Multiple R-squared:  0.03346,    Adjusted R-squared:  0.0319 
## F-statistic: 21.42 on 3 and 1856 DF,  p-value: 1.226e-13
```

```
summary(lm_TMS_2b)
```

```
## 
## Call:
## lm(formula = PCGPA ~ Gender + Age + TMS_total, data = data_TMS_Incumbents)
## 
## Residuals:
##      Min       1Q   Median       3Q      Max 
## -1.52258 -0.42823 -0.09179  0.40834  1.92407 
## 
## Coefficients:
##              Estimate Std. Error t value Pr(>|t|)    
## (Intercept)  3.160364   0.249324  12.676  < 2e-16 ***
## Gender      -0.121259   0.031853  -3.807 0.000145 ***
## Age          0.024821   0.005879   4.222 2.54e-05 ***
## TMS_total   -0.012478   0.001855  -6.728 2.29e-11 ***
## ---
## Signif. codes:  0 '***' 0.001 '**' 0.01 '*' 0.05 '.' 0.1 ' ' 1
## 
## Residual standard error: 0.6277 on 1856 degrees of freedom
##   (13 Beobachtungen als fehlend gelöscht)
## Multiple R-squared:  0.04635,    Adjusted R-squared:  0.04481 
## F-statistic: 30.07 on 3 and 1856 DF,  p-value: < 2.2e-16
```

```
summary(lm_TMS_3)
```

```
## 
## Call:
## lm(formula = PCGPA ~ Gender + Age + GPA + TMS_total, data = data_TMS_Incumbents)
## 
## Residuals:
##      Min       1Q   Median       3Q      Max 
## -1.51844 -0.44050 -0.08705  0.40149  1.89607 
## 
## Coefficients:
##              Estimate Std. Error t value Pr(>|t|)    
## (Intercept)  3.274543   0.249408  13.129  < 2e-16 ***
## Gender      -0.132858   0.031801  -4.178 3.08e-05 ***
## Age          0.006195   0.007199   0.861     0.39    
## GPA          0.178644   0.040253   4.438 9.61e-06 ***
## TMS_total   -0.012384   0.001846  -6.710 2.57e-11 ***
## ---
## Signif. codes:  0 '***' 0.001 '**' 0.01 '*' 0.05 '.' 0.1 ' ' 1
## 
## Residual standard error: 0.6246 on 1855 degrees of freedom
##   (13 Beobachtungen als fehlend gelöscht)
## Multiple R-squared:  0.05637,    Adjusted R-squared:  0.05433 
## F-statistic:  27.7 on 4 and 1855 DF,  p-value: < 2.2e-16
```

```
lm.beta(lm_TMS_1)
```

```
## 
## Call:
## lm(formula = PCGPA ~ Gender + Age, data = data_TMS_Incumbents)
## 
## Standardized Coefficients::
## (Intercept)      Gender         Age 
##          NA -0.09743258  0.12288789
```

```
lm.beta(lm_TMS_2a)
```

```
## 
## Call:
## lm(formula = PCGPA ~ Gender + Age + GPA, data = data_TMS_Incumbents)
## 
## Standardized Coefficients::
## (Intercept)      Gender         Age         GPA 
##          NA -0.10578452  0.04846357  0.12671626
```

```
lm.beta(lm_TMS_2b)
```

```
## 
## Call:
## lm(formula = PCGPA ~ Gender + Age + TMS_total, data = data_TMS_Incumbents)
## 
## Standardized Coefficients::
## (Intercept)      Gender         Age   TMS_total 
##          NA -0.08667757  0.09723666 -0.15490131
```

```
lm.beta(lm_TMS_3)
```

```
## 
## Call:
## lm(formula = PCGPA ~ Gender + Age + GPA + TMS_total, data = data_TMS_Incumbents)
## 
## Standardized Coefficients::
## (Intercept)      Gender         Age         GPA   TMS_total 
##          NA -0.09496828  0.02427030  0.12456185 -0.15373815
```

```
anova(lm_TMS_1, lm_TMS_2a, lm_TMS_3)
```

```
## Analysis of Variance Table
## 
## Model 1: PCGPA ~ Gender + Age
## Model 2: PCGPA ~ Gender + Age + GPA
## Model 3: PCGPA ~ Gender + Age + GPA + TMS_total
##   Res.Df    RSS Df Sum of Sq      F    Pr(>F)    
## 1   1857 749.08                                  
## 2   1856 741.13  1    7.9518 20.386 6.728e-06 ***
## 3   1855 723.57  1   17.5629 45.026 2.575e-11 ***
## ---
## Signif. codes:  0 '***' 0.001 '**' 0.01 '*' 0.05 '.' 0.1 ' ' 1
```

```
anova(lm_TMS_1, lm_TMS_2b, lm_TMS_3)
```

```
## Analysis of Variance Table
## 
## Model 1: PCGPA ~ Gender + Age
## Model 2: PCGPA ~ Gender + Age + TMS_total
## Model 3: PCGPA ~ Gender + Age + GPA + TMS_total
##   Res.Df    RSS Df Sum of Sq      F    Pr(>F)    
## 1   1857 749.08                                  
## 2   1856 731.25  1   17.8320 45.716 1.826e-11 ***
## 3   1855 723.57  1    7.6827 19.696 9.613e-06 ***
## ---
## Signif. codes:  0 '***' 0.001 '**' 0.01 '*' 0.05 '.' 0.1 ' ' 1
```

```
#Criterion: M1

#Models
lm_TMS_M1_1 <- lm(M1 ~ Gender + Age, data = data_TMS_Incumbents)
lm_TMS_M1_2a <- lm(M1 ~ Gender + Age + GPA, data = data_TMS_Incumbents)
lm_TMS_M1_2b <- lm(M1 ~ Gender + Age + TMS_total, data = data_TMS_Incumbents)
lm_TMS_M1_3 <- lm(M1 ~ Gender + Age + GPA + TMS_total, data = data_TMS_Incumbents)

#Results
summary(lm_TMS_M1_1)
```

```
## 
## Call:
## lm(formula = M1 ~ Gender + Age, data = data_TMS_Incumbents)
## 
## Residuals:
##      Min       1Q   Median       3Q      Max 
## -1.55701 -0.55701  0.00301  0.56302  2.62304 
## 
## Coefficients:
##             Estimate Std. Error t value Pr(>|t|)    
## (Intercept)  1.46618    0.35863   4.088 5.33e-05 ***
## Gender      -0.22953    0.09003  -2.550   0.0112 *  
## Age          0.06002    0.01680   3.573   0.0004 ***
## ---
## Signif. codes:  0 '***' 0.001 '**' 0.01 '*' 0.05 '.' 0.1 ' ' 1
## 
## Residual standard error: 0.7931 on 372 degrees of freedom
##   (1498 Beobachtungen als fehlend gelöscht)
## Multiple R-squared:  0.04751,    Adjusted R-squared:  0.04239 
## F-statistic: 9.278 on 2 and 372 DF,  p-value: 0.000117
```

```
summary(lm_TMS_M1_2a)
```

```
## 
## Call:
## lm(formula = M1 ~ Gender + Age + GPA, data = data_TMS_Incumbents)
## 
## Residuals:
##      Min       1Q   Median       3Q      Max 
## -1.62231 -0.59105 -0.01738  0.57135  2.65241 
## 
## Coefficients:
##             Estimate Std. Error t value Pr(>|t|)    
## (Intercept)  1.70498    0.36588   4.660 4.42e-06 ***
## Gender      -0.25673    0.08978  -2.859  0.00448 ** 
## Age          0.02206    0.02160   1.021  0.30773    
## GPA          0.36933    0.13391   2.758  0.00610 ** 
## ---
## Signif. codes:  0 '***' 0.001 '**' 0.01 '*' 0.05 '.' 0.1 ' ' 1
## 
## Residual standard error: 0.7861 on 371 degrees of freedom
##   (1498 Beobachtungen als fehlend gelöscht)
## Multiple R-squared:  0.06665,    Adjusted R-squared:  0.0591 
## F-statistic: 8.831 on 3 and 371 DF,  p-value: 1.144e-05
```

```
summary(lm_TMS_M1_2b)
```

```
## 
## Call:
## lm(formula = M1 ~ Gender + Age + TMS_total, data = data_TMS_Incumbents)
## 
## Residuals:
##      Min       1Q   Median       3Q      Max 
## -1.59396 -0.59744  0.00334  0.59383  2.64363 
## 
## Coefficients:
##              Estimate Std. Error t value Pr(>|t|)    
## (Intercept)  3.421643   0.671392   5.096 5.53e-07 ***
## Gender      -0.244043   0.088857  -2.746 0.006318 ** 
## Age          0.054412   0.016642   3.270 0.001178 ** 
## TMS_total   -0.017176   0.005013  -3.426 0.000681 ***
## ---
## Signif. codes:  0 '***' 0.001 '**' 0.01 '*' 0.05 '.' 0.1 ' ' 1
## 
## Residual standard error: 0.7819 on 371 degrees of freedom
##   (1498 Beobachtungen als fehlend gelöscht)
## Multiple R-squared:  0.07672,    Adjusted R-squared:  0.06926 
## F-statistic: 10.28 on 3 and 371 DF,  p-value: 1.632e-06
```

```
summary(lm_TMS_M1_3)
```

```
## 
## Call:
## lm(formula = M1 ~ Gender + Age + GPA + TMS_total, data = data_TMS_Incumbents)
## 
## Residuals:
##      Min       1Q   Median       3Q      Max 
## -1.57633 -0.60591  0.01654  0.60641  2.66975 
## 
## Coefficients:
##              Estimate Std. Error t value Pr(>|t|)    
## (Intercept)  3.546989   0.668132   5.309 1.91e-07 ***
## Gender      -0.268457   0.088699  -3.027  0.00265 ** 
## Age          0.019637   0.021336   0.920  0.35799    
## GPA          0.341066   0.132466   2.575  0.01042 *  
## TMS_total   -0.016340   0.004986  -3.277  0.00115 ** 
## ---
## Signif. codes:  0 '***' 0.001 '**' 0.01 '*' 0.05 '.' 0.1 ' ' 1
## 
## Residual standard error: 0.776 on 370 degrees of freedom
##   (1498 Beobachtungen als fehlend gelöscht)
## Multiple R-squared:  0.09297,    Adjusted R-squared:  0.08317 
## F-statistic: 9.481 on 4 and 370 DF,  p-value: 2.629e-07
```

```
lm.beta(lm_TMS_M1_1)
```

```
## 
## Call:
## lm(formula = M1 ~ Gender + Age, data = data_TMS_Incumbents)
## 
## Standardized Coefficients::
## (Intercept)      Gender         Age 
##          NA  -0.1291155   0.1809318
```

```
lm.beta(lm_TMS_M1_2a)
```

```
## 
## Call:
## lm(formula = M1 ~ Gender + Age + GPA, data = data_TMS_Incumbents)
## 
## Standardized Coefficients::
## (Intercept)      Gender         Age         GPA 
##          NA -0.14441749  0.06651478  0.18057005
```

```
lm.beta(lm_TMS_M1_2b)
```

```
## 
## Call:
## lm(formula = M1 ~ Gender + Age + TMS_total, data = data_TMS_Incumbents)
## 
## Standardized Coefficients::
## (Intercept)      Gender         Age   TMS_total 
##          NA  -0.1372794   0.1640365  -0.1719738
```

```
lm.beta(lm_TMS_M1_3)
```

```
## 
## Call:
## lm(formula = M1 ~ Gender + Age + GPA + TMS_total, data = data_TMS_Incumbents)
## 
## Standardized Coefficients::
## (Intercept)      Gender         Age         GPA   TMS_total 
##          NA -0.15101284  0.05919872  0.16675012 -0.16360336
```

```
anova(lm_TMS_M1_1, lm_TMS_M1_2a, lm_TMS_M1_3)
```

```
## Analysis of Variance Table
## 
## Model 1: M1 ~ Gender + Age
## Model 2: M1 ~ Gender + Age + GPA
## Model 3: M1 ~ Gender + Age + GPA + TMS_total
##   Res.Df    RSS Df Sum of Sq       F   Pr(>F)   
## 1    372 233.99                                 
## 2    371 229.28  1    4.7014  7.8069 0.005476 **
## 3    370 222.82  1    6.4670 10.7387 0.001148 **
## ---
## Signif. codes:  0 '***' 0.001 '**' 0.01 '*' 0.05 '.' 0.1 ' ' 1
```

```
anova(lm_TMS_M1_1, lm_TMS_M1_2b, lm_TMS_M1_3)
```

```
## Analysis of Variance Table
## 
## Model 1: M1 ~ Gender + Age
## Model 2: M1 ~ Gender + Age + TMS_total
## Model 3: M1 ~ Gender + Age + GPA + TMS_total
##   Res.Df    RSS Df Sum of Sq       F    Pr(>F)    
## 1    372 233.99                                   
## 2    371 226.81  1    7.1761 11.9162 0.0006208 ***
## 3    370 222.82  1    3.9923  6.6294 0.0104190 *  
## ---
## Signif. codes:  0 '***' 0.001 '**' 0.01 '*' 0.05 '.' 0.1 ' ' 1
```

### 3.1.2 HAM-Nat

```
#Criterion: PCGPA

#Models
lm_HAMNat_1 <- lm(PCGPA ~ Gender + Age, data = data_HAMNat_Incumbents)
lm_HAMNat_2a <- lm(PCGPA ~ Gender + Age + GPA, data = data_HAMNat_Incumbents)
lm_HAMNat_2b <- lm(PCGPA ~ Gender + Age + HAMNat_total, data = data_HAMNat_Incumbents)
lm_HAMNat_3 <- lm(PCGPA ~ Gender + Age + GPA + HAMNat_total, data = data_HAMNat_Incumbents)

#Results
summary(lm_HAMNat_1)
```

```
## 
## Call:
## lm(formula = PCGPA ~ Gender + Age, data = data_HAMNat_Incumbents)
## 
## Residuals:
##     Min      1Q  Median      3Q     Max 
## -1.2776 -0.4356 -0.1098  0.3999  1.8289 
## 
## Coefficients:
##              Estimate Std. Error t value Pr(>|t|)    
## (Intercept)  1.824051   0.208684   8.741   <2e-16 ***
## Gender      -0.043536   0.050168  -0.868   0.3858    
## Age          0.017754   0.009361   1.897   0.0583 .  
## ---
## Signif. codes:  0 '***' 0.001 '**' 0.01 '*' 0.05 '.' 0.1 ' ' 1
## 
## Residual standard error: 0.6347 on 696 degrees of freedom
##   (7 Beobachtungen als fehlend gelöscht)
## Multiple R-squared:  0.006037,   Adjusted R-squared:  0.003181 
## F-statistic: 2.114 on 2 and 696 DF,  p-value: 0.1216
```

```
summary(lm_HAMNat_2a)
```

```
## 
## Call:
## lm(formula = PCGPA ~ Gender + Age + GPA, data = data_HAMNat_Incumbents)
## 
## Residuals:
##     Min      1Q  Median      3Q     Max 
## -1.2707 -0.4631 -0.0981  0.4061  1.8935 
## 
## Coefficients:
##              Estimate Std. Error t value Pr(>|t|)    
## (Intercept)  1.917613   0.208728   9.187  < 2e-16 ***
## Gender      -0.058175   0.049941  -1.165 0.244467    
## Age         -0.003489   0.011089  -0.315 0.753130    
## GPA          0.231320   0.066011   3.504 0.000487 ***
## ---
## Signif. codes:  0 '***' 0.001 '**' 0.01 '*' 0.05 '.' 0.1 ' ' 1
## 
## Residual standard error: 0.6296 on 695 degrees of freedom
##   (7 Beobachtungen als fehlend gelöscht)
## Multiple R-squared:  0.02329,    Adjusted R-squared:  0.01908 
## F-statistic: 5.525 on 3 and 695 DF,  p-value: 0.0009418
```

```
summary(lm_HAMNat_2b)
```

```
## 
## Call:
## lm(formula = PCGPA ~ Gender + Age + HAMNat_total, data = data_HAMNat_Incumbents)
## 
## Residuals:
##      Min       1Q   Median       3Q      Max 
## -1.34885 -0.42914 -0.05229  0.41633  1.68216 
## 
## Coefficients:
##               Estimate Std. Error t value Pr(>|t|)    
## (Intercept)   2.019693   0.197122  10.246   <2e-16 ***
## Gender        0.050308   0.048127   1.045    0.296    
## Age           0.011477   0.008819   1.301    0.194    
## HAMNat_total -0.243363   0.025186  -9.662   <2e-16 ***
## ---
## Signif. codes:  0 '***' 0.001 '**' 0.01 '*' 0.05 '.' 0.1 ' ' 1
## 
## Residual standard error: 0.5963 on 695 degrees of freedom
##   (7 Beobachtungen als fehlend gelöscht)
## Multiple R-squared:  0.1237, Adjusted R-squared:   0.12 
## F-statistic: 32.72 on 3 and 695 DF,  p-value: < 2.2e-16
```

```
summary(lm_HAMNat_3)
```

```
## 
## Call:
## lm(formula = PCGPA ~ Gender + Age + GPA + HAMNat_total, data = data_HAMNat_Incumbents)
## 
## Residuals:
##      Min       1Q   Median       3Q      Max 
## -1.34156 -0.42910 -0.05917  0.41644  1.67121 
## 
## Coefficients:
##               Estimate Std. Error t value Pr(>|t|)    
## (Intercept)   2.090335   0.197434  10.587  < 2e-16 ***
## Gender        0.036315   0.048085   0.755  0.45037    
## Age          -0.005463   0.010446  -0.523  0.60116    
## GPA           0.186079   0.062356   2.984  0.00294 ** 
## HAMNat_total -0.237614   0.025118  -9.460  < 2e-16 ***
## ---
## Signif. codes:  0 '***' 0.001 '**' 0.01 '*' 0.05 '.' 0.1 ' ' 1
## 
## Residual standard error: 0.593 on 694 degrees of freedom
##   (7 Beobachtungen als fehlend gelöscht)
## Multiple R-squared:  0.1349, Adjusted R-squared:  0.1299 
## F-statistic: 27.04 on 4 and 694 DF,  p-value: < 2.2e-16
```

```
lm.beta(lm_HAMNat_1)
```

```
## 
## Call:
## lm(formula = PCGPA ~ Gender + Age, data = data_HAMNat_Incumbents)
## 
## Standardized Coefficients::
## (Intercept)      Gender         Age 
##          NA -0.03282007  0.07172846
```

```
lm.beta(lm_HAMNat_2a)
```

```
## 
## Call:
## lm(formula = PCGPA ~ Gender + Age + GPA, data = data_HAMNat_Incumbents)
## 
## Standardized Coefficients::
## (Intercept)      Gender         Age         GPA 
##          NA -0.04385613 -0.01409691  0.15754123
```

```
lm.beta(lm_HAMNat_2b)
```

```
## 
## Call:
## lm(formula = PCGPA ~ Gender + Age + HAMNat_total, data = data_HAMNat_Incumbents)
## 
## Standardized Coefficients::
##  (Intercept)       Gender          Age HAMNat_total 
##           NA   0.03792524   0.04636843  -0.35102601
```

```
lm.beta(lm_HAMNat_3)
```

```
## 
## Call:
## lm(formula = PCGPA ~ Gender + Age + GPA + HAMNat_total, data = data_HAMNat_Incumbents)
## 
## Standardized Coefficients::
##  (Intercept)       Gender          Age          GPA HAMNat_total 
##           NA   0.02737638  -0.02207231   0.12672964  -0.34273374
```

```
anova(lm_HAMNat_1, lm_HAMNat_2a, lm_HAMNat_3)
```

```
## Analysis of Variance Table
## 
## Model 1: PCGPA ~ Gender + Age
## Model 2: PCGPA ~ Gender + Age + GPA
## Model 3: PCGPA ~ Gender + Age + GPA + HAMNat_total
##   Res.Df    RSS Df Sum of Sq      F    Pr(>F)    
## 1    696 280.36                                  
## 2    695 275.50  1    4.8677 13.843 0.0002148 ***
## 3    694 244.03  1   31.4663 89.487 < 2.2e-16 ***
## ---
## Signif. codes:  0 '***' 0.001 '**' 0.01 '*' 0.05 '.' 0.1 ' ' 1
```

```
anova(lm_HAMNat_1, lm_HAMNat_2b, lm_HAMNat_3)
```

```
## Analysis of Variance Table
## 
## Model 1: PCGPA ~ Gender + Age
## Model 2: PCGPA ~ Gender + Age + HAMNat_total
## Model 3: PCGPA ~ Gender + Age + GPA + HAMNat_total
##   Res.Df    RSS Df Sum of Sq       F    Pr(>F)    
## 1    696 280.36                                   
## 2    695 247.16  1    33.203 94.4255 < 2.2e-16 ***
## 3    694 244.03  1     3.131  8.9052  0.002943 ** 
## ---
## Signif. codes:  0 '***' 0.001 '**' 0.01 '*' 0.05 '.' 0.1 ' ' 1
```

```
#Criterion: M1

#Models
lm_HAMNat_M1_1 <- lm(M1 ~ Gender + Age, data = data_HAMNat_Incumbents)
lm_HAMNat_M1_2a <- lm(M1 ~ Gender + Age + GPA, data = data_HAMNat_Incumbents)
lm_HAMNat_M1_2b <- lm(M1 ~ Gender + Age + HAMNat_total, data = data_HAMNat_Incumbents)
lm_HAMNat_M1_3 <- lm(M1 ~ Gender + Age + GPA + HAMNat_total, data = data_HAMNat_Incumbents)

#Results
summary(lm_HAMNat_M1_1)
```

```
## 
## Call:
## lm(formula = M1 ~ Gender + Age, data = data_HAMNat_Incumbents)
## 
## Residuals:
##     Min      1Q  Median      3Q     Max 
## -1.7526 -0.6477 -0.1477  0.5912  2.0116 
## 
## Coefficients:
##             Estimate Std. Error t value Pr(>|t|)    
## (Intercept)  0.88922    0.44851   1.983 0.048599 *  
## Gender      -0.20681    0.11043  -1.873 0.062374 .  
## Age          0.07962    0.02050   3.885 0.000134 ***
## ---
## Signif. codes:  0 '***' 0.001 '**' 0.01 '*' 0.05 '.' 0.1 ' ' 1
## 
## Residual standard error: 0.8174 on 230 degrees of freedom
##   (473 Beobachtungen als fehlend gelöscht)
## Multiple R-squared:  0.07046,    Adjusted R-squared:  0.06238 
## F-statistic: 8.718 on 2 and 230 DF,  p-value: 0.0002242
```

```
summary(lm_HAMNat_M1_2a)
```

```
## 
## Call:
## lm(formula = M1 ~ Gender + Age + GPA, data = data_HAMNat_Incumbents)
## 
## Residuals:
##      Min       1Q   Median       3Q      Max 
## -1.71681 -0.63011 -0.08558  0.61826  2.09255 
## 
## Coefficients:
##             Estimate Std. Error t value Pr(>|t|)   
## (Intercept)  1.08021    0.44543   2.425  0.01608 * 
## Gender      -0.22828    0.10879  -2.098  0.03696 * 
## Age          0.03747    0.02455   1.526  0.12832   
## GPA          0.44532    0.14819   3.005  0.00295 **
## ---
## Signif. codes:  0 '***' 0.001 '**' 0.01 '*' 0.05 '.' 0.1 ' ' 1
## 
## Residual standard error: 0.8035 on 229 degrees of freedom
##   (473 Beobachtungen als fehlend gelöscht)
## Multiple R-squared:  0.1057, Adjusted R-squared:  0.09401 
## F-statistic: 9.025 on 3 and 229 DF,  p-value: 1.132e-05
```

```
summary(lm_HAMNat_M1_2b)
```

```
## 
## Call:
## lm(formula = M1 ~ Gender + Age + HAMNat_total, data = data_HAMNat_Incumbents)
## 
## Residuals:
##      Min       1Q   Median       3Q      Max 
## -1.60131 -0.54350 -0.04737  0.53397  2.16261 
## 
## Coefficients:
##              Estimate Std. Error t value Pr(>|t|)    
## (Intercept)   1.40624    0.40896   3.439 0.000695 ***
## Gender       -0.04032    0.10171  -0.396 0.692175    
## Age           0.06314    0.01855   3.403 0.000786 ***
## HAMNat_total -0.42216    0.05652  -7.470 1.68e-12 ***
## ---
## Signif. codes:  0 '***' 0.001 '**' 0.01 '*' 0.05 '.' 0.1 ' ' 1
## 
## Residual standard error: 0.7346 on 229 degrees of freedom
##   (473 Beobachtungen als fehlend gelöscht)
## Multiple R-squared:  0.2526, Adjusted R-squared:  0.2428 
## F-statistic: 25.79 on 3 and 229 DF,  p-value: 2.06e-14
```

```
summary(lm_HAMNat_M1_3)
```

```
## 
## Call:
## lm(formula = M1 ~ Gender + Age + GPA + HAMNat_total, data = data_HAMNat_Incumbents)
## 
## Residuals:
##      Min       1Q   Median       3Q      Max 
## -1.57799 -0.55069 -0.02762  0.50075  2.04989 
## 
## Coefficients:
##              Estimate Std. Error t value Pr(>|t|)    
## (Intercept)   1.54485    0.40681   3.798 0.000187 ***
## Gender       -0.06283    0.10071  -0.624 0.533325    
## Age           0.02953    0.02217   1.332 0.184194    
## GPA           0.36037    0.13418   2.686 0.007772 ** 
## HAMNat_total -0.40914    0.05598  -7.309  4.5e-12 ***
## ---
## Signif. codes:  0 '***' 0.001 '**' 0.01 '*' 0.05 '.' 0.1 ' ' 1
## 
## Residual standard error: 0.7248 on 228 degrees of freedom
##   (473 Beobachtungen als fehlend gelöscht)
## Multiple R-squared:  0.2755, Adjusted R-squared:  0.2628 
## F-statistic: 21.67 on 4 and 228 DF,  p-value: 3.595e-15
```

```
lm.beta(lm_HAMNat_M1_1)
```

```
## 
## Call:
## lm(formula = M1 ~ Gender + Age, data = data_HAMNat_Incumbents)
## 
## Standardized Coefficients::
## (Intercept)      Gender         Age 
##          NA  -0.1195348   0.2479617
```

```
lm.beta(lm_HAMNat_M1_2a)
```

```
## 
## Call:
## lm(formula = M1 ~ Gender + Age + GPA, data = data_HAMNat_Incumbents)
## 
## Standardized Coefficients::
## (Intercept)      Gender         Age         GPA 
##          NA  -0.1319463   0.1166899   0.2300913
```

```
lm.beta(lm_HAMNat_M1_2b)
```

```
## 
## Call:
## lm(formula = M1 ~ Gender + Age + HAMNat_total, data = data_HAMNat_Incumbents)
## 
## Standardized Coefficients::
##  (Intercept)       Gender          Age HAMNat_total 
##           NA  -0.02330468   0.19661701  -0.43945205
```

```
lm.beta(lm_HAMNat_M1_3)
```

```
## 
## Call:
## lm(formula = M1 ~ Gender + Age + GPA + HAMNat_total, data = data_HAMNat_Incumbents)
## 
## Standardized Coefficients::
##  (Intercept)       Gender          Age          GPA HAMNat_total 
##           NA  -0.03631674   0.09197143   0.18619714  -0.42589705
```

```
anova(lm_HAMNat_M1_1, lm_HAMNat_M1_2a, lm_HAMNat_M1_3)
```

```
## Analysis of Variance Table
## 
## Model 1: M1 ~ Gender + Age
## Model 2: M1 ~ Gender + Age + GPA
## Model 3: M1 ~ Gender + Age + GPA + HAMNat_total
##   Res.Df    RSS Df Sum of Sq      F    Pr(>F)    
## 1    230 153.69                                  
## 2    229 147.86  1    5.8304 11.097  0.001008 ** 
## 3    228 119.79  1   28.0680 53.423 4.499e-12 ***
## ---
## Signif. codes:  0 '***' 0.001 '**' 0.01 '*' 0.05 '.' 0.1 ' ' 1
```

```
anova(lm_HAMNat_M1_1, lm_HAMNat_M1_2b, lm_HAMNat_M1_3)
```

```
## Analysis of Variance Table
## 
## Model 1: M1 ~ Gender + Age
## Model 2: M1 ~ Gender + Age + HAMNat_total
## Model 3: M1 ~ Gender + Age + GPA + HAMNat_total
##   Res.Df    RSS Df Sum of Sq       F    Pr(>F)    
## 1    230 153.69                                   
## 2    229 123.58  1   30.1090 57.3081 9.175e-13 ***
## 3    228 119.79  1    3.7894  7.2126  0.007772 ** 
## ---
## Signif. codes:  0 '***' 0.001 '**' 0.01 '*' 0.05 '.' 0.1 ' ' 1
```

## 3.2 Regression analyses with correction for effects of range restriction by the correction formula of Aitken and Lawley

### 3.2.1 TMS

```
#Criterion: PCGPA

#Correcting for range restriction
data_TMS_Incumbents$Gender <- as.numeric(data_TMS_Incumbents$Gender)
Cov_Mat_TMS_Incumbents <- cov((dplyr::select(data_TMS_Incumbents, Gender, Age, GPA, TMS_total, PCGPA)), use = "complete.obs")
Cor_Mat_TMS_Incumbents <- cor((dplyr::select(data_TMS_Incumbents, Gender, Age, GPA, TMS_total, PCGPA)), use = "complete.obs")
Cov_Mat_TMS_Testtakers <- cov((dplyr::select(data_TMS_Testtakers, Gender, Age, GPA, TMS_total)))
Cor_Mat_TMS_Testtakers <- cor((dplyr::select(data_TMS_Testtakers, Gender, Age, GPA, TMS_total)))
Cor_Mat_TMS_Incumbents_Corr <- lMvrrc(rcov = Cov_Mat_TMS_Incumbents, vnp = Cov_Mat_TMS_Testtakers, as_cor = T)
rownames(Cor_Mat_TMS_Incumbents_Corr) <- colnames(Cor_Mat_TMS_Incumbents_Corr) <- c('Gender', 'Age', 'GPA', 'TMS_total', 'PCGPA')

#Models
lm_TMS_1_corr <- lmCor(PCGPA ~ Gender + Age, data = Cor_Mat_TMS_Incumbents_Corr, n.obs = nobs(lm_TMS_1), plot = F)
lm_TMS_2a_corr <- lmCor(PCGPA ~ Gender + Age + GPA, data = Cor_Mat_TMS_Incumbents_Corr, n.obs = nobs(lm_TMS_2a), plot = F)
lm_TMS_2b_corr <- lmCor(PCGPA ~ Gender + Age + TMS_total, data = Cor_Mat_TMS_Incumbents_Corr, n.obs = nobs(lm_TMS_2b), plot = F)
lm_TMS_3_corr <- lmCor(PCGPA ~ Gender + Age + GPA + TMS_total, data = Cor_Mat_TMS_Incumbents_Corr, n.obs = nobs(lm_TMS_3), plot = F)

#Results
lm_TMS_1_corr
```

```
## Call: lmCor(y = PCGPA ~ Gender + Age, data = Cor_Mat_TMS_Incumbents_Corr, 
##     n.obs = nobs(lm_TMS_1), plot = F)
## 
## Multiple Regression from matrix input 
## 
##  DV =  PCGPA 
##        slope   se     t       p lower.ci upper.ci VIF Vy.x
## Gender -0.11 0.02 -4.63 4.0e-06    -0.15    -0.06   1 0.01
## Age     0.11 0.02  4.94 8.4e-07     0.07     0.16   1 0.01
## 
## Residual Standard Error =  0.99  with  1857  degrees of freedom
## 
##  Multiple Regression
##          R   R2  Ruw R2uw Shrunken R2 SE of R2 overall F df1  df2        p
## PCGPA 0.15 0.02 0.15 0.02        0.02     0.01     21.79   2 1857 4.43e-10
```

```
lm_TMS_2a_corr
```

```
## Call: lmCor(y = PCGPA ~ Gender + Age + GPA, data = Cor_Mat_TMS_Incumbents_Corr, 
##     n.obs = nobs(lm_TMS_2a), plot = F)
## 
## Multiple Regression from matrix input 
## 
##  DV =  PCGPA 
##        slope   se     t       p lower.ci upper.ci  VIF Vy.x
## Gender -0.11 0.02 -4.76 2.1e-06    -0.15    -0.06 1.00 0.01
## Age     0.03 0.03  1.32 1.9e-01    -0.02     0.08 1.27 0.00
## GPA     0.17 0.03  6.79 1.5e-11     0.12     0.22 1.27 0.03
## 
## Residual Standard Error =  0.98  with  1856  degrees of freedom
## 
##  Multiple Regression
##          R   R2 Ruw R2uw Shrunken R2 SE of R2 overall F df1  df2        p
## PCGPA 0.22 0.05 0.2 0.04        0.05     0.01     30.25   3 1856 4.31e-19
```

```
lm_TMS_2b_corr
```

```
## Call: lmCor(y = PCGPA ~ Gender + Age + TMS_total, data = Cor_Mat_TMS_Incumbents_Corr, 
##     n.obs = nobs(lm_TMS_2b), plot = F)
## 
## Multiple Regression from matrix input 
## 
##  DV =  PCGPA 
##           slope   se     t       p lower.ci upper.ci  VIF Vy.x
## Gender    -0.09 0.02 -3.83 1.3e-04    -0.13    -0.04 1.01 0.01
## Age        0.08 0.02  3.37 7.7e-04     0.03     0.12 1.04 0.01
## TMS_total -0.21 0.02 -9.00 5.4e-19    -0.25    -0.16 1.04 0.05
## 
## Residual Standard Error =  0.97  with  1856  degrees of freedom
## 
##  Multiple Regression
##          R   R2  Ruw R2uw Shrunken R2 SE of R2 overall F df1  df2        p
## PCGPA 0.25 0.06 0.24 0.06        0.06     0.01     42.15   3 1856 2.39e-26
```

```
lm_TMS_3_corr
```

```
## Call: lmCor(y = PCGPA ~ Gender + Age + GPA + TMS_total, data = Cor_Mat_TMS_Incumbents_Corr, 
##     n.obs = nobs(lm_TMS_3), plot = F)
## 
## Multiple Regression from matrix input 
## 
##  DV =  PCGPA 
##           slope   se     t       p lower.ci upper.ci  VIF Vy.x
## Gender    -0.09 0.02 -4.02 6.0e-05    -0.13    -0.05 1.01 0.01
## Age        0.02 0.03  0.90 3.7e-01    -0.03     0.07 1.28 0.00
## GPA        0.13 0.03  4.98 7.0e-07     0.08     0.18 1.34 0.02
## TMS_total -0.18 0.02 -7.70 2.2e-14    -0.23    -0.13 1.10 0.04
## 
## Residual Standard Error =  0.96  with  1855  degrees of freedom
## 
##  Multiple Regression
##          R   R2  Ruw R2uw Shrunken R2 SE of R2 overall F df1  df2        p
## PCGPA 0.28 0.08 0.26 0.07        0.07     0.01     38.21   4 1855 9.12e-31
```

```
anova(lm_TMS_1_corr, lm_TMS_2a_corr, lm_TMS_3_corr)
```

```
## Model 1 = lmCor(y = PCGPA ~ Gender + Age, data = Cor_Mat_TMS_Incumbents_Corr, 
##     n.obs = nobs(lm_TMS_1), plot = F)
## Model 2 = lmCor(y = PCGPA ~ Gender + Age + GPA, data = Cor_Mat_TMS_Incumbents_Corr, 
##     n.obs = nobs(lm_TMS_2a), plot = F)
## Model 3 = lmCor(y = PCGPA ~ Gender + Age + GPA + TMS_total, data = Cor_Mat_TMS_Incumbents_Corr, 
##     n.obs = nobs(lm_TMS_3), plot = F)
```

```
## $PCGPA
##   Res Df   Res SS Diff df  Diff SS        F     Pr(F > )
## 1   1857 1816.373      NA       NA       NA           NA
## 2   1856 1772.336       1 44.03699 47.56314 7.277322e-12
## 3   1855 1717.477       1 54.85830 59.25094 2.240550e-14
```

```
anova(lm_TMS_1_corr, lm_TMS_2b_corr, lm_TMS_3_corr)
```

```
## Model 1 = lmCor(y = PCGPA ~ Gender + Age, data = Cor_Mat_TMS_Incumbents_Corr, 
##     n.obs = nobs(lm_TMS_1), plot = F)
## Model 2 = lmCor(y = PCGPA ~ Gender + Age + TMS_total, data = Cor_Mat_TMS_Incumbents_Corr, 
##     n.obs = nobs(lm_TMS_2b), plot = F)
## Model 3 = lmCor(y = PCGPA ~ Gender + Age + GPA + TMS_total, data = Cor_Mat_TMS_Incumbents_Corr, 
##     n.obs = nobs(lm_TMS_3), plot = F)
```

```
## $PCGPA
##   Res Df   Res SS Diff df  Diff SS        F     Pr(F > )
## 1   1857 1816.373      NA       NA       NA           NA
## 2   1856 1740.420       1 75.95317 82.03492 3.295580e-19
## 3   1855 1717.477       1 22.94212 24.77915 7.024183e-07
```

```
#Criterion: M1

#Correcting for range restriction
Cov_Mat_TMS_Incumbents_M1 <- cov((dplyr::select(data_TMS_Incumbents, Gender, Age, GPA, TMS_total, M1)), use = "complete.obs")
Cor_Mat_TMS_Incumbents_M1 <- cor((dplyr::select(data_TMS_Incumbents, Gender, Age, GPA, TMS_total, M1)), use = "complete.obs")
Cor_Mat_TMS_Incumbents_Corr_M1 <- lMvrrc(rcov = Cov_Mat_TMS_Incumbents_M1, vnp = Cov_Mat_TMS_Testtakers, as_cor = T)
rownames(Cor_Mat_TMS_Incumbents_Corr_M1) <- colnames(Cor_Mat_TMS_Incumbents_Corr_M1) <- c('Gender', 'Age', 'GPA', 'TMS_total', 'M1')

#Models
lm_TMS_M1_1_corr <- lmCor(M1 ~ Gender + Age, data = Cor_Mat_TMS_Incumbents_Corr_M1, n.obs = nobs(lm_TMS_M1_1), plot = F)
lm_TMS_M1_2a_corr <- lmCor(M1 ~ Gender + Age + GPA, data = Cor_Mat_TMS_Incumbents_Corr_M1, n.obs = nobs(lm_TMS_M1_2a), plot = F)
lm_TMS_M1_2b_corr <- lmCor(M1 ~ Gender + Age + TMS_total, data = Cor_Mat_TMS_Incumbents_Corr_M1, n.obs = nobs(lm_TMS_M1_2b), plot = F)
lm_TMS_M1_3_corr <- lmCor(M1 ~ Gender + Age + GPA + TMS_total, data = Cor_Mat_TMS_Incumbents_Corr_M1, n.obs = nobs(lm_TMS_M1_3), plot = F)

#Results
lm_TMS_M1_1_corr
```

```
## Call: lmCor(y = M1 ~ Gender + Age, data = Cor_Mat_TMS_Incumbents_Corr_M1, 
##     n.obs = nobs(lm_TMS_M1_1), plot = F)
## 
## Multiple Regression from matrix input 
## 
##  DV =  M1 
##        slope   se     t       p lower.ci upper.ci VIF Vy.x
## Gender -0.16 0.05 -3.15 0.00180    -0.26    -0.06   1 0.02
## Age     0.18 0.05  3.52 0.00049     0.08     0.28   1 0.03
## 
## Residual Standard Error =  0.98  with  372  degrees of freedom
## 
##  Multiple Regression
##       R   R2  Ruw R2uw Shrunken R2 SE of R2 overall F df1 df2        p
## M1 0.23 0.05 0.23 0.05        0.05     0.02     10.59   2 372 3.35e-05
```

```
lm_TMS_M1_2a_corr
```

```
## Call: lmCor(y = M1 ~ Gender + Age + GPA, data = Cor_Mat_TMS_Incumbents_Corr_M1, 
##     n.obs = nobs(lm_TMS_M1_2a), plot = F)
## 
## Multiple Regression from matrix input 
## 
##  DV =  M1 
##        slope   se     t       p lower.ci upper.ci  VIF Vy.x
## Gender -0.16 0.05 -3.27 1.2e-03    -0.26    -0.06 1.00 0.02
## Age     0.07 0.06  1.22 2.2e-01    -0.04     0.18 1.27 0.01
## GPA     0.24 0.06  4.30 2.2e-05     0.13     0.35 1.27 0.06
## 
## Residual Standard Error =  0.95  with  371  degrees of freedom
## 
##  Multiple Regression
##       R  R2 Ruw R2uw Shrunken R2 SE of R2 overall F df1 df2        p
## M1 0.31 0.1 0.3 0.09        0.09     0.03     13.56   3 371 2.05e-08
```

```
lm_TMS_M1_2b_corr
```

```
## Call: lmCor(y = M1 ~ Gender + Age + TMS_total, data = Cor_Mat_TMS_Incumbents_Corr_M1, 
##     n.obs = nobs(lm_TMS_M1_2b), plot = F)
## 
## Multiple Regression from matrix input 
## 
##  DV =  M1 
##           slope   se     t       p lower.ci upper.ci  VIF Vy.x
## Gender    -0.14 0.05 -2.78 5.7e-03    -0.23    -0.04 1.01 0.02
## Age        0.14 0.05  2.75 6.3e-03     0.04     0.24 1.04 0.02
## TMS_total -0.23 0.05 -4.50 9.3e-06    -0.32    -0.13 1.04 0.06
## 
## Residual Standard Error =  0.95  with  371  degrees of freedom
## 
##  Multiple Regression
##       R  R2  Ruw R2uw Shrunken R2 SE of R2 overall F df1 df2        p
## M1 0.32 0.1 0.31  0.1         0.1     0.03     14.16   3 371 9.28e-09
```

```
lm_TMS_M1_3_corr
```

```
## Call: lmCor(y = M1 ~ Gender + Age + GPA + TMS_total, data = Cor_Mat_TMS_Incumbents_Corr_M1, 
##     n.obs = nobs(lm_TMS_M1_3), plot = F)
## 
## Multiple Regression from matrix input 
## 
##  DV =  M1 
##           slope   se     t       p lower.ci upper.ci  VIF Vy.x
## Gender    -0.14 0.05 -2.93 0.00360    -0.24    -0.05 1.01 0.02
## Age        0.06 0.05  1.02 0.31000    -0.05     0.16 1.28 0.01
## GPA        0.19 0.06  3.43 0.00067     0.08     0.30 1.34 0.05
## TMS_total -0.19 0.05 -3.67 0.00028    -0.29    -0.09 1.10 0.05
## 
## Residual Standard Error =  0.94  with  370  degrees of freedom
## 
##  Multiple Regression
##       R   R2  Ruw R2uw Shrunken R2 SE of R2 overall F df1 df2        p
## M1 0.36 0.13 0.35 0.12        0.12     0.03     13.87   4 370 1.49e-10
```

```
anova(lm_TMS_M1_1_corr, lm_TMS_M1_2a_corr, lm_TMS_M1_3_corr)
```

```
## Model 1 = lmCor(y = M1 ~ Gender + Age, data = Cor_Mat_TMS_Incumbents_Corr_M1, 
##     n.obs = nobs(lm_TMS_M1_1), plot = F)
## Model 2 = lmCor(y = M1 ~ Gender + Age + GPA, data = Cor_Mat_TMS_Incumbents_Corr_M1, 
##     n.obs = nobs(lm_TMS_M1_2a), plot = F)
## Model 3 = lmCor(y = M1 ~ Gender + Age + GPA + TMS_total, data = Cor_Mat_TMS_Incumbents_Corr_M1, 
##     n.obs = nobs(lm_TMS_M1_3), plot = F)
```

```
## $M1
##   Res Df   Res SS Diff df  Diff SS        F     Pr(F > )
## 1    372 353.8443      NA       NA       NA           NA
## 2    371 337.0371       1 16.80716 19.12129 1.595519e-05
## 3    370 325.2212       1 11.81595 13.44286 2.819525e-04
```

```
anova(lm_TMS_M1_1_corr, lm_TMS_M1_2b_corr, lm_TMS_M1_3_corr)
```

```
## Model 1 = lmCor(y = M1 ~ Gender + Age, data = Cor_Mat_TMS_Incumbents_Corr_M1, 
##     n.obs = nobs(lm_TMS_M1_1), plot = F)
## Model 2 = lmCor(y = M1 ~ Gender + Age + TMS_total, data = Cor_Mat_TMS_Incumbents_Corr_M1, 
##     n.obs = nobs(lm_TMS_M1_2b), plot = F)
## Model 3 = lmCor(y = M1 ~ Gender + Age + GPA + TMS_total, data = Cor_Mat_TMS_Incumbents_Corr_M1, 
##     n.obs = nobs(lm_TMS_M1_3), plot = F)
```

```
## $M1
##   Res Df   Res SS Diff df  Diff SS        F     Pr(F > )
## 1    372 353.8443      NA       NA       NA           NA
## 2    371 335.5664       1 18.27785 20.79448 6.955298e-06
## 3    370 325.2212       1 10.34526 11.76967 6.699751e-04
```

### 3.2.2 HAM-Nat

```
#Criterion: PCGPA

#Correcting for range restriction
Cov_Mat_HAMNat_Incumbents <- cov((dplyr::select(data_HAMNat_Incumbents, Gender, Age, GPA, HAMNat_total, PCGPA)), use = "complete.obs")
Cor_Mat_HAMNat_Incumbents <- cor((dplyr::select(data_HAMNat_Incumbents, Gender, Age, GPA, HAMNat_total, PCGPA)), use = "complete.obs")
Cov_Mat_HAMNat_Testtakers <- cov((dplyr::select(data_HAMNat_Testtakers, Gender, Age, GPA, HAMNat_total)))
Cor_Mat_HAMNat_Testtakers <- cor((dplyr::select(data_HAMNat_Testtakers, Gender, Age, GPA, HAMNat_total)))
Cor_Mat_HAMNat_Incumbents_Corr <- lMvrrc(rcov = Cov_Mat_HAMNat_Incumbents, vnp = Cov_Mat_HAMNat_Testtakers, as_cor = T)
rownames(Cor_Mat_HAMNat_Incumbents_Corr) <- colnames(Cor_Mat_HAMNat_Incumbents_Corr) <- c('Gender', 'Age', 'GPA', 'HAMNat_total', 'PCGPA')

#Models
lm_HAMNat_1_corr <- lmCor(PCGPA ~ Gender + Age, data = Cor_Mat_HAMNat_Incumbents_Corr, n.obs = nobs(lm_HAMNat_1), plot = F)
lm_HAMNat_2a_corr <- lmCor(PCGPA ~ Gender + Age + GPA, data = Cor_Mat_HAMNat_Incumbents_Corr, n.obs = nobs(lm_HAMNat_2a), plot = F)
lm_HAMNat_2b_corr <- lmCor(PCGPA ~ Gender + Age + HAMNat_total, data = Cor_Mat_HAMNat_Incumbents_Corr, n.obs = nobs(lm_HAMNat_2b), plot = F)
lm_HAMNat_3_corr <- lmCor(PCGPA ~ Gender + Age + GPA + HAMNat_total, data = Cor_Mat_HAMNat_Incumbents_Corr, n.obs = nobs(lm_HAMNat_3), plot = F)

#Results
lm_HAMNat_1_corr
```

```
## Call: lmCor(y = PCGPA ~ Gender + Age, data = Cor_Mat_HAMNat_Incumbents_Corr, 
##     n.obs = nobs(lm_HAMNat_1), plot = F)
## 
## Multiple Regression from matrix input 
## 
##  DV =  PCGPA 
##        slope   se     t    p lower.ci upper.ci VIF Vy.x
## Gender -0.04 0.04 -0.96 0.34    -0.11     0.04   1    0
## Age     0.05 0.04  1.36 0.17    -0.02     0.13   1    0
## 
## Residual Standard Error =  1  with  696  degrees of freedom
## 
##  Multiple Regression
##          R R2  Ruw R2uw Shrunken R2 SE of R2 overall F df1 df2     p
## PCGPA 0.06  0 0.06    0           0        0      1.36   2 696 0.258
```

```
lm_HAMNat_2a_corr
```

```
## Call: lmCor(y = PCGPA ~ Gender + Age + GPA, data = Cor_Mat_HAMNat_Incumbents_Corr, 
##     n.obs = nobs(lm_HAMNat_2a), plot = F)
## 
## Multiple Regression from matrix input 
## 
##  DV =  PCGPA 
##        slope   se     t       p lower.ci upper.ci VIF Vy.x
## Gender -0.04 0.04 -1.10 2.7e-01    -0.11     0.03 1.0 0.00
## Age    -0.05 0.04 -1.16 2.5e-01    -0.13     0.03 1.2 0.00
## GPA     0.24 0.04  5.98 3.6e-09     0.16     0.32 1.2 0.05
## 
## Residual Standard Error =  0.98  with  695  degrees of freedom
## 
##  Multiple Regression
##          R   R2  Ruw R2uw Shrunken R2 SE of R2 overall F df1 df2        p
## PCGPA 0.23 0.05 0.16 0.03        0.05     0.02     12.86   3 695 3.49e-08
```

```
lm_HAMNat_2b_corr
```

```
## Call: lmCor(y = PCGPA ~ Gender + Age + HAMNat_total, data = Cor_Mat_HAMNat_Incumbents_Corr, 
##     n.obs = nobs(lm_HAMNat_2b), plot = F)
## 
## Multiple Regression from matrix input 
## 
##  DV =  PCGPA 
##              slope   se     t       p lower.ci upper.ci  VIF Vy.x
## Gender        0.04 0.04  1.01 3.1e-01    -0.03     0.11 1.04 0.00
## Age           0.03 0.04  0.90 3.7e-01    -0.04     0.10 1.00 0.00
## HAMNat_total -0.36 0.04 -9.98 5.2e-22    -0.43    -0.29 1.04 0.13
## 
## Residual Standard Error =  0.94  with  695  degrees of freedom
## 
##  Multiple Regression
##          R   R2  Ruw R2uw Shrunken R2 SE of R2 overall F df1 df2        p
## PCGPA 0.36 0.13 0.24 0.06        0.12     0.02     34.23   3 695 1.21e-20
```

```
lm_HAMNat_3_corr
```

```
## Call: lmCor(y = PCGPA ~ Gender + Age + GPA + HAMNat_total, data = Cor_Mat_HAMNat_Incumbents_Corr, 
##     n.obs = nobs(lm_HAMNat_3), plot = F)
## 
## Multiple Regression from matrix input 
## 
##  DV =  PCGPA 
##              slope   se     t       p lower.ci upper.ci  VIF Vy.x
## Gender        0.03 0.04  0.73 4.7e-01    -0.04     0.10 1.05 0.00
## Age          -0.02 0.04 -0.58 5.6e-01    -0.10     0.05 1.21 0.00
## GPA           0.14 0.04  3.44 6.2e-04     0.06     0.22 1.32 0.03
## HAMNat_total -0.32 0.04 -8.57 6.5e-17    -0.40    -0.25 1.15 0.11
## 
## Residual Standard Error =  0.93  with  694  degrees of freedom
## 
##  Multiple Regression
##          R   R2  Ruw R2uw Shrunken R2 SE of R2 overall F df1 df2        p
## PCGPA 0.38 0.14 0.28 0.08        0.14     0.02     29.03   4 694 2.47e-22
```

```
anova(lm_HAMNat_1_corr, lm_HAMNat_2a_corr, lm_HAMNat_3_corr)
```

```
## Model 1 = lmCor(y = PCGPA ~ Gender + Age, data = Cor_Mat_HAMNat_Incumbents_Corr, 
##     n.obs = nobs(lm_HAMNat_1), plot = F)
## Model 2 = lmCor(y = PCGPA ~ Gender + Age + GPA, data = Cor_Mat_HAMNat_Incumbents_Corr, 
##     n.obs = nobs(lm_HAMNat_2a), plot = F)
## Model 3 = lmCor(y = PCGPA ~ Gender + Age + GPA + HAMNat_total, data = Cor_Mat_HAMNat_Incumbents_Corr, 
##     n.obs = nobs(lm_HAMNat_3), plot = F)
```

```
## $PCGPA
##   Res Df   Res SS Diff df  Diff SS        F     Pr(F > )
## 1    696 695.2853      NA       NA       NA           NA
## 2    695 661.2959       1 33.98944 39.44848 5.936816e-10
## 3    694 597.9616       1 63.33432 73.50642 6.502097e-17
```

```
anova(lm_HAMNat_1_corr, lm_HAMNat_2b_corr, lm_HAMNat_3_corr)
```

```
## Model 1 = lmCor(y = PCGPA ~ Gender + Age, data = Cor_Mat_HAMNat_Incumbents_Corr, 
##     n.obs = nobs(lm_HAMNat_1), plot = F)
## Model 2 = lmCor(y = PCGPA ~ Gender + Age + HAMNat_total, data = Cor_Mat_HAMNat_Incumbents_Corr, 
##     n.obs = nobs(lm_HAMNat_2b), plot = F)
## Model 3 = lmCor(y = PCGPA ~ Gender + Age + GPA + HAMNat_total, data = Cor_Mat_HAMNat_Incumbents_Corr, 
##     n.obs = nobs(lm_HAMNat_3), plot = F)
```

```
## $PCGPA
##   Res Df   Res SS Diff df  Diff SS         F     Pr(F > )
## 1    696 695.2853      NA       NA        NA           NA
## 2    695 608.1420       1 87.14333 101.13939 2.634196e-22
## 3    694 597.9616       1 10.18044  11.81551 6.224426e-04
```

```
#Criterion: M1

#Correcting for range restriction
Cov_Mat_HAMNat_Incumbents_M1 <- cov((dplyr::select(data_HAMNat_Incumbents, Gender, Age, GPA, HAMNat_total, M1)), use = "complete.obs")
Cor_Mat_HAMNat_Incumbents_M1 <- cor((dplyr::select(data_HAMNat_Incumbents, Gender, Age, GPA, HAMNat_total, M1)), use = "complete.obs")
Cor_Mat_HAMNat_Incumbents_Corr_M1 <- lMvrrc(rcov = Cov_Mat_HAMNat_Incumbents_M1, vnp = Cov_Mat_HAMNat_Testtakers, as_cor = T)
rownames(Cor_Mat_HAMNat_Incumbents_Corr_M1) <- colnames(Cor_Mat_HAMNat_Incumbents_Corr_M1) <- c('Gender', 'Age', 'GPA', 'HAMNat_total', 'M1')

#Models
lm_HAMNat_1_corr_M1 <- lmCor(M1 ~ Gender + Age, data = Cor_Mat_HAMNat_Incumbents_Corr_M1, n.obs = nobs(lm_HAMNat_M1_1), plot = F)
lm_HAMNat_2a_corr_M1 <- lmCor(M1 ~ Gender + Age + GPA, data = Cor_Mat_HAMNat_Incumbents_Corr_M1, n.obs = nobs(lm_HAMNat_M1_2a), plot = F)
lm_HAMNat_2b_corr_M1 <- lmCor(M1 ~ Gender + Age + HAMNat_total, data = Cor_Mat_HAMNat_Incumbents_Corr_M1, n.obs = nobs(lm_HAMNat_M1_2b), plot = F)
lm_HAMNat_3_corr_M1 <- lmCor(M1 ~ Gender + Age + GPA + HAMNat_total, data = Cor_Mat_HAMNat_Incumbents_Corr_M1, n.obs = nobs(lm_HAMNat_M1_3), plot = F)

#Results
lm_HAMNat_1_corr_M1
```

```
## Call: lmCor(y = M1 ~ Gender + Age, data = Cor_Mat_HAMNat_Incumbents_Corr_M1, 
##     n.obs = nobs(lm_HAMNat_M1_1), plot = F)
## 
## Multiple Regression from matrix input 
## 
##  DV =  M1 
##        slope   se     t      p lower.ci upper.ci VIF Vy.x
## Gender -0.11 0.06 -1.77 0.0780    -0.24     0.01   1 0.01
## Age     0.19 0.06  3.03 0.0027     0.07     0.32   1 0.04
## 
## Residual Standard Error =  0.98  with  230  degrees of freedom
## 
##  Multiple Regression
##       R   R2  Ruw R2uw Shrunken R2 SE of R2 overall F df1 df2       p
## M1 0.22 0.05 0.22 0.05        0.04     0.03      6.06   2 230 0.00273
```

```
lm_HAMNat_2a_corr_M1
```

```
## Call: lmCor(y = M1 ~ Gender + Age + GPA, data = Cor_Mat_HAMNat_Incumbents_Corr_M1, 
##     n.obs = nobs(lm_HAMNat_M1_2a), plot = F)
## 
## Multiple Regression from matrix input 
## 
##  DV =  M1 
##        slope   se     t       p lower.ci upper.ci VIF Vy.x
## Gender -0.12 0.06 -1.96 5.2e-02    -0.24     0.00 1.0 0.01
## Age     0.06 0.07  0.88 3.8e-01    -0.07     0.19 1.2 0.01
## GPA     0.33 0.07  4.97 1.3e-06     0.20     0.47 1.2 0.12
## 
## Residual Standard Error =  0.93  with  229  degrees of freedom
## 
##  Multiple Regression
##       R   R2  Ruw R2uw Shrunken R2 SE of R2 overall F df1 df2        p
## M1 0.38 0.14 0.34 0.12        0.13     0.04      12.7   3 229 1.04e-07
```

```
lm_HAMNat_2b_corr_M1
```

```
## Call: lmCor(y = M1 ~ Gender + Age + HAMNat_total, data = Cor_Mat_HAMNat_Incumbents_Corr_M1, 
##     n.obs = nobs(lm_HAMNat_M1_2b), plot = F)
## 
## Multiple Regression from matrix input 
## 
##  DV =  M1 
##              slope   se     t       p lower.ci upper.ci  VIF Vy.x
## Gender       -0.02 0.06 -0.33 7.4e-01    -0.13     0.10 1.04 0.00
## Age           0.17 0.06  2.98 3.2e-03     0.06     0.28 1.00 0.03
## HAMNat_total -0.47 0.06 -8.12 2.9e-14    -0.59    -0.36 1.04 0.23
## 
## Residual Standard Error =  0.86  with  229  degrees of freedom
## 
##  Multiple Regression
##       R   R2  Ruw R2uw Shrunken R2 SE of R2 overall F df1 df2        p
## M1 0.51 0.26 0.42 0.18        0.25     0.05     27.16   3 229 4.59e-15
```

```
lm_HAMNat_3_corr_M1
```

```
## Call: lmCor(y = M1 ~ Gender + Age + GPA + HAMNat_total, data = Cor_Mat_HAMNat_Incumbents_Corr_M1, 
##     n.obs = nobs(lm_HAMNat_M1_3), plot = F)
## 
## Multiple Regression from matrix input 
## 
##  DV =  M1 
##              slope   se     t       p lower.ci upper.ci  VIF Vy.x
## Gender       -0.03 0.06 -0.59 5.5e-01    -0.15     0.08 1.05 0.00
## Age           0.09 0.06  1.48 1.4e-01    -0.03     0.21 1.21 0.02
## GPA           0.20 0.06  3.14 1.9e-03     0.07     0.33 1.32 0.07
## HAMNat_total -0.42 0.06 -6.96 3.5e-11    -0.53    -0.30 1.15 0.20
## 
## Residual Standard Error =  0.85  with  228  degrees of freedom
## 
##  Multiple Regression
##       R   R2  Ruw R2uw Shrunken R2 SE of R2 overall F df1 df2        p
## M1 0.54 0.29 0.47 0.22        0.28     0.05     23.62   4 228 2.35e-16
```

```
anova(lm_HAMNat_1_corr_M1, lm_HAMNat_2a_corr_M1, lm_HAMNat_3_corr_M1)
```

```
## Model 1 = lmCor(y = M1 ~ Gender + Age, data = Cor_Mat_HAMNat_Incumbents_Corr_M1, 
##     n.obs = nobs(lm_HAMNat_M1_1), plot = F)
## Model 2 = lmCor(y = M1 ~ Gender + Age + GPA, data = Cor_Mat_HAMNat_Incumbents_Corr_M1, 
##     n.obs = nobs(lm_HAMNat_M1_2a), plot = F)
## Model 3 = lmCor(y = M1 ~ Gender + Age + GPA + HAMNat_total, data = Cor_Mat_HAMNat_Incumbents_Corr_M1, 
##     n.obs = nobs(lm_HAMNat_M1_3), plot = F)
```

```
## $M1
##   Res Df   Res SS Diff df  Diff SS        F     Pr(F > )
## 1    230 220.3920      NA       NA       NA           NA
## 2    229 198.9016       1 21.49035 29.87233 1.202067e-07
## 3    228 164.0247       1 34.87697 48.48020 3.523297e-11
```

```
anova(lm_HAMNat_1_corr_M1, lm_HAMNat_2b_corr_M1, lm_HAMNat_3_corr_M1)
```

```
## Model 1 = lmCor(y = M1 ~ Gender + Age, data = Cor_Mat_HAMNat_Incumbents_Corr_M1, 
##     n.obs = nobs(lm_HAMNat_M1_1), plot = F)
## Model 2 = lmCor(y = M1 ~ Gender + Age + HAMNat_total, data = Cor_Mat_HAMNat_Incumbents_Corr_M1, 
##     n.obs = nobs(lm_HAMNat_M1_2b), plot = F)
## Model 3 = lmCor(y = M1 ~ Gender + Age + GPA + HAMNat_total, data = Cor_Mat_HAMNat_Incumbents_Corr_M1, 
##     n.obs = nobs(lm_HAMNat_M1_3), plot = F)
```

```
## $M1
##   Res Df   Res SS Diff df   Diff SS         F     Pr(F > )
## 1    230 220.3920      NA        NA        NA           NA
## 2    229 171.1166       1 49.275406 68.494527 1.052457e-14
## 3    228 164.0247       1  7.091912  9.858004 1.914482e-03
```

## 3.3 Regression analyses split by univerisities with selection via TMS/ HAM-Nat

### 3.3.1 Regression analyses in universities with selection via TMS (uncorrected)

#### 3.3.1.1 TMS

```
#Criterion: PCGPA

#Models
lm_TMS_via_Uni_TMS_1 <- lm(PCGPA ~ Gender + Age, data = data_TMS_via_Uni_TMS)
lm_TMS_via_Uni_TMS_2a <- lm(PCGPA ~ Gender + Age + GPA, data = data_TMS_via_Uni_TMS)
lm_TMS_via_Uni_TMS_2b <- lm(PCGPA ~ Gender + Age + TMS_total, data = data_TMS_via_Uni_TMS)
lm_TMS_via_Uni_TMS_3 <- lm(PCGPA ~ Gender + Age + GPA + TMS_total, data = data_TMS_via_Uni_TMS)

#Results
summary(lm_TMS_via_Uni_TMS_1)
```

```
## 
## Call:
## lm(formula = PCGPA ~ Gender + Age, data = data_TMS_via_Uni_TMS)
## 
## Residuals:
##      Min       1Q   Median       3Q      Max 
## -1.73839 -0.44850 -0.08726  0.42088  1.84336 
## 
## Coefficients:
##              Estimate Std. Error t value Pr(>|t|)    
## (Intercept)  1.762038   0.130069  13.547  < 2e-16 ***
## Gender      -0.156523   0.034104  -4.590 4.77e-06 ***
## Age          0.030618   0.006086   5.031 5.42e-07 ***
## ---
## Signif. codes:  0 '***' 0.001 '**' 0.01 '*' 0.05 '.' 0.1 ' ' 1
## 
## Residual standard error: 0.6366 on 1670 degrees of freedom
##   (12 Beobachtungen als fehlend gelöscht)
## Multiple R-squared:  0.02529,    Adjusted R-squared:  0.02413 
## F-statistic: 21.67 on 2 and 1670 DF,  p-value: 5.122e-10
```

```
summary(lm_TMS_via_Uni_TMS_2a)
```

```
## 
## Call:
## lm(formula = PCGPA ~ Gender + Age + GPA, data = data_TMS_via_Uni_TMS)
## 
## Residuals:
##      Min       1Q   Median       3Q      Max 
## -1.73009 -0.46048 -0.09778  0.42324  1.82665 
## 
## Coefficients:
##              Estimate Std. Error t value Pr(>|t|)    
## (Intercept)  1.885646   0.132996  14.178  < 2e-16 ***
## Gender      -0.166507   0.034035  -4.892 1.09e-06 ***
## Age          0.012432   0.007532   1.650    0.099 .  
## GPA          0.172177   0.042375   4.063 5.07e-05 ***
## ---
## Signif. codes:  0 '***' 0.001 '**' 0.01 '*' 0.05 '.' 0.1 ' ' 1
## 
## Residual standard error: 0.6337 on 1669 degrees of freedom
##   (12 Beobachtungen als fehlend gelöscht)
## Multiple R-squared:  0.03484,    Adjusted R-squared:  0.03311 
## F-statistic: 20.08 on 3 and 1669 DF,  p-value: 8.692e-13
```

```
summary(lm_TMS_via_Uni_TMS_2b)
```

```
## 
## Call:
## lm(formula = PCGPA ~ Gender + Age + TMS_total, data = data_TMS_via_Uni_TMS)
## 
## Residuals:
##      Min       1Q   Median       3Q      Max 
## -1.54058 -0.43393 -0.09008  0.40992  1.89563 
## 
## Coefficients:
##              Estimate Std. Error t value Pr(>|t|)    
## (Intercept)  3.205643   0.267509  11.983  < 2e-16 ***
## Gender      -0.137689   0.033872  -4.065 5.03e-05 ***
## Age          0.024263   0.006108   3.972 7.42e-05 ***
## TMS_total   -0.012392   0.002013  -6.155 9.38e-10 ***
## ---
## Signif. codes:  0 '***' 0.001 '**' 0.01 '*' 0.05 '.' 0.1 ' ' 1
## 
## Residual standard error: 0.6297 on 1669 degrees of freedom
##   (12 Beobachtungen als fehlend gelöscht)
## Multiple R-squared:  0.04693,    Adjusted R-squared:  0.04521 
## F-statistic: 27.39 on 3 and 1669 DF,  p-value: < 2.2e-16
```

```
summary(lm_TMS_via_Uni_TMS_3)
```

```
## 
## Call:
## lm(formula = PCGPA ~ Gender + Age + GPA + TMS_total, data = data_TMS_via_Uni_TMS)
## 
## Residuals:
##     Min      1Q  Median      3Q     Max 
## -1.5319 -0.4397 -0.0809  0.4133  1.8683 
## 
## Coefficients:
##              Estimate Std. Error t value Pr(>|t|)    
## (Intercept)  3.332620   0.268006  12.435  < 2e-16 ***
## Gender      -0.147679   0.033797  -4.370 1.32e-05 ***
## Age          0.005984   0.007522   0.796    0.426    
## GPA          0.172938   0.041908   4.127 3.86e-05 ***
## TMS_total   -0.012416   0.002004  -6.197 7.25e-10 ***
## ---
## Signif. codes:  0 '***' 0.001 '**' 0.01 '*' 0.05 '.' 0.1 ' ' 1
## 
## Residual standard error: 0.6267 on 1668 degrees of freedom
##   (12 Beobachtungen als fehlend gelöscht)
## Multiple R-squared:  0.05656,    Adjusted R-squared:  0.0543 
## F-statistic:    25 on 4 and 1668 DF,  p-value: < 2.2e-16
```

```
anova(lm_TMS_via_Uni_TMS_1, lm_TMS_via_Uni_TMS_2a, lm_TMS_via_Uni_TMS_3)
```

```
## Analysis of Variance Table
## 
## Model 1: PCGPA ~ Gender + Age
## Model 2: PCGPA ~ Gender + Age + GPA
## Model 3: PCGPA ~ Gender + Age + GPA + TMS_total
##   Res.Df    RSS Df Sum of Sq      F    Pr(>F)    
## 1   1670 676.77                                  
## 2   1669 670.14  1    6.6288 16.879 4.177e-05 ***
## 3   1668 655.06  1   15.0799 38.398 7.253e-10 ***
## ---
## Signif. codes:  0 '***' 0.001 '**' 0.01 '*' 0.05 '.' 0.1 ' ' 1
```

```
anova(lm_TMS_via_Uni_TMS_1, lm_TMS_via_Uni_TMS_2b, lm_TMS_via_Uni_TMS_3)
```

```
## Analysis of Variance Table
## 
## Model 1: PCGPA ~ Gender + Age
## Model 2: PCGPA ~ Gender + Age + TMS_total
## Model 3: PCGPA ~ Gender + Age + GPA + TMS_total
##   Res.Df    RSS Df Sum of Sq      F    Pr(>F)    
## 1   1670 676.77                                  
## 2   1669 661.75  1   15.0212 38.249 7.816e-10 ***
## 3   1668 655.06  1    6.6875 17.029 3.864e-05 ***
## ---
## Signif. codes:  0 '***' 0.001 '**' 0.01 '*' 0.05 '.' 0.1 ' ' 1
```

```
#Criterion: M1

#Models
lm_TMS_via_Uni_TMS_M1_1 <- lm(M1 ~ Gender + Age, data = data_TMS_via_Uni_TMS)
lm_TMS_via_Uni_TMS_M1_2a <- lm(M1 ~ Gender + Age + GPA, data = data_TMS_via_Uni_TMS)
lm_TMS_via_Uni_TMS_M1_2b <- lm(M1 ~ Gender + Age + TMS_total, data = data_TMS_via_Uni_TMS)
lm_TMS_via_Uni_TMS_M1_3 <- lm(M1 ~ Gender + Age + GPA + TMS_total, data = data_TMS_via_Uni_TMS)

#Results
summary(lm_TMS_via_Uni_TMS_M1_1)
```

```
## 
## Call:
## lm(formula = M1 ~ Gender + Age, data = data_TMS_via_Uni_TMS)
## 
## Residuals:
##      Min       1Q   Median       3Q      Max 
## -1.58260 -0.52371  0.03519  0.53519  2.53519 
## 
## Coefficients:
##             Estimate Std. Error t value Pr(>|t|)    
## (Intercept)  1.55919    0.37239   4.187 3.78e-05 ***
## Gender      -0.21343    0.10256  -2.081 0.038330 *  
## Age          0.05890    0.01741   3.384 0.000816 ***
## ---
## Signif. codes:  0 '***' 0.001 '**' 0.01 '*' 0.05 '.' 0.1 ' ' 1
## 
## Residual standard error: 0.7699 on 282 degrees of freedom
##   (1400 Beobachtungen als fehlend gelöscht)
## Multiple R-squared:  0.05001,    Adjusted R-squared:  0.04328 
## F-statistic: 7.423 on 2 and 282 DF,  p-value: 0.0007213
```

```
summary(lm_TMS_via_Uni_TMS_M1_2a)
```

```
## 
## Call:
## lm(formula = M1 ~ Gender + Age + GPA, data = data_TMS_via_Uni_TMS)
## 
## Residuals:
##      Min       1Q   Median       3Q      Max 
## -1.64239 -0.54869  0.00469  0.50957  2.55325 
## 
## Coefficients:
##             Estimate Std. Error t value Pr(>|t|)    
## (Intercept)  1.71712    0.38320   4.481 1.08e-05 ***
## Gender      -0.23163    0.10282  -2.253   0.0251 *  
## Age          0.03398    0.02293   1.482   0.1395    
## GPA          0.24279    0.14605   1.662   0.0976 .  
## ---
## Signif. codes:  0 '***' 0.001 '**' 0.01 '*' 0.05 '.' 0.1 ' ' 1
## 
## Residual standard error: 0.7675 on 281 degrees of freedom
##   (1400 Beobachtungen als fehlend gelöscht)
## Multiple R-squared:  0.05927,    Adjusted R-squared:  0.04922 
## F-statistic: 5.901 on 3 and 281 DF,  p-value: 0.0006435
```

```
summary(lm_TMS_via_Uni_TMS_M1_2b)
```

```
## 
## Call:
## lm(formula = M1 ~ Gender + Age + TMS_total, data = data_TMS_via_Uni_TMS)
## 
## Residuals:
##      Min       1Q   Median       3Q      Max 
## -1.73632 -0.57976  0.02786  0.54711  2.54016 
## 
## Coefficients:
##              Estimate Std. Error t value Pr(>|t|)    
## (Intercept)  3.787994   0.747109   5.070 7.22e-07 ***
## Gender      -0.214313   0.100665  -2.129 0.034126 *  
## Age          0.050813   0.017247   2.946 0.003486 ** 
## TMS_total   -0.019253   0.005628  -3.421 0.000718 ***
## ---
## Signif. codes:  0 '***' 0.001 '**' 0.01 '*' 0.05 '.' 0.1 ' ' 1
## 
## Residual standard error: 0.7557 on 281 degrees of freedom
##   (1400 Beobachtungen als fehlend gelöscht)
## Multiple R-squared:  0.08799,    Adjusted R-squared:  0.07825 
## F-statistic: 9.037 on 3 and 281 DF,  p-value: 9.88e-06
```

```
summary(lm_TMS_via_Uni_TMS_M1_3)
```

```
## 
## Call:
## lm(formula = M1 ~ Gender + Age + GPA + TMS_total, data = data_TMS_via_Uni_TMS)
## 
## Residuals:
##      Min       1Q   Median       3Q      Max 
## -1.69157 -0.57428  0.01065  0.55980  2.55644 
## 
## Coefficients:
##              Estimate Std. Error t value Pr(>|t|)    
## (Intercept)  3.884559   0.747973   5.193 3.98e-07 ***
## Gender      -0.230804   0.100998  -2.285 0.023047 *  
## Age          0.028379   0.022583   1.257 0.209933    
## GPA          0.220251   0.143615   1.534 0.126253    
## TMS_total   -0.018849   0.005621  -3.353 0.000909 ***
## ---
## Signif. codes:  0 '***' 0.001 '**' 0.01 '*' 0.05 '.' 0.1 ' ' 1
## 
## Residual standard error: 0.7539 on 280 degrees of freedom
##   (1400 Beobachtungen als fehlend gelöscht)
## Multiple R-squared:  0.09559,    Adjusted R-squared:  0.08267 
## F-statistic: 7.398 on 4 and 280 DF,  p-value: 1.12e-05
```

```
anova(lm_TMS_via_Uni_TMS_M1_1, lm_TMS_via_Uni_TMS_M1_2a, lm_TMS_via_Uni_TMS_M1_3)
```

```
## Analysis of Variance Table
## 
## Model 1: M1 ~ Gender + Age
## Model 2: M1 ~ Gender + Age + GPA
## Model 3: M1 ~ Gender + Age + GPA + TMS_total
##   Res.Df    RSS Df Sum of Sq       F    Pr(>F)    
## 1    282 167.17                                   
## 2    281 165.54  1    1.6280  2.8642 0.0916812 .  
## 3    280 159.15  1    6.3912 11.2445 0.0009087 ***
## ---
## Signif. codes:  0 '***' 0.001 '**' 0.01 '*' 0.05 '.' 0.1 ' ' 1
```

```
anova(lm_TMS_via_Uni_TMS_M1_1, lm_TMS_via_Uni_TMS_M1_2b, lm_TMS_via_Uni_TMS_M1_3)
```

```
## Analysis of Variance Table
## 
## Model 1: M1 ~ Gender + Age
## Model 2: M1 ~ Gender + Age + TMS_total
## Model 3: M1 ~ Gender + Age + GPA + TMS_total
##   Res.Df    RSS Df Sum of Sq      F    Pr(>F)    
## 1    282 167.17                                  
## 2    281 160.49  1    6.6824 11.757 0.0006974 ***
## 3    280 159.15  1    1.3368  2.352 0.1262531    
## ---
## Signif. codes:  0 '***' 0.001 '**' 0.01 '*' 0.05 '.' 0.1 ' ' 1
```

#### 3.3.1.2 HAM-Nat

```
#Criterion: PCGPA

#Models
lm_HAMNat_via_Uni_TMS_1 <- lm(PCGPA ~ Gender + Age, data = data_HAMNat_via_Uni_TMS)
lm_HAMNat_via_Uni_TMS_2a <- lm(PCGPA ~ Gender + Age + GPA, data = data_HAMNat_via_Uni_TMS)
lm_HAMNat_via_Uni_TMS_2b <- lm(PCGPA ~ Gender + Age + HAMNat_total, data = data_HAMNat_via_Uni_TMS)
lm_HAMNat_via_Uni_TMS_3 <- lm(PCGPA ~ Gender + Age + GPA + HAMNat_total, data = data_HAMNat_via_Uni_TMS)

#Results
summary(lm_HAMNat_via_Uni_TMS_1)
```

```
## 
## Call:
## lm(formula = PCGPA ~ Gender + Age, data = data_HAMNat_via_Uni_TMS)
## 
## Residuals:
##     Min      1Q  Median      3Q     Max 
## -1.2550 -0.4541 -0.1540  0.4451  1.7956 
## 
## Coefficients:
##               Estimate Std. Error t value Pr(>|t|)    
## (Intercept)  2.3024791  0.2762951   8.333 1.36e-15 ***
## Gender      -0.0505769  0.0678616  -0.745    0.457    
## Age          0.0001109  0.0124167   0.009    0.993    
## ---
## Signif. codes:  0 '***' 0.001 '**' 0.01 '*' 0.05 '.' 0.1 ' ' 1
## 
## Residual standard error: 0.622 on 390 degrees of freedom
##   (5 Beobachtungen als fehlend gelöscht)
## Multiple R-squared:  0.001423,   Adjusted R-squared:  -0.003698 
## F-statistic: 0.2779 on 2 and 390 DF,  p-value: 0.7575
```

```
summary(lm_HAMNat_via_Uni_TMS_2a)
```

```
## 
## Call:
## lm(formula = PCGPA ~ Gender + Age + GPA, data = data_HAMNat_via_Uni_TMS)
## 
## Residuals:
##     Min      1Q  Median      3Q     Max 
## -1.2843 -0.4289 -0.1194  0.4073  1.7973 
## 
## Coefficients:
##             Estimate Std. Error t value Pr(>|t|)    
## (Intercept)  2.36504    0.27734   8.528 3.36e-16 ***
## Gender      -0.06276    0.06794  -0.924   0.3562    
## Age         -0.01491    0.01469  -1.015   0.3107    
## GPA          0.16311    0.08590   1.899   0.0583 .  
## ---
## Signif. codes:  0 '***' 0.001 '**' 0.01 '*' 0.05 '.' 0.1 ' ' 1
## 
## Residual standard error: 0.62 on 389 degrees of freedom
##   (5 Beobachtungen als fehlend gelöscht)
## Multiple R-squared:  0.01059,    Adjusted R-squared:  0.002964 
## F-statistic: 1.388 on 3 and 389 DF,  p-value: 0.2459
```

```
summary(lm_HAMNat_via_Uni_TMS_2b)
```

```
## 
## Call:
## lm(formula = PCGPA ~ Gender + Age + HAMNat_total, data = data_HAMNat_via_Uni_TMS)
## 
## Residuals:
##      Min       1Q   Median       3Q      Max 
## -1.26239 -0.38399 -0.08203  0.42704  1.69706 
## 
## Coefficients:
##               Estimate Std. Error t value Pr(>|t|)    
## (Intercept)   2.470803   0.265799   9.296  < 2e-16 ***
## Gender        0.015795   0.065839   0.240    0.811    
## Age          -0.008218   0.011959  -0.687    0.492    
## HAMNat_total -0.221479   0.036399  -6.085 2.79e-09 ***
## ---
## Signif. codes:  0 '***' 0.001 '**' 0.01 '*' 0.05 '.' 0.1 ' ' 1
## 
## Residual standard error: 0.5951 on 389 degrees of freedom
##   (5 Beobachtungen als fehlend gelöscht)
## Multiple R-squared:  0.08821,    Adjusted R-squared:  0.08118 
## F-statistic: 12.54 on 3 and 389 DF,  p-value: 7.608e-08
```

```
summary(lm_HAMNat_via_Uni_TMS_3)
```

```
## 
## Call:
## lm(formula = PCGPA ~ Gender + Age + GPA + HAMNat_total, data = data_HAMNat_via_Uni_TMS)
## 
## Residuals:
##      Min       1Q   Median       3Q      Max 
## -1.24281 -0.40017 -0.08544  0.43064  1.66126 
## 
## Coefficients:
##               Estimate Std. Error t value Pr(>|t|)    
## (Intercept)   2.516118   0.266986   9.424  < 2e-16 ***
## Gender        0.005111   0.066095   0.077    0.938    
## Age          -0.019698   0.014097  -1.397    0.163    
## GPA           0.126388   0.082549   1.531    0.127    
## HAMNat_total -0.217318   0.036437  -5.964 5.54e-09 ***
## ---
## Signif. codes:  0 '***' 0.001 '**' 0.01 '*' 0.05 '.' 0.1 ' ' 1
## 
## Residual standard error: 0.5941 on 388 degrees of freedom
##   (5 Beobachtungen als fehlend gelöscht)
## Multiple R-squared:  0.09368,    Adjusted R-squared:  0.08434 
## F-statistic: 10.03 on 4 and 388 DF,  p-value: 9.885e-08
```

```
anova(lm_HAMNat_via_Uni_TMS_1, lm_HAMNat_via_Uni_TMS_2a, lm_HAMNat_via_Uni_TMS_3)
```

```
## Analysis of Variance Table
## 
## Model 1: PCGPA ~ Gender + Age
## Model 2: PCGPA ~ Gender + Age + GPA
## Model 3: PCGPA ~ Gender + Age + GPA + HAMNat_total
##   Res.Df    RSS Df Sum of Sq       F    Pr(>F)    
## 1    390 150.90                                   
## 2    389 149.51  1    1.3859  3.9263   0.04824 *  
## 3    388 136.96  1   12.5559 35.5712 5.538e-09 ***
## ---
## Signif. codes:  0 '***' 0.001 '**' 0.01 '*' 0.05 '.' 0.1 ' ' 1
```

```
anova(lm_HAMNat_via_Uni_TMS_1, lm_HAMNat_via_Uni_TMS_2b, lm_HAMNat_via_Uni_TMS_3)
```

```
## Analysis of Variance Table
## 
## Model 1: PCGPA ~ Gender + Age
## Model 2: PCGPA ~ Gender + Age + HAMNat_total
## Model 3: PCGPA ~ Gender + Age + GPA + HAMNat_total
##   Res.Df    RSS Df Sum of Sq       F    Pr(>F)    
## 1    390 150.90                                   
## 2    389 137.78  1   13.1144 37.1533 2.637e-09 ***
## 3    388 136.96  1    0.8275  2.3442    0.1266    
## ---
## Signif. codes:  0 '***' 0.001 '**' 0.01 '*' 0.05 '.' 0.1 ' ' 1
```

```
#Criterion: M1

#Models
lm_HAMNat_via_Uni_TMS_M1_1 <- lm(M1 ~ Gender + Age, data = data_HAMNat_via_Uni_TMS)
lm_HAMNat_via_Uni_TMS_M1_2a <- lm(M1 ~ Gender + Age + GPA, data = data_HAMNat_via_Uni_TMS)
lm_HAMNat_via_Uni_TMS_M1_2b <- lm(M1 ~ Gender + Age + HAMNat_total, data = data_HAMNat_via_Uni_TMS)
lm_HAMNat_via_Uni_TMS_M1_3 <- lm(M1 ~ Gender + Age + GPA + HAMNat_total, data = data_HAMNat_via_Uni_TMS)

#Results
summary(lm_HAMNat_via_Uni_TMS_M1_1)
```

```
## 
## Call:
## lm(formula = M1 ~ Gender + Age, data = data_HAMNat_via_Uni_TMS)
## 
## Residuals:
##      Min       1Q   Median       3Q      Max 
## -1.52784 -0.58526 -0.02784  0.52332  1.59691 
## 
## Coefficients:
##             Estimate Std. Error t value Pr(>|t|)  
## (Intercept)  1.53031    0.65077   2.352   0.0205 *
## Gender      -0.12475    0.16339  -0.764   0.4468  
## Age          0.05907    0.03092   1.910   0.0588 .
## ---
## Signif. codes:  0 '***' 0.001 '**' 0.01 '*' 0.05 '.' 0.1 ' ' 1
## 
## Residual standard error: 0.7728 on 107 degrees of freedom
##   (288 Beobachtungen als fehlend gelöscht)
## Multiple R-squared:  0.0346, Adjusted R-squared:  0.01655 
## F-statistic: 1.917 on 2 and 107 DF,  p-value: 0.152
```

```
summary(lm_HAMNat_via_Uni_TMS_M1_2a)
```

```
## 
## Call:
## lm(formula = M1 ~ Gender + Age + GPA, data = data_HAMNat_via_Uni_TMS)
## 
## Residuals:
##      Min       1Q   Median       3Q      Max 
## -1.50919 -0.57744 -0.02765  0.50797  1.62727 
## 
## Coefficients:
##             Estimate Std. Error t value Pr(>|t|)  
## (Intercept)  1.51132    0.66119   2.286   0.0243 *
## Gender      -0.11930    0.16658  -0.716   0.4755  
## Age          0.06309    0.03749   1.683   0.0954 .
## GPA         -0.04292    0.22403  -0.192   0.8484  
## ---
## Signif. codes:  0 '***' 0.001 '**' 0.01 '*' 0.05 '.' 0.1 ' ' 1
## 
## Residual standard error: 0.7763 on 106 degrees of freedom
##   (288 Beobachtungen als fehlend gelöscht)
## Multiple R-squared:  0.03493,    Adjusted R-squared:  0.007619 
## F-statistic: 1.279 on 3 and 106 DF,  p-value: 0.2854
```

```
summary(lm_HAMNat_via_Uni_TMS_M1_2b)
```

```
## 
## Call:
## lm(formula = M1 ~ Gender + Age + HAMNat_total, data = data_HAMNat_via_Uni_TMS)
## 
## Residuals:
##      Min       1Q   Median       3Q      Max 
## -1.64689 -0.44191 -0.03986  0.55008  1.82072 
## 
## Coefficients:
##              Estimate Std. Error t value Pr(>|t|)    
## (Intercept)   2.41976    0.62008   3.902 0.000168 ***
## Gender       -0.05152    0.14942  -0.345 0.730921    
## Age           0.02191    0.02917   0.751 0.454086    
## HAMNat_total -0.41707    0.08644  -4.825 4.72e-06 ***
## ---
## Signif. codes:  0 '***' 0.001 '**' 0.01 '*' 0.05 '.' 0.1 ' ' 1
## 
## Residual standard error: 0.7031 on 106 degrees of freedom
##   (288 Beobachtungen als fehlend gelöscht)
## Multiple R-squared:  0.2084, Adjusted R-squared:  0.186 
## F-statistic: 9.304 on 3 and 106 DF,  p-value: 1.626e-05
```

```
summary(lm_HAMNat_via_Uni_TMS_M1_3)
```

```
## 
## Call:
## lm(formula = M1 ~ Gender + Age + GPA + HAMNat_total, data = data_HAMNat_via_Uni_TMS)
## 
## Residuals:
##      Min       1Q   Median       3Q      Max 
## -1.65110 -0.44741 -0.03945  0.55857  1.79808 
## 
## Coefficients:
##              Estimate Std. Error t value Pr(>|t|)    
## (Intercept)   2.43658    0.63170   3.857 0.000198 ***
## Gender       -0.05550    0.15215  -0.365 0.715994    
## Age           0.01874    0.03534   0.530 0.596993    
## GPA           0.03282    0.20444   0.161 0.872777    
## HAMNat_total -0.41815    0.08710  -4.801 5.26e-06 ***
## ---
## Signif. codes:  0 '***' 0.001 '**' 0.01 '*' 0.05 '.' 0.1 ' ' 1
## 
## Residual standard error: 0.7063 on 105 degrees of freedom
##   (288 Beobachtungen als fehlend gelöscht)
## Multiple R-squared:  0.2086, Adjusted R-squared:  0.1785 
## F-statistic: 6.921 on 4 and 105 DF,  p-value: 5.523e-05
```

```
anova(lm_HAMNat_via_Uni_TMS_M1_1, lm_HAMNat_via_Uni_TMS_M1_2a, lm_HAMNat_via_Uni_TMS_M1_3)
```

```
## Analysis of Variance Table
## 
## Model 1: M1 ~ Gender + Age
## Model 2: M1 ~ Gender + Age + GPA
## Model 3: M1 ~ Gender + Age + GPA + HAMNat_total
##   Res.Df    RSS Df Sum of Sq       F    Pr(>F)    
## 1    107 63.901                                   
## 2    106 63.879  1    0.0221  0.0443    0.8336    
## 3    105 52.381  1   11.4978 23.0478 5.263e-06 ***
## ---
## Signif. codes:  0 '***' 0.001 '**' 0.01 '*' 0.05 '.' 0.1 ' ' 1
```

```
anova(lm_HAMNat_via_Uni_TMS_M1_1, lm_HAMNat_via_Uni_TMS_M1_2b, lm_HAMNat_via_Uni_TMS_M1_3)
```

```
## Analysis of Variance Table
## 
## Model 1: M1 ~ Gender + Age
## Model 2: M1 ~ Gender + Age + HAMNat_total
## Model 3: M1 ~ Gender + Age + GPA + HAMNat_total
##   Res.Df    RSS Df Sum of Sq       F    Pr(>F)    
## 1    107 63.901                                   
## 2    106 52.394  1   11.5070 23.0663 5.221e-06 ***
## 3    105 52.381  1    0.0129  0.0258    0.8728    
## ---
## Signif. codes:  0 '***' 0.001 '**' 0.01 '*' 0.05 '.' 0.1 ' ' 1
```

### 3.3.2 Regression analyses in universities with selection via TMS (corrected)

#### 3.3.2.1 TMS

```
#Criterion: PCGPA

#Correcting for range restriction
data_TMS_via_Uni_TMS$Gender <- as.numeric(data_TMS_via_Uni_TMS$Gender)
Cov_Mat_TMS_via_Uni_TMS_Incumbents <- cov((dplyr::select(data_TMS_via_Uni_TMS, Gender, Age, GPA, TMS_total, PCGPA)), use = "complete.obs")
Cov_Mat_TMS_via_Uni_TMS_Testtakers <- cov((dplyr::select(data_TMS_Testtakers, Gender, Age, GPA, TMS_total)))
Cor_Mat_TMS_via_Uni_TMS_Incumbents_Corr <- lMvrrc(rcov = Cov_Mat_TMS_via_Uni_TMS_Incumbents, vnp = Cov_Mat_TMS_via_Uni_TMS_Testtakers, as_cor = T)
rownames(Cor_Mat_TMS_via_Uni_TMS_Incumbents_Corr) <- colnames(Cor_Mat_TMS_via_Uni_TMS_Incumbents_Corr) <- c('Gender', 'Age', 'GPA', 'TMS_total', 'PCGPA')

#Models
lm_TMS_via_Uni_TMS_1_corr <- lmCor(PCGPA ~ Gender + Age, data = Cor_Mat_TMS_via_Uni_TMS_Incumbents_Corr, n.obs = nobs(lm_TMS_via_Uni_TMS_1), plot = F)
lm_TMS_via_Uni_TMS_2a_corr <- lmCor(PCGPA ~ Gender + Age + GPA, data = Cor_Mat_TMS_via_Uni_TMS_Incumbents_Corr, n.obs = nobs(lm_TMS_via_Uni_TMS_2a), plot = F)
lm_TMS_via_Uni_TMS_2b_corr <- lmCor(PCGPA ~ Gender + Age + TMS_total, data = Cor_Mat_TMS_via_Uni_TMS_Incumbents_Corr, n.obs = nobs(lm_TMS_via_Uni_TMS_2b), plot = F)
lm_TMS_via_Uni_TMS_3_corr <- lmCor(PCGPA ~ Gender + Age + GPA + TMS_total, data = Cor_Mat_TMS_via_Uni_TMS_Incumbents_Corr, n.obs = nobs(lm_TMS_via_Uni_TMS_3), plot = F)

#Results
lm_TMS_via_Uni_TMS_1_corr
```

```
## Call: lmCor(y = PCGPA ~ Gender + Age, data = Cor_Mat_TMS_via_Uni_TMS_Incumbents_Corr, 
##     n.obs = nobs(lm_TMS_via_Uni_TMS_1), plot = F)
## 
## Multiple Regression from matrix input 
## 
##  DV =  PCGPA 
##        slope   se     t       p lower.ci upper.ci VIF Vy.x
## Gender -0.12 0.02 -4.80 1.8e-06    -0.16    -0.07   1 0.01
## Age     0.11 0.02  4.57 5.3e-06     0.06     0.16   1 0.01
## 
## Residual Standard Error =  0.99  with  1670  degrees of freedom
## 
##  Multiple Regression
##          R   R2  Ruw R2uw Shrunken R2 SE of R2 overall F df1  df2        p
## PCGPA 0.16 0.02 0.16 0.02        0.02     0.01     20.85   2 1670 1.14e-09
```

```
lm_TMS_via_Uni_TMS_2a_corr
```

```
## Call: lmCor(y = PCGPA ~ Gender + Age + GPA, data = Cor_Mat_TMS_via_Uni_TMS_Incumbents_Corr, 
##     n.obs = nobs(lm_TMS_via_Uni_TMS_2a), plot = F)
## 
## Multiple Regression from matrix input 
## 
##  DV =  PCGPA 
##        slope   se     t       p lower.ci upper.ci  VIF Vy.x
## Gender -0.12 0.02 -4.92 9.7e-07    -0.16    -0.07 1.00 0.01
## Age     0.03 0.03  1.22 2.2e-01    -0.02     0.09 1.27 0.00
## GPA     0.17 0.03  6.27 4.6e-10     0.12     0.22 1.27 0.03
## 
## Residual Standard Error =  0.98  with  1669  degrees of freedom
## 
##  Multiple Regression
##          R   R2 Ruw R2uw Shrunken R2 SE of R2 overall F df1  df2        p
## PCGPA 0.22 0.05 0.2 0.04        0.05     0.01     27.33   3 1669 2.98e-17
```

```
lm_TMS_via_Uni_TMS_2b_corr
```

```
## Call: lmCor(y = PCGPA ~ Gender + Age + TMS_total, data = Cor_Mat_TMS_via_Uni_TMS_Incumbents_Corr, 
##     n.obs = nobs(lm_TMS_via_Uni_TMS_2b), plot = F)
## 
## Multiple Regression from matrix input 
## 
##  DV =  PCGPA 
##           slope   se     t       p lower.ci upper.ci  VIF Vy.x
## Gender    -0.10 0.02 -4.05 5.3e-05    -0.14    -0.05 1.01 0.01
## Age        0.07 0.02  3.08 2.1e-03     0.03     0.12 1.04 0.01
## TMS_total -0.21 0.02 -8.50 4.3e-17    -0.25    -0.16 1.04 0.05
## 
## Residual Standard Error =  0.97  with  1669  degrees of freedom
## 
##  Multiple Regression
##          R   R2  Ruw R2uw Shrunken R2 SE of R2 overall F df1  df2        p
## PCGPA 0.25 0.06 0.24 0.06        0.06     0.01     38.55   3 1669 4.38e-24
```

```
lm_TMS_via_Uni_TMS_3_corr
```

```
## Call: lmCor(y = PCGPA ~ Gender + Age + GPA + TMS_total, data = Cor_Mat_TMS_via_Uni_TMS_Incumbents_Corr, 
##     n.obs = nobs(lm_TMS_via_Uni_TMS_3), plot = F)
## 
## Multiple Regression from matrix input 
## 
##  DV =  PCGPA 
##           slope   se     t       p lower.ci upper.ci  VIF Vy.x
## Gender    -0.10 0.02 -4.23 2.5e-05    -0.15    -0.05 1.01 0.01
## Age        0.02 0.03  0.82 4.1e-01    -0.03     0.07 1.28 0.00
## GPA        0.12 0.03  4.55 5.6e-06     0.07     0.18 1.34 0.02
## TMS_total -0.18 0.02 -7.29 4.6e-13    -0.23    -0.13 1.10 0.04
## 
## Residual Standard Error =  0.96  with  1668  degrees of freedom
## 
##  Multiple Regression
##          R   R2  Ruw R2uw Shrunken R2 SE of R2 overall F df1  df2        p
## PCGPA 0.28 0.08 0.26 0.07        0.07     0.01     34.44   4 1668 1.17e-27
```

```
anova(lm_TMS_via_Uni_TMS_1_corr, lm_TMS_via_Uni_TMS_2a_corr, lm_TMS_via_Uni_TMS_3_corr)
```

```
## Model 1 = lmCor(y = PCGPA ~ Gender + Age, data = Cor_Mat_TMS_via_Uni_TMS_Incumbents_Corr, 
##     n.obs = nobs(lm_TMS_via_Uni_TMS_1), plot = F)
## Model 2 = lmCor(y = PCGPA ~ Gender + Age + GPA, data = Cor_Mat_TMS_via_Uni_TMS_Incumbents_Corr, 
##     n.obs = nobs(lm_TMS_via_Uni_TMS_2a), plot = F)
## Model 3 = lmCor(y = PCGPA ~ Gender + Age + GPA + TMS_total, data = Cor_Mat_TMS_via_Uni_TMS_Incumbents_Corr, 
##     n.obs = nobs(lm_TMS_via_Uni_TMS_3), plot = F)
```

```
## $PCGPA
##   Res Df   Res SS Diff df  Diff SS        F     Pr(F > )
## 1   1670 1631.264      NA       NA       NA           NA
## 2   1669 1593.710       1 37.55367 40.55800 2.461458e-10
## 3   1668 1544.443       1 49.26758 53.20905 4.614908e-13
```

```
anova(lm_TMS_via_Uni_TMS_1_corr, lm_TMS_via_Uni_TMS_2b_corr, lm_TMS_via_Uni_TMS_3_corr)
```

```
## Model 1 = lmCor(y = PCGPA ~ Gender + Age, data = Cor_Mat_TMS_via_Uni_TMS_Incumbents_Corr, 
##     n.obs = nobs(lm_TMS_via_Uni_TMS_1), plot = F)
## Model 2 = lmCor(y = PCGPA ~ Gender + Age + TMS_total, data = Cor_Mat_TMS_via_Uni_TMS_Incumbents_Corr, 
##     n.obs = nobs(lm_TMS_via_Uni_TMS_2b), plot = F)
## Model 3 = lmCor(y = PCGPA ~ Gender + Age + GPA + TMS_total, data = Cor_Mat_TMS_via_Uni_TMS_Incumbents_Corr, 
##     n.obs = nobs(lm_TMS_via_Uni_TMS_3), plot = F)
```

```
## $PCGPA
##   Res Df   Res SS Diff df  Diff SS        F     Pr(F > )
## 1   1670 1631.264      NA       NA       NA           NA
## 2   1669 1563.650       1 67.61384 73.02303 2.844690e-17
## 3   1668 1544.443       1 19.20741 20.74403 5.630356e-06
```

```
#Criterion: M1

#Correcting for range restriction
Cov_Mat_TMS_via_Uni_TMS_Incumbents_M1 <- cov((dplyr::select(data_TMS_via_Uni_TMS, Gender, Age, GPA, TMS_total, M1)), use = "complete.obs")
Cor_Mat_TMS_via_Uni_TMS_Incumbents_Corr_M1 <- lMvrrc(rcov = Cov_Mat_TMS_via_Uni_TMS_Incumbents_M1, vnp = Cov_Mat_TMS_via_Uni_TMS_Testtakers, as_cor = T)
rownames(Cor_Mat_TMS_via_Uni_TMS_Incumbents_Corr_M1) <- colnames(Cor_Mat_TMS_via_Uni_TMS_Incumbents_Corr_M1) <- c('Gender', 'Age', 'GPA', 'TMS_total', 'M1')

#Models
lm_TMS_via_Uni_TMS_M1_1_corr <- lmCor(M1 ~ Gender + Age, data = Cor_Mat_TMS_via_Uni_TMS_Incumbents_Corr_M1, n.obs = nobs(lm_TMS_via_Uni_TMS_M1_1), plot = F)
lm_TMS_via_Uni_TMS_M1_2a_corr <- lmCor(M1 ~ Gender + Age + GPA, data = Cor_Mat_TMS_via_Uni_TMS_Incumbents_Corr_M1, n.obs = nobs(lm_TMS_via_Uni_TMS_M1_2a), plot = F)
lm_TMS_via_Uni_TMS_M1_2b_corr <- lmCor(M1 ~ Gender + Age + TMS_total, data = Cor_Mat_TMS_via_Uni_TMS_Incumbents_Corr_M1, n.obs = nobs(lm_TMS_via_Uni_TMS_M1_2b), plot = F)
lm_TMS_via_Uni_TMS_M1_3_corr <- lmCor(M1 ~ Gender + Age + GPA + TMS_total, data = Cor_Mat_TMS_via_Uni_TMS_Incumbents_Corr_M1, n.obs = nobs(lm_TMS_via_Uni_TMS_M1_3), plot = F)

#Results
lm_TMS_via_Uni_TMS_M1_1_corr
```

```
## Call: lmCor(y = M1 ~ Gender + Age, data = Cor_Mat_TMS_via_Uni_TMS_Incumbents_Corr_M1, 
##     n.obs = nobs(lm_TMS_via_Uni_TMS_M1_1), plot = F)
## 
## Multiple Regression from matrix input 
## 
##  DV =  M1 
##        slope   se     t      p lower.ci upper.ci VIF Vy.x
## Gender -0.15 0.06 -2.54 0.0120    -0.26    -0.03   1 0.02
## Age     0.18 0.06  3.14 0.0019     0.07     0.30   1 0.03
## 
## Residual Standard Error =  0.98  with  282  degrees of freedom
## 
##  Multiple Regression
##       R   R2  Ruw R2uw Shrunken R2 SE of R2 overall F df1 df2        p
## M1 0.23 0.05 0.23 0.05        0.05     0.03      7.76   2 282 0.000522
```

```
lm_TMS_via_Uni_TMS_M1_2a_corr
```

```
## Call: lmCor(y = M1 ~ Gender + Age + GPA, data = Cor_Mat_TMS_via_Uni_TMS_Incumbents_Corr_M1, 
##     n.obs = nobs(lm_TMS_via_Uni_TMS_M1_2a), plot = F)
## 
## Multiple Regression from matrix input 
## 
##  DV =  M1 
##        slope   se     t      p lower.ci upper.ci  VIF Vy.x
## Gender -0.15 0.06 -2.60 0.0098    -0.26    -0.04 1.00 0.02
## Age     0.10 0.06  1.51 0.1300    -0.03     0.22 1.27 0.02
## GPA     0.18 0.06  2.85 0.0046     0.06     0.31 1.27 0.04
## 
## Residual Standard Error =  0.96  with  281  degrees of freedom
## 
##  Multiple Regression
##       R   R2  Ruw R2uw Shrunken R2 SE of R2 overall F df1 df2       p
## M1 0.28 0.08 0.28 0.08        0.07     0.03      8.02   3 281 3.8e-05
```

```
lm_TMS_via_Uni_TMS_M1_2b_corr
```

```
## Call: lmCor(y = M1 ~ Gender + Age + TMS_total, data = Cor_Mat_TMS_via_Uni_TMS_Incumbents_Corr_M1, 
##     n.obs = nobs(lm_TMS_via_Uni_TMS_M1_2b), plot = F)
## 
## Multiple Regression from matrix input 
## 
##  DV =  M1 
##           slope   se     t       p lower.ci upper.ci  VIF Vy.x
## Gender    -0.12 0.06 -2.19 3.0e-02    -0.24    -0.01 1.01 0.02
## Age        0.14 0.06  2.42 1.6e-02     0.03     0.25 1.04 0.02
## TMS_total -0.25 0.06 -4.33 2.1e-05    -0.36    -0.14 1.04 0.07
## 
## Residual Standard Error =  0.95  with  281  degrees of freedom
## 
##  Multiple Regression
##       R   R2  Ruw R2uw Shrunken R2 SE of R2 overall F df1 df2        p
## M1 0.33 0.11 0.32  0.1         0.1     0.03     11.76   3 281 2.79e-07
```

```
lm_TMS_via_Uni_TMS_M1_3_corr
```

```
## Call: lmCor(y = M1 ~ Gender + Age + GPA + TMS_total, data = Cor_Mat_TMS_via_Uni_TMS_Incumbents_Corr_M1, 
##     n.obs = nobs(lm_TMS_via_Uni_TMS_M1_3), plot = F)
## 
## Multiple Regression from matrix input 
## 
##  DV =  M1 
##           slope   se     t       p lower.ci upper.ci  VIF Vy.x
## Gender    -0.13 0.06 -2.26 0.02400    -0.24    -0.02 1.01 0.02
## Age        0.08 0.06  1.33 0.19000    -0.04     0.21 1.28 0.01
## GPA        0.13 0.06  1.99 0.04800     0.00     0.26 1.34 0.03
## TMS_total -0.22 0.06 -3.79 0.00018    -0.34    -0.11 1.10 0.06
## 
## Residual Standard Error =  0.94  with  280  degrees of freedom
## 
##  Multiple Regression
##       R   R2  Ruw R2uw Shrunken R2 SE of R2 overall F df1 df2        p
## M1 0.35 0.12 0.34 0.12        0.11     0.04       9.9   4 280 1.66e-07
```

```
anova(lm_TMS_via_Uni_TMS_M1_1_corr, lm_TMS_via_Uni_TMS_M1_2a_corr, lm_TMS_via_Uni_TMS_M1_3_corr)
```

```
## Model 1 = lmCor(y = M1 ~ Gender + Age, data = Cor_Mat_TMS_via_Uni_TMS_Incumbents_Corr_M1, 
##     n.obs = nobs(lm_TMS_via_Uni_TMS_M1_1), plot = F)
## Model 2 = lmCor(y = M1 ~ Gender + Age + GPA, data = Cor_Mat_TMS_via_Uni_TMS_Incumbents_Corr_M1, 
##     n.obs = nobs(lm_TMS_via_Uni_TMS_M1_2a), plot = F)
## Model 3 = lmCor(y = M1 ~ Gender + Age + GPA + TMS_total, data = Cor_Mat_TMS_via_Uni_TMS_Incumbents_Corr_M1, 
##     n.obs = nobs(lm_TMS_via_Uni_TMS_M1_3), plot = F)
```

```
## $M1
##   Res Df   Res SS Diff df   Diff SS         F     Pr(F > )
## 1    282 269.1772      NA        NA        NA           NA
## 2    281 261.5984       1  7.578805  8.528861 0.0037789560
## 3    280 248.8099       1 12.788416 14.391533 0.0001819423
```

```
anova(lm_TMS_via_Uni_TMS_M1_1_corr, lm_TMS_via_Uni_TMS_M1_2b_corr, lm_TMS_via_Uni_TMS_M1_3_corr)
```

```
## Model 1 = lmCor(y = M1 ~ Gender + Age, data = Cor_Mat_TMS_via_Uni_TMS_Incumbents_Corr_M1, 
##     n.obs = nobs(lm_TMS_via_Uni_TMS_M1_1), plot = F)
## Model 2 = lmCor(y = M1 ~ Gender + Age + TMS_total, data = Cor_Mat_TMS_via_Uni_TMS_Incumbents_Corr_M1, 
##     n.obs = nobs(lm_TMS_via_Uni_TMS_M1_2b), plot = F)
## Model 3 = lmCor(y = M1 ~ Gender + Age + GPA + TMS_total, data = Cor_Mat_TMS_via_Uni_TMS_Incumbents_Corr_M1, 
##     n.obs = nobs(lm_TMS_via_Uni_TMS_M1_3), plot = F)
```

```
## $M1
##   Res Df   Res SS Diff df   Diff SS         F     Pr(F > )
## 1    282 269.1772      NA        NA        NA           NA
## 2    281 252.3189       1 16.858217 18.971513 1.861425e-05
## 3    280 248.8099       1  3.509003  3.948881 4.787676e-02
```

#### 3.3.2.2 HAM-Nat

```
#Criterion: PCGPA

#Correcting for range restriction
Cov_Mat_HAMNat_via_Uni_TMS_Incumbents <- cov((dplyr::select(data_HAMNat_via_Uni_TMS, Gender, Age, GPA, HAMNat_total, PCGPA)), use = "complete.obs")
Cov_Mat_HAMNat_via_Uni_TMS_Testtakers <- cov((dplyr::select(data_HAMNat_Testtakers, Gender, Age, GPA, HAMNat_total)))
Cor_Mat_HAMNat_via_Uni_TMS_Incumbents_Corr <- lMvrrc(rcov = Cov_Mat_HAMNat_via_Uni_TMS_Incumbents, vnp = Cov_Mat_HAMNat_via_Uni_TMS_Testtakers, as_cor = T)
rownames(Cor_Mat_HAMNat_via_Uni_TMS_Incumbents_Corr) <- colnames(Cor_Mat_HAMNat_via_Uni_TMS_Incumbents_Corr) <- c('Gender', 'Age', 'GPA', 'HAMNat_total', 'PCGPA')

#Models
lm_HAMNat_via_Uni_TMS_1_corr <- lmCor(PCGPA ~ Gender + Age, data = Cor_Mat_HAMNat_via_Uni_TMS_Incumbents_Corr, n.obs = nobs(lm_HAMNat_via_Uni_TMS_1), plot = F)
lm_HAMNat_via_Uni_TMS_2a_corr <- lmCor(PCGPA ~ Gender + Age + GPA, data = Cor_Mat_HAMNat_via_Uni_TMS_Incumbents_Corr, n.obs = nobs(lm_HAMNat_via_Uni_TMS_2a), plot = F)
lm_HAMNat_via_Uni_TMS_2b_corr <- lmCor(PCGPA ~ Gender + Age + HAMNat_total, data = Cor_Mat_HAMNat_via_Uni_TMS_Incumbents_Corr, n.obs = nobs(lm_HAMNat_via_Uni_TMS_2b), plot = F)
lm_HAMNat_via_Uni_TMS_3_corr <- lmCor(PCGPA ~ Gender + Age + GPA + HAMNat_total, data = Cor_Mat_HAMNat_via_Uni_TMS_Incumbents_Corr, n.obs = nobs(lm_HAMNat_via_Uni_TMS_3), plot = F)

#Results
lm_HAMNat_via_Uni_TMS_1_corr
```

```
## Call: lmCor(y = PCGPA ~ Gender + Age, data = Cor_Mat_HAMNat_via_Uni_TMS_Incumbents_Corr, 
##     n.obs = nobs(lm_HAMNat_via_Uni_TMS_1), plot = F)
## 
## Multiple Regression from matrix input 
## 
##  DV =  PCGPA 
##        slope   se     t    p lower.ci upper.ci VIF Vy.x
## Gender -0.05 0.05 -1.09 0.28    -0.15     0.04   1    0
## Age    -0.03 0.05 -0.53 0.59    -0.13     0.07   1    0
## 
## Residual Standard Error =  1  with  390  degrees of freedom
## 
##  Multiple Regression
##          R R2  Ruw R2uw Shrunken R2 SE of R2 overall F df1 df2     p
## PCGPA 0.06  0 0.06    0           0     0.01      0.74   2 390 0.475
```

```
lm_HAMNat_via_Uni_TMS_2a_corr
```

```
## Call: lmCor(y = PCGPA ~ Gender + Age + GPA, data = Cor_Mat_HAMNat_via_Uni_TMS_Incumbents_Corr, 
##     n.obs = nobs(lm_HAMNat_via_Uni_TMS_2a), plot = F)
## 
## Multiple Regression from matrix input 
## 
##  DV =  PCGPA 
##        slope   se     t       p lower.ci upper.ci VIF Vy.x
## Gender -0.06 0.05 -1.17 0.24000    -0.16     0.04 1.0 0.00
## Age    -0.11 0.05 -1.93 0.05500    -0.21     0.00 1.2 0.00
## GPA     0.19 0.05  3.51 0.00049     0.08     0.30 1.2 0.03
## 
## Residual Standard Error =  0.99  with  389  degrees of freedom
## 
##  Multiple Regression
##          R   R2  Ruw R2uw Shrunken R2 SE of R2 overall F df1 df2      p
## PCGPA 0.19 0.03 0.16 0.02        0.03     0.02      4.63   3 389 0.0034
```

```
lm_HAMNat_via_Uni_TMS_2b_corr
```

```
## Call: lmCor(y = PCGPA ~ Gender + Age + HAMNat_total, data = Cor_Mat_HAMNat_via_Uni_TMS_Incumbents_Corr, 
##     n.obs = nobs(lm_HAMNat_via_Uni_TMS_2b), plot = F)
## 
## Multiple Regression from matrix input 
## 
##  DV =  PCGPA 
##              slope   se     t       p lower.ci upper.ci  VIF Vy.x
## Gender        0.01 0.05  0.22 8.2e-01    -0.09     0.11 1.04 0.00
## Age          -0.04 0.05 -0.93 3.5e-01    -0.14     0.05 1.00 0.00
## HAMNat_total -0.33 0.05 -6.67 8.6e-11    -0.42    -0.23 1.04 0.11
## 
## Residual Standard Error =  0.95  with  389  degrees of freedom
## 
##  Multiple Regression
##          R   R2  Ruw R2uw Shrunken R2 SE of R2 overall F df1 df2        p
## PCGPA 0.33 0.11 0.22 0.05         0.1     0.03     15.39   3 389 1.75e-09
```

```
lm_HAMNat_via_Uni_TMS_3_corr
```

```
## Call: lmCor(y = PCGPA ~ Gender + Age + GPA + HAMNat_total, data = Cor_Mat_HAMNat_via_Uni_TMS_Incumbents_Corr, 
##     n.obs = nobs(lm_HAMNat_via_Uni_TMS_3), plot = F)
## 
## Multiple Regression from matrix input 
## 
##  DV =  PCGPA 
##              slope   se     t       p lower.ci upper.ci  VIF Vy.x
## Gender        0.00 0.05  0.08 9.4e-01    -0.09     0.10 1.05 0.00
## Age          -0.08 0.05 -1.57 1.2e-01    -0.19     0.02 1.21 0.00
## GPA           0.10 0.05  1.75 8.2e-02    -0.01     0.20 1.32 0.01
## HAMNat_total -0.30 0.05 -5.86 9.6e-09    -0.40    -0.20 1.15 0.10
## 
## Residual Standard Error =  0.95  with  388  degrees of freedom
## 
##  Multiple Regression
##          R   R2  Ruw R2uw Shrunken R2 SE of R2 overall F df1 df2        p
## PCGPA 0.34 0.11 0.28 0.08         0.1     0.03     12.37   4 388 1.77e-09
```

```
anova(lm_HAMNat_via_Uni_TMS_1_corr, lm_HAMNat_via_Uni_TMS_2a_corr, lm_HAMNat_via_Uni_TMS_3_corr)
```

```
## Model 1 = lmCor(y = PCGPA ~ Gender + Age, data = Cor_Mat_HAMNat_via_Uni_TMS_Incumbents_Corr, 
##     n.obs = nobs(lm_HAMNat_via_Uni_TMS_1), plot = F)
## Model 2 = lmCor(y = PCGPA ~ Gender + Age + GPA, data = Cor_Mat_HAMNat_via_Uni_TMS_Incumbents_Corr, 
##     n.obs = nobs(lm_HAMNat_via_Uni_TMS_2a), plot = F)
## Model 3 = lmCor(y = PCGPA ~ Gender + Age + GPA + HAMNat_total, data = Cor_Mat_HAMNat_via_Uni_TMS_Incumbents_Corr, 
##     n.obs = nobs(lm_HAMNat_via_Uni_TMS_3), plot = F)
```

```
## $PCGPA
##   Res Df   Res SS Diff df  Diff SS        F     Pr(F > )
## 1    390 390.5081      NA       NA       NA           NA
## 2    389 378.4883       1 12.01986 13.41433 2.842646e-04
## 3    388 347.6659       1 30.82233 34.39814 9.624090e-09
```

```
anova(lm_HAMNat_via_Uni_TMS_1_corr, lm_HAMNat_via_Uni_TMS_2b_corr, lm_HAMNat_via_Uni_TMS_3_corr)
```

```
## Model 1 = lmCor(y = PCGPA ~ Gender + Age, data = Cor_Mat_HAMNat_via_Uni_TMS_Incumbents_Corr, 
##     n.obs = nobs(lm_HAMNat_via_Uni_TMS_1), plot = F)
## Model 2 = lmCor(y = PCGPA ~ Gender + Age + HAMNat_total, data = Cor_Mat_HAMNat_via_Uni_TMS_Incumbents_Corr, 
##     n.obs = nobs(lm_HAMNat_via_Uni_TMS_2b), plot = F)
## Model 3 = lmCor(y = PCGPA ~ Gender + Age + GPA + HAMNat_total, data = Cor_Mat_HAMNat_via_Uni_TMS_Incumbents_Corr, 
##     n.obs = nobs(lm_HAMNat_via_Uni_TMS_3), plot = F)
```

```
## $PCGPA
##   Res Df   Res SS Diff df   Diff SS        F     Pr(F > )
## 1    390 390.5081      NA        NA       NA           NA
## 2    389 350.3985       1 40.109650 44.76293 7.767538e-11
## 3    388 347.6659       1  2.732538  3.04955 8.155091e-02
```

```
#Criterion: M1

#Correcting for range restriction
Cov_Mat_HAMNat_via_Uni_TMS_Incumbents_M1 <- cov((dplyr::select(data_HAMNat_via_Uni_TMS, Gender, Age, GPA, HAMNat_total, M1)), use = "complete.obs")
Cor_Mat_HAMNat_via_Uni_TMS_Incumbents_Corr_M1 <- lMvrrc(rcov = Cov_Mat_HAMNat_via_Uni_TMS_Incumbents_M1, vnp = Cov_Mat_HAMNat_via_Uni_TMS_Testtakers, as_cor = T)
rownames(Cor_Mat_HAMNat_via_Uni_TMS_Incumbents_Corr_M1) <- colnames(Cor_Mat_HAMNat_via_Uni_TMS_Incumbents_Corr_M1) <- c('Gender', 'Age', 'GPA', 'HAMNat_total', 'M1')

#Models
lm_HAMNat_via_Uni_TMS_1_corr_M1 <- lmCor(M1 ~ Gender + Age, data = Cor_Mat_HAMNat_via_Uni_TMS_Incumbents_Corr_M1, n.obs = nobs(lm_HAMNat_via_Uni_TMS_M1_1), plot = F)
lm_HAMNat_via_Uni_TMS_2a_corr_M1 <- lmCor(M1 ~ Gender + Age + GPA, data = Cor_Mat_HAMNat_via_Uni_TMS_Incumbents_Corr_M1, n.obs = nobs(lm_HAMNat_via_Uni_TMS_M1_2a), plot = F)
lm_HAMNat_via_Uni_TMS_2b_corr_M1 <- lmCor(M1 ~ Gender + Age + HAMNat_total, data = Cor_Mat_HAMNat_via_Uni_TMS_Incumbents_Corr_M1, n.obs = nobs(lm_HAMNat_via_Uni_TMS_M1_2b), plot = F)
lm_HAMNat_via_Uni_TMS_3_corr_M1 <- lmCor(M1 ~ Gender + Age + GPA + HAMNat_total, data = Cor_Mat_HAMNat_via_Uni_TMS_Incumbents_Corr_M1, n.obs = nobs(lm_HAMNat_via_Uni_TMS_M1_3), plot = F)

#Results
lm_HAMNat_via_Uni_TMS_1_corr_M1
```

```
## Call: lmCor(y = M1 ~ Gender + Age, data = Cor_Mat_HAMNat_via_Uni_TMS_Incumbents_Corr_M1, 
##     n.obs = nobs(lm_HAMNat_via_Uni_TMS_M1_1), plot = F)
## 
## Multiple Regression from matrix input 
## 
##  DV =  M1 
##        slope  se    t    p lower.ci upper.ci VIF Vy.x
## Gender -0.12 0.1 -1.3 0.20    -0.31     0.07   1 0.02
## Age     0.10 0.1  1.0 0.32    -0.09     0.28   1 0.01
## 
## Residual Standard Error =  1  with  107  degrees of freedom
## 
##  Multiple Regression
##       R   R2  Ruw R2uw Shrunken R2 SE of R2 overall F df1 df2     p
## M1 0.15 0.02 0.15 0.02        0.01     0.03      1.32   2 107 0.272
```

```
lm_HAMNat_via_Uni_TMS_2a_corr_M1
```

```
## Call: lmCor(y = M1 ~ Gender + Age + GPA, data = Cor_Mat_HAMNat_via_Uni_TMS_Incumbents_Corr_M1, 
##     n.obs = nobs(lm_HAMNat_via_Uni_TMS_M1_2a), plot = F)
## 
## Multiple Regression from matrix input 
## 
##  DV =  M1 
##        slope   se     t    p lower.ci upper.ci VIF Vy.x
## Gender -0.13 0.09 -1.34 0.18    -0.32     0.06 1.0 0.02
## Age     0.03 0.10  0.26 0.79    -0.18     0.23 1.2 0.00
## GPA     0.17 0.10  1.60 0.11    -0.04     0.37 1.2 0.03
## 
## Residual Standard Error =  0.99  with  106  degrees of freedom
## 
##  Multiple Regression
##       R   R2 Ruw R2uw Shrunken R2 SE of R2 overall F df1 df2     p
## M1 0.22 0.05 0.2 0.04        0.02     0.04      1.75   3 106 0.162
```

```
lm_HAMNat_via_Uni_TMS_2b_corr_M1
```

```
## Call: lmCor(y = M1 ~ Gender + Age + HAMNat_total, data = Cor_Mat_HAMNat_via_Uni_TMS_Incumbents_Corr_M1, 
##     n.obs = nobs(lm_HAMNat_via_Uni_TMS_M1_2b), plot = F)
## 
## Multiple Regression from matrix input 
## 
##  DV =  M1 
##              slope   se     t       p lower.ci upper.ci  VIF Vy.x
## Gender       -0.03 0.09 -0.35 7.2e-01    -0.20     0.14 1.04 0.00
## Age           0.07 0.09  0.82 4.1e-01    -0.10     0.24 1.00 0.01
## HAMNat_total -0.46 0.09 -5.33 5.5e-07    -0.64    -0.29 1.04 0.22
## 
## Residual Standard Error =  0.89  with  106  degrees of freedom
## 
##  Multiple Regression
##       R   R2  Ruw R2uw Shrunken R2 SE of R2 overall F df1 df2        p
## M1 0.48 0.23 0.37 0.14        0.21     0.07     10.58   3 106 3.84e-06
```

```
lm_HAMNat_via_Uni_TMS_3_corr_M1
```

```
## Call: lmCor(y = M1 ~ Gender + Age + GPA + HAMNat_total, data = Cor_Mat_HAMNat_via_Uni_TMS_Incumbents_Corr_M1, 
##     n.obs = nobs(lm_HAMNat_via_Uni_TMS_M1_3), plot = F)
## 
## Multiple Regression from matrix input 
## 
##  DV =  M1 
##              slope   se     t       p lower.ci upper.ci  VIF Vy.x
## Gender       -0.03 0.09 -0.37 7.1e-01    -0.21     0.14 1.05 0.00
## Age           0.06 0.09  0.66 5.1e-01    -0.12     0.25 1.21 0.01
## GPA           0.02 0.10  0.20 8.4e-01    -0.18     0.21 1.32 0.00
## HAMNat_total -0.46 0.09 -5.01 2.3e-06    -0.64    -0.28 1.15 0.22
## 
## Residual Standard Error =  0.89  with  105  degrees of freedom
## 
##  Multiple Regression
##       R   R2  Ruw R2uw Shrunken R2 SE of R2 overall F df1 df2        p
## M1 0.48 0.23 0.36 0.13         0.2     0.07      7.87   4 105 1.38e-05
```

```
anova(lm_HAMNat_via_Uni_TMS_1_corr_M1, lm_HAMNat_via_Uni_TMS_2a_corr_M1, lm_HAMNat_via_Uni_TMS_3_corr_M1)
```

```
## Model 1 = lmCor(y = M1 ~ Gender + Age, data = Cor_Mat_HAMNat_via_Uni_TMS_Incumbents_Corr_M1, 
##     n.obs = nobs(lm_HAMNat_via_Uni_TMS_M1_1), plot = F)
## Model 2 = lmCor(y = M1 ~ Gender + Age + GPA, data = Cor_Mat_HAMNat_via_Uni_TMS_Incumbents_Corr_M1, 
##     n.obs = nobs(lm_HAMNat_via_Uni_TMS_M1_2a), plot = F)
## Model 3 = lmCor(y = M1 ~ Gender + Age + GPA + HAMNat_total, data = Cor_Mat_HAMNat_via_Uni_TMS_Incumbents_Corr_M1, 
##     n.obs = nobs(lm_HAMNat_via_Uni_TMS_M1_3), plot = F)
```

```
## $M1
##   Res Df    Res SS Diff df   Diff SS         F     Pr(F > )
## 1    107 106.38307      NA        NA        NA           NA
## 2    106 103.86659       1  2.516483  3.151002 7.875063e-02
## 3    105  83.85612       1 20.010471 25.056007 2.253669e-06
```

```
anova(lm_HAMNat_via_Uni_TMS_1_corr_M1, lm_HAMNat_via_Uni_TMS_2b_corr_M1, lm_HAMNat_via_Uni_TMS_3_corr_M1)
```

```
## Model 1 = lmCor(y = M1 ~ Gender + Age, data = Cor_Mat_HAMNat_via_Uni_TMS_Incumbents_Corr_M1, 
##     n.obs = nobs(lm_HAMNat_via_Uni_TMS_M1_1), plot = F)
## Model 2 = lmCor(y = M1 ~ Gender + Age + HAMNat_total, data = Cor_Mat_HAMNat_via_Uni_TMS_Incumbents_Corr_M1, 
##     n.obs = nobs(lm_HAMNat_via_Uni_TMS_M1_2b), plot = F)
## Model 3 = lmCor(y = M1 ~ Gender + Age + GPA + HAMNat_total, data = Cor_Mat_HAMNat_via_Uni_TMS_Incumbents_Corr_M1, 
##     n.obs = nobs(lm_HAMNat_via_Uni_TMS_M1_3), plot = F)
```

```
## $M1
##   Res Df    Res SS Diff df     Diff SS           F     Pr(F > )
## 1    107 106.38307      NA          NA          NA           NA
## 2    106  83.88842       1 22.49464703 28.16655555 6.155523e-07
## 3    105  83.85612       1  0.03230735  0.04045348 8.409856e-01
```

### 3.3.3 Regression analyses in universities with selection via HAM-Nat (uncorrected)

#### 3.3.3.1 TMS

```
#Criterion: PCGPA

#Models
lm_TMS_via_Uni_HAMNat_1 <- lm(PCGPA ~ Gender + Age, data = data_TMS_via_Uni_HAMNat)
lm_TMS_via_Uni_HAMNat_2a <- lm(PCGPA ~ Gender + Age + GPA, data = data_TMS_via_Uni_HAMNat)
lm_TMS_via_Uni_HAMNat_2b <- lm(PCGPA ~ Gender + Age + TMS_total, data = data_TMS_via_Uni_HAMNat)
lm_TMS_via_Uni_HAMNat_3 <- lm(PCGPA ~ Gender + Age + GPA + TMS_total, data = data_TMS_via_Uni_HAMNat)

#Results
summary(lm_TMS_via_Uni_HAMNat_1)
```

```
## 
## Call:
## lm(formula = PCGPA ~ Gender + Age, data = data_TMS_via_Uni_HAMNat)
## 
## Residuals:
##      Min       1Q   Median       3Q      Max 
## -1.09056 -0.42816 -0.03642  0.41498  1.61224 
## 
## Coefficients:
##             Estimate Std. Error t value Pr(>|t|)  
## (Intercept)  0.84004    0.47109   1.783   0.0762 .
## Gender       0.05960    0.09339   0.638   0.5242  
## Age          0.05413    0.02173   2.491   0.0136 *
## ---
## Signif. codes:  0 '***' 0.001 '**' 0.01 '*' 0.05 '.' 0.1 ' ' 1
## 
## Residual standard error: 0.6011 on 184 degrees of freedom
##   (1 Beobachtung als fehlend gelöscht)
## Multiple R-squared:  0.03431,    Adjusted R-squared:  0.02381 
## F-statistic: 3.268 on 2 and 184 DF,  p-value: 0.04029
```

```
summary(lm_TMS_via_Uni_HAMNat_2a)
```

```
## 
## Call:
## lm(formula = PCGPA ~ Gender + Age + GPA, data = data_TMS_via_Uni_HAMNat)
## 
## Residuals:
##      Min       1Q   Median       3Q      Max 
## -1.09581 -0.42123 -0.02465  0.34629  1.68554 
## 
## Coefficients:
##             Estimate Std. Error t value Pr(>|t|)  
## (Intercept)  0.98375    0.46809   2.102   0.0370 *
## Gender       0.02063    0.09341   0.221   0.8255  
## Age          0.02200    0.02500   0.880   0.3801  
## GPA          0.36270    0.14530   2.496   0.0134 *
## ---
## Signif. codes:  0 '***' 0.001 '**' 0.01 '*' 0.05 '.' 0.1 ' ' 1
## 
## Residual standard error: 0.5927 on 183 degrees of freedom
##   (1 Beobachtung als fehlend gelöscht)
## Multiple R-squared:  0.06611,    Adjusted R-squared:  0.0508 
## F-statistic: 4.318 on 3 and 183 DF,  p-value: 0.005719
```

```
summary(lm_TMS_via_Uni_HAMNat_2b)
```

```
## 
## Call:
## lm(formula = PCGPA ~ Gender + Age + TMS_total, data = data_TMS_via_Uni_HAMNat)
## 
## Residuals:
##     Min      1Q  Median      3Q     Max 
## -1.2010 -0.4036 -0.0605  0.3799  1.6164 
## 
## Coefficients:
##              Estimate Std. Error t value Pr(>|t|)    
## (Intercept)  4.110315   0.733182   5.606 7.52e-08 ***
## Gender       0.037853   0.086718   0.437   0.6630    
## Age          0.039653   0.020326   1.951   0.0526 .  
## TMS_total   -0.028698   0.005166  -5.555 9.68e-08 ***
## ---
## Signif. codes:  0 '***' 0.001 '**' 0.01 '*' 0.05 '.' 0.1 ' ' 1
## 
## Residual standard error: 0.5575 on 183 degrees of freedom
##   (1 Beobachtung als fehlend gelöscht)
## Multiple R-squared:  0.1736, Adjusted R-squared:  0.1601 
## F-statistic: 12.82 on 3 and 183 DF,  p-value: 1.221e-07
```

```
summary(lm_TMS_via_Uni_HAMNat_3)
```

```
## 
## Call:
## lm(formula = PCGPA ~ Gender + Age + GPA + TMS_total, data = data_TMS_via_Uni_HAMNat)
## 
## Residuals:
##      Min       1Q   Median       3Q      Max 
## -1.28576 -0.36704 -0.06162  0.37344  1.67711 
## 
## Coefficients:
##              Estimate Std. Error t value Pr(>|t|)    
## (Intercept)  4.120534   0.725472   5.680 5.26e-08 ***
## Gender       0.006239   0.086981   0.072   0.9429    
## Age          0.013469   0.023321   0.578   0.5643    
## GPA          0.300977   0.135725   2.218   0.0278 *  
## TMS_total   -0.027741   0.005130  -5.407 1.99e-07 ***
## ---
## Signif. codes:  0 '***' 0.001 '**' 0.01 '*' 0.05 '.' 0.1 ' ' 1
## 
## Residual standard error: 0.5516 on 182 degrees of freedom
##   (1 Beobachtung als fehlend gelöscht)
## Multiple R-squared:  0.1954, Adjusted R-squared:  0.1777 
## F-statistic: 11.05 on 4 and 182 DF,  p-value: 4.814e-08
```

```
anova(lm_TMS_via_Uni_HAMNat_1, lm_TMS_via_Uni_HAMNat_2a, lm_TMS_via_Uni_HAMNat_3)
```

```
## Analysis of Variance Table
## 
## Model 1: PCGPA ~ Gender + Age
## Model 2: PCGPA ~ Gender + Age + GPA
## Model 3: PCGPA ~ Gender + Age + GPA + TMS_total
##   Res.Df    RSS Df Sum of Sq       F   Pr(>F)    
## 1    184 66.473                                  
## 2    183 64.284  1    2.1887  7.1923 0.007995 ** 
## 3    182 55.385  1    8.8984 29.2408 1.99e-07 ***
## ---
## Signif. codes:  0 '***' 0.001 '**' 0.01 '*' 0.05 '.' 0.1 ' ' 1
```

```
anova(lm_TMS_via_Uni_HAMNat_1, lm_TMS_via_Uni_HAMNat_2b, lm_TMS_via_Uni_HAMNat_3)
```

```
## Analysis of Variance Table
## 
## Model 1: PCGPA ~ Gender + Age
## Model 2: PCGPA ~ Gender + Age + TMS_total
## Model 3: PCGPA ~ Gender + Age + GPA + TMS_total
##   Res.Df    RSS Df Sum of Sq       F    Pr(>F)    
## 1    184 66.473                                   
## 2    183 56.882  1    9.5907 31.5156 7.285e-08 ***
## 3    182 55.385  1    1.4965  4.9176   0.02782 *  
## ---
## Signif. codes:  0 '***' 0.001 '**' 0.01 '*' 0.05 '.' 0.1 ' ' 1
```

```
#Criterion: M1

#Models
lm_TMS_via_Uni_HAMNat_M1_1 <- lm(M1 ~ Gender + Age, data = data_TMS_via_Uni_HAMNat)
lm_TMS_via_Uni_HAMNat_M1_2a <- lm(M1 ~ Gender + Age + GPA, data = data_TMS_via_Uni_HAMNat)
lm_TMS_via_Uni_HAMNat_M1_2b <- lm(M1 ~ Gender + Age + TMS_total, data = data_TMS_via_Uni_HAMNat)
lm_TMS_via_Uni_HAMNat_M1_3 <- lm(M1 ~ Gender + Age + GPA + TMS_total, data = data_TMS_via_Uni_HAMNat)

#Results
summary(lm_TMS_via_Uni_HAMNat_M1_1)
```

```
## 
## Call:
## lm(formula = M1 ~ Gender + Age, data = data_TMS_via_Uni_HAMNat)
## 
## Residuals:
##     Min      1Q  Median      3Q     Max 
## -1.2000 -0.6243 -0.1327  0.5353  1.9009 
## 
## Coefficients:
##             Estimate Std. Error t value Pr(>|t|)
## (Intercept)  1.63672    1.06093   1.543    0.127
## Gender      -0.17644    0.17835  -0.989    0.325
## Age          0.03362    0.05046   0.666    0.507
## 
## Residual standard error: 0.8079 on 87 degrees of freedom
##   (98 Beobachtungen als fehlend gelöscht)
## Multiple R-squared:  0.0172, Adjusted R-squared:  -0.005389 
## F-statistic: 0.7615 on 2 and 87 DF,  p-value: 0.4701
```

```
summary(lm_TMS_via_Uni_HAMNat_M1_2a)
```

```
## 
## Call:
## lm(formula = M1 ~ Gender + Age + GPA, data = data_TMS_via_Uni_HAMNat)
## 
## Residuals:
##     Min      1Q  Median      3Q     Max 
## -1.3935 -0.5479 -0.1230  0.5903  1.7032 
## 
## Coefficients:
##             Estimate Std. Error t value Pr(>|t|)   
## (Intercept)  2.28653    1.02461   2.232  0.02824 * 
## Gender      -0.23951    0.17011  -1.408  0.16273   
## Age         -0.06827    0.05694  -1.199  0.23389   
## GPA          0.96678    0.29331   3.296  0.00143 **
## ---
## Signif. codes:  0 '***' 0.001 '**' 0.01 '*' 0.05 '.' 0.1 ' ' 1
## 
## Residual standard error: 0.7657 on 86 degrees of freedom
##   (98 Beobachtungen als fehlend gelöscht)
## Multiple R-squared:  0.1274, Adjusted R-squared:  0.097 
## F-statistic: 4.187 on 3 and 86 DF,  p-value: 0.008136
```

```
summary(lm_TMS_via_Uni_HAMNat_M1_2b)
```

```
## 
## Call:
## lm(formula = M1 ~ Gender + Age + TMS_total, data = data_TMS_via_Uni_HAMNat)
## 
## Residuals:
##     Min      1Q  Median      3Q     Max 
## -1.2960 -0.5144 -0.1583  0.4463  2.2768 
## 
## Coefficients:
##             Estimate Std. Error t value Pr(>|t|)    
## (Intercept)  5.51125    1.53645   3.587 0.000555 ***
## Gender      -0.23833    0.16984  -1.403 0.164135    
## Age          0.03023    0.04778   0.633 0.528642    
## TMS_total   -0.03618    0.01086  -3.332 0.001273 ** 
## ---
## Signif. codes:  0 '***' 0.001 '**' 0.01 '*' 0.05 '.' 0.1 ' ' 1
## 
## Residual standard error: 0.7647 on 86 degrees of freedom
##   (98 Beobachtungen als fehlend gelöscht)
## Multiple R-squared:  0.1296, Adjusted R-squared:  0.09921 
## F-statistic: 4.267 on 3 and 86 DF,  p-value: 0.007375
```

```
summary(lm_TMS_via_Uni_HAMNat_M1_3)
```

```
## 
## Call:
## lm(formula = M1 ~ Gender + Age + GPA + TMS_total, data = data_TMS_via_Uni_HAMNat)
## 
## Residuals:
##      Min       1Q   Median       3Q      Max 
## -1.51955 -0.51295 -0.08223  0.51851  1.97485 
## 
## Coefficients:
##             Estimate Std. Error t value Pr(>|t|)    
## (Intercept)  5.70229    1.46705   3.887 0.000201 ***
## Gender      -0.28863    0.16284  -1.772 0.079898 .  
## Age         -0.06080    0.05431  -1.119 0.266119    
## GPA          0.86692    0.28129   3.082 0.002772 ** 
## TMS_total   -0.03252    0.01043  -3.119 0.002476 ** 
## ---
## Signif. codes:  0 '***' 0.001 '**' 0.01 '*' 0.05 '.' 0.1 ' ' 1
## 
## Residual standard error: 0.7295 on 85 degrees of freedom
##   (98 Beobachtungen als fehlend gelöscht)
## Multiple R-squared:  0.2171, Adjusted R-squared:  0.1802 
## F-statistic: 5.891 on 4 and 85 DF,  p-value: 0.0003113
```

```
anova(lm_TMS_via_Uni_HAMNat_M1_1, lm_TMS_via_Uni_HAMNat_M1_2a, lm_TMS_via_Uni_HAMNat_M1_3)
```

```
## Analysis of Variance Table
## 
## Model 1: M1 ~ Gender + Age
## Model 2: M1 ~ Gender + Age + GPA
## Model 3: M1 ~ Gender + Age + GPA + TMS_total
##   Res.Df    RSS Df Sum of Sq       F    Pr(>F)    
## 1     87 56.786                                   
## 2     86 50.417  1    6.3694 11.9675 0.0008487 ***
## 3     85 45.239  1    5.1784  9.7297 0.0024757 ** 
## ---
## Signif. codes:  0 '***' 0.001 '**' 0.01 '*' 0.05 '.' 0.1 ' ' 1
```

```
anova(lm_TMS_via_Uni_HAMNat_M1_1, lm_TMS_via_Uni_HAMNat_M1_2b, lm_TMS_via_Uni_HAMNat_M1_3)
```

```
## Analysis of Variance Table
## 
## Model 1: M1 ~ Gender + Age
## Model 2: M1 ~ Gender + Age + TMS_total
## Model 3: M1 ~ Gender + Age + GPA + TMS_total
##   Res.Df    RSS Df Sum of Sq       F    Pr(>F)    
## 1     87 56.786                                   
## 2     86 50.294  1    6.4925 12.1990 0.0007614 ***
## 3     85 45.239  1    5.0552  9.4982 0.0027721 ** 
## ---
## Signif. codes:  0 '***' 0.001 '**' 0.01 '*' 0.05 '.' 0.1 ' ' 1
```

#### 3.3.3.2 HAM-Nat

```
#Criterion: PCGPA

#Models
lm_HAMNat_via_Uni_HAMNat_1 <- lm(PCGPA ~ Gender + Age, data = data_HAMNat_via_Uni_HAMNat)
lm_HAMNat_via_Uni_HAMNat_2a <- lm(PCGPA ~ Gender + Age + GPA, data = data_HAMNat_via_Uni_HAMNat)
lm_HAMNat_via_Uni_HAMNat_2b <- lm(PCGPA ~ Gender + Age + HAMNat_total, data = data_HAMNat_via_Uni_HAMNat)
lm_HAMNat_via_Uni_HAMNat_3 <- lm(PCGPA ~ Gender + Age + GPA + HAMNat_total, data = data_HAMNat_via_Uni_HAMNat)

#Results
summary(lm_HAMNat_via_Uni_HAMNat_1)
```

```
## 
## Call:
## lm(formula = PCGPA ~ Gender + Age, data = data_HAMNat_via_Uni_HAMNat)
## 
## Residuals:
##      Min       1Q   Median       3Q      Max 
## -1.07981 -0.49224 -0.04001  0.41309  1.74343 
## 
## Coefficients:
##             Estimate Std. Error t value Pr(>|t|)    
## (Intercept)  1.14765    0.30742   3.733 0.000226 ***
## Gender       0.01657    0.07281   0.228 0.820170    
## Age          0.03981    0.01373   2.900 0.004000 ** 
## ---
## Signif. codes:  0 '***' 0.001 '**' 0.01 '*' 0.05 '.' 0.1 ' ' 1
## 
## Residual standard error: 0.6271 on 303 degrees of freedom
##   (2 Beobachtungen als fehlend gelöscht)
## Multiple R-squared:  0.02738,    Adjusted R-squared:  0.02096 
## F-statistic: 4.265 on 2 and 303 DF,  p-value: 0.0149
```

```
summary(lm_HAMNat_via_Uni_HAMNat_2a)
```

```
## 
## Call:
## lm(formula = PCGPA ~ Gender + Age + GPA, data = data_HAMNat_via_Uni_HAMNat)
## 
## Residuals:
##      Min       1Q   Median       3Q      Max 
## -1.10586 -0.49603 -0.02802  0.38929  1.74969 
## 
## Coefficients:
##              Estimate Std. Error t value Pr(>|t|)    
## (Intercept)  1.261594   0.308875   4.084 5.66e-05 ***
## Gender      -0.001021   0.072643  -0.014   0.9888    
## Age          0.017634   0.016527   1.067   0.2868    
## GPA          0.241237   0.101809   2.370   0.0184 *  
## ---
## Signif. codes:  0 '***' 0.001 '**' 0.01 '*' 0.05 '.' 0.1 ' ' 1
## 
## Residual standard error: 0.6223 on 302 degrees of freedom
##   (2 Beobachtungen als fehlend gelöscht)
## Multiple R-squared:  0.04514,    Adjusted R-squared:  0.03565 
## F-statistic: 4.758 on 3 and 302 DF,  p-value: 0.002945
```

```
summary(lm_HAMNat_via_Uni_HAMNat_2b)
```

```
## 
## Call:
## lm(formula = PCGPA ~ Gender + Age + HAMNat_total, data = data_HAMNat_via_Uni_HAMNat)
## 
## Residuals:
##      Min       1Q   Median       3Q      Max 
## -1.14716 -0.42383 -0.04039  0.37439  1.73792 
## 
## Coefficients:
##              Estimate Std. Error t value Pr(>|t|)    
## (Intercept)   1.53315    0.29774   5.149 4.72e-07 ***
## Gender        0.09420    0.07007   1.344  0.17983    
## Age           0.03522    0.01301   2.708  0.00716 ** 
## HAMNat_total -0.30056    0.04971  -6.046 4.38e-09 ***
## ---
## Signif. codes:  0 '***' 0.001 '**' 0.01 '*' 0.05 '.' 0.1 ' ' 1
## 
## Residual standard error: 0.5932 on 302 degrees of freedom
##   (2 Beobachtungen als fehlend gelöscht)
## Multiple R-squared:  0.1324, Adjusted R-squared:  0.1238 
## F-statistic: 15.36 on 3 and 302 DF,  p-value: 2.509e-09
```

```
summary(lm_HAMNat_via_Uni_HAMNat_3)
```

```
## 
## Call:
## lm(formula = PCGPA ~ Gender + Age + GPA + HAMNat_total, data = data_HAMNat_via_Uni_HAMNat)
## 
## Residuals:
##      Min       1Q   Median       3Q      Max 
## -1.23971 -0.42027 -0.03878  0.38264  1.78929 
## 
## Coefficients:
##              Estimate Std. Error t value Pr(>|t|)    
## (Intercept)   1.69065    0.29804   5.673 3.30e-08 ***
## Gender        0.07612    0.06935   1.098  0.27327    
## Age           0.00777    0.01561   0.498  0.61909    
## GPA           0.29639    0.09609   3.084  0.00223 ** 
## HAMNat_total -0.31421    0.04923  -6.383 6.55e-10 ***
## ---
## Signif. codes:  0 '***' 0.001 '**' 0.01 '*' 0.05 '.' 0.1 ' ' 1
## 
## Residual standard error: 0.585 on 301 degrees of freedom
##   (2 Beobachtungen als fehlend gelöscht)
## Multiple R-squared:  0.159,  Adjusted R-squared:  0.1478 
## F-statistic: 14.22 on 4 and 301 DF,  p-value: 1.203e-10
```

```
anova(lm_HAMNat_via_Uni_HAMNat_1, lm_HAMNat_via_Uni_HAMNat_2a, lm_HAMNat_via_Uni_HAMNat_3)
```

```
## Analysis of Variance Table
## 
## Model 1: PCGPA ~ Gender + Age
## Model 2: PCGPA ~ Gender + Age + GPA
## Model 3: PCGPA ~ Gender + Age + GPA + HAMNat_total
##   Res.Df    RSS Df Sum of Sq       F    Pr(>F)    
## 1    303 119.14                                   
## 2    302 116.97  1    2.1746  6.3534   0.01223 *  
## 3    301 103.02  1   13.9450 40.7434 6.554e-10 ***
## ---
## Signif. codes:  0 '***' 0.001 '**' 0.01 '*' 0.05 '.' 0.1 ' ' 1
```

```
anova(lm_HAMNat_via_Uni_HAMNat_1, lm_HAMNat_via_Uni_HAMNat_2b, lm_HAMNat_via_Uni_HAMNat_3)
```

```
## Analysis of Variance Table
## 
## Model 1: PCGPA ~ Gender + Age
## Model 2: PCGPA ~ Gender + Age + HAMNat_total
## Model 3: PCGPA ~ Gender + Age + GPA + HAMNat_total
##   Res.Df    RSS Df Sum of Sq       F    Pr(>F)    
## 1    303 119.14                                   
## 2    302 106.28  1    12.864 37.5836 2.744e-09 ***
## 3    301 103.02  1     3.256  9.5132  0.002229 ** 
## ---
## Signif. codes:  0 '***' 0.001 '**' 0.01 '*' 0.05 '.' 0.1 ' ' 1
```

```
#Criterion: M1

#Models
lm_HAMNat_via_Uni_HAMNat_M1_1 <- lm(M1 ~ Gender + Age, data = data_HAMNat_via_Uni_HAMNat)
lm_HAMNat_via_Uni_HAMNat_M1_2a <- lm(M1 ~ Gender + Age + GPA, data = data_HAMNat_via_Uni_HAMNat)
lm_HAMNat_via_Uni_HAMNat_M1_2b <- lm(M1 ~ Gender + Age + HAMNat_total, data = data_HAMNat_via_Uni_HAMNat)
lm_HAMNat_via_Uni_HAMNat_M1_3 <- lm(M1 ~ Gender + Age + GPA + HAMNat_total, data = data_HAMNat_via_Uni_HAMNat)

#Results
summary(lm_HAMNat_via_Uni_HAMNat_M1_1)
```

```
## 
## Call:
## lm(formula = M1 ~ Gender + Age, data = data_HAMNat_via_Uni_HAMNat)
## 
## Residuals:
##      Min       1Q   Median       3Q      Max 
## -1.48678 -0.62509 -0.00642  0.39454  2.18573 
## 
## Coefficients:
##             Estimate Std. Error t value Pr(>|t|)    
## (Intercept)  0.05938    0.55117   0.108  0.91439    
## Gender      -0.07050    0.13594  -0.519  0.60497    
## Age          0.09607    0.02430   3.954  0.00013 ***
## ---
## Signif. codes:  0 '***' 0.001 '**' 0.01 '*' 0.05 '.' 0.1 ' ' 1
## 
## Residual standard error: 0.7516 on 120 degrees of freedom
##   (185 Beobachtungen als fehlend gelöscht)
## Multiple R-squared:  0.1165, Adjusted R-squared:  0.1018 
## F-statistic: 7.912 on 2 and 120 DF,  p-value: 0.0005922
```

```
summary(lm_HAMNat_via_Uni_HAMNat_M1_2a)
```

```
## 
## Call:
## lm(formula = M1 ~ Gender + Age + GPA, data = data_HAMNat_via_Uni_HAMNat)
## 
## Residuals:
##      Min       1Q   Median       3Q      Max 
## -1.47193 -0.53803 -0.05577  0.44008  2.01823 
## 
## Coefficients:
##             Estimate Std. Error t value Pr(>|t|)   
## (Intercept)  0.31661    0.53930   0.587  0.55827   
## Gender      -0.08715    0.13151  -0.663  0.50880   
## Age          0.04365    0.02903   1.503  0.13538   
## GPA          0.55382    0.18028   3.072  0.00264 **
## ---
## Signif. codes:  0 '***' 0.001 '**' 0.01 '*' 0.05 '.' 0.1 ' ' 1
## 
## Residual standard error: 0.7265 on 119 degrees of freedom
##   (185 Beobachtungen als fehlend gelöscht)
## Multiple R-squared:  0.1814, Adjusted R-squared:  0.1608 
## F-statistic: 8.791 on 3 and 119 DF,  p-value: 2.597e-05
```

```
summary(lm_HAMNat_via_Uni_HAMNat_M1_2b)
```

```
## 
## Call:
## lm(formula = M1 ~ Gender + Age + HAMNat_total, data = data_HAMNat_via_Uni_HAMNat)
## 
## Residuals:
##      Min       1Q   Median       3Q      Max 
## -1.27308 -0.55401 -0.04016  0.36458  2.32167 
## 
## Coefficients:
##              Estimate Std. Error t value Pr(>|t|)    
## (Intercept)   0.32829    0.54390   0.604 0.547263    
## Gender        0.02028    0.13591   0.149 0.881650    
## Age           0.09422    0.02362   3.989 0.000115 ***
## HAMNat_total -0.26700    0.09395  -2.842 0.005277 ** 
## ---
## Signif. codes:  0 '***' 0.001 '**' 0.01 '*' 0.05 '.' 0.1 ' ' 1
## 
## Residual standard error: 0.7304 on 119 degrees of freedom
##   (185 Beobachtungen als fehlend gelöscht)
## Multiple R-squared:  0.1727, Adjusted R-squared:  0.1518 
## F-statistic: 8.278 on 3 and 119 DF,  p-value: 4.783e-05
```

```
summary(lm_HAMNat_via_Uni_HAMNat_M1_3)
```

```
## 
## Call:
## lm(formula = M1 ~ Gender + Age + GPA + HAMNat_total, data = data_HAMNat_via_Uni_HAMNat)
## 
## Residuals:
##      Min       1Q   Median       3Q      Max 
## -1.33258 -0.51398 -0.03666  0.40665  2.13699 
## 
## Coefficients:
##                Estimate Std. Error t value Pr(>|t|)   
## (Intercept)   0.5586364  0.5319959   1.050  0.29583   
## Gender       -0.0005735  0.1317270  -0.004  0.99653   
## Age           0.0443696  0.0282510   1.571  0.11897   
## GPA           0.5277289  0.1756817   3.004  0.00326 **
## HAMNat_total -0.2523350  0.0910616  -2.771  0.00649 **
## ---
## Signif. codes:  0 '***' 0.001 '**' 0.01 '*' 0.05 '.' 0.1 ' ' 1
## 
## Residual standard error: 0.707 on 118 degrees of freedom
##   (185 Beobachtungen als fehlend gelöscht)
## Multiple R-squared:  0.2314, Adjusted R-squared:  0.2054 
## F-statistic: 8.883 on 4 and 118 DF,  p-value: 2.639e-06
```

```
anova(lm_HAMNat_via_Uni_HAMNat_M1_1, lm_HAMNat_via_Uni_HAMNat_M1_2a, lm_HAMNat_via_Uni_HAMNat_M1_3)
```

```
## Analysis of Variance Table
## 
## Model 1: M1 ~ Gender + Age
## Model 2: M1 ~ Gender + Age + GPA
## Model 3: M1 ~ Gender + Age + GPA + HAMNat_total
##   Res.Df    RSS Df Sum of Sq      F   Pr(>F)   
## 1    120 67.793                                
## 2    119 62.812  1    4.9810 9.9663 0.002024 **
## 3    118 58.974  1    3.8376 7.6786 0.006494 **
## ---
## Signif. codes:  0 '***' 0.001 '**' 0.01 '*' 0.05 '.' 0.1 ' ' 1
```

```
anova(lm_HAMNat_via_Uni_HAMNat_M1_1, lm_HAMNat_via_Uni_HAMNat_M1_2b, lm_HAMNat_via_Uni_HAMNat_M1_3)
```

```
## Analysis of Variance Table
## 
## Model 1: M1 ~ Gender + Age
## Model 2: M1 ~ Gender + Age + HAMNat_total
## Model 3: M1 ~ Gender + Age + GPA + HAMNat_total
##   Res.Df    RSS Df Sum of Sq      F   Pr(>F)   
## 1    120 67.793                                
## 2    119 63.484  1    4.3089 8.6216 0.003995 **
## 3    118 58.974  1    4.5097 9.0234 0.003255 **
## ---
## Signif. codes:  0 '***' 0.001 '**' 0.01 '*' 0.05 '.' 0.1 ' ' 1
```

### 3.3.4 Regression analyses in universities with selection via HAM-Nat (corrected)

#### 3.3.4.1 TMS

```
#Criterion: PCGPA

#Correcting for range restriction
data_TMS_via_Uni_HAMNat$Gender <- as.numeric(data_TMS_via_Uni_HAMNat$Gender)
Cov_Mat_TMS_via_Uni_HAMNat_Incumbents <- cov((dplyr::select(data_TMS_via_Uni_HAMNat, Gender, Age, GPA, TMS_total, PCGPA)), use = "complete.obs")
Cov_Mat_TMS_via_Uni_HAMNat_Testtakers <- cov((dplyr::select(data_TMS_Testtakers, Gender, Age, GPA, TMS_total)))
Cor_Mat_TMS_via_Uni_HAMNat_Incumbents_Corr <- lMvrrc(rcov = Cov_Mat_TMS_via_Uni_HAMNat_Incumbents, vnp = Cov_Mat_TMS_via_Uni_HAMNat_Testtakers, as_cor = T)
rownames(Cor_Mat_TMS_via_Uni_HAMNat_Incumbents_Corr) <- colnames(Cor_Mat_TMS_via_Uni_HAMNat_Incumbents_Corr) <- c('Gender', 'Age', 'GPA', 'TMS_total', 'PCGPA')

#Models
lm_TMS_via_Uni_HAMNat_1_corr <- lmCor(PCGPA ~ Gender + Age, data = Cor_Mat_TMS_via_Uni_HAMNat_Incumbents_Corr, n.obs = nobs(lm_TMS_via_Uni_HAMNat_1), plot = F)
lm_TMS_via_Uni_HAMNat_2a_corr <- lmCor(PCGPA ~ Gender + Age + GPA, data = Cor_Mat_TMS_via_Uni_HAMNat_Incumbents_Corr, n.obs = nobs(lm_TMS_via_Uni_HAMNat_2a), plot = F)
lm_TMS_via_Uni_HAMNat_2b_corr <- lmCor(PCGPA ~ Gender + Age + TMS_total, data = Cor_Mat_TMS_via_Uni_HAMNat_Incumbents_Corr, n.obs = nobs(lm_TMS_via_Uni_HAMNat_2b), plot = F)
lm_TMS_via_Uni_HAMNat_3_corr <- lmCor(PCGPA ~ Gender + Age + GPA + TMS_total, data = Cor_Mat_TMS_via_Uni_HAMNat_Incumbents_Corr, n.obs = nobs(lm_TMS_via_Uni_HAMNat_3), plot = F)

#Results
lm_TMS_via_Uni_HAMNat_1_corr
```

```
## Call: lmCor(y = PCGPA ~ Gender + Age, data = Cor_Mat_TMS_via_Uni_HAMNat_Incumbents_Corr, 
##     n.obs = nobs(lm_TMS_via_Uni_HAMNat_1), plot = F)
## 
## Multiple Regression from matrix input 
## 
##  DV =  PCGPA 
##        slope   se     t      p lower.ci upper.ci VIF Vy.x
## Gender -0.03 0.07 -0.45 0.6500    -0.17     0.11   1 0.00
## Age     0.22 0.07  3.08 0.0024     0.08     0.36   1 0.05
## 
## Residual Standard Error =  0.98  with  184  degrees of freedom
## 
##  Multiple Regression
##          R   R2  Ruw R2uw Shrunken R2 SE of R2 overall F df1 df2       p
## PCGPA 0.22 0.05 0.18 0.03        0.04     0.03       4.8   2 184 0.00932
```

```
lm_TMS_via_Uni_HAMNat_2a_corr
```

```
## Call: lmCor(y = PCGPA ~ Gender + Age + GPA, data = Cor_Mat_TMS_via_Uni_HAMNat_Incumbents_Corr, 
##     n.obs = nobs(lm_TMS_via_Uni_HAMNat_2a), plot = F)
## 
## Multiple Regression from matrix input 
## 
##  DV =  PCGPA 
##        slope   se     t       p lower.ci upper.ci  VIF Vy.x
## Gender -0.04 0.07 -0.51 6.1e-01    -0.17     0.10 1.00 0.00
## Age     0.07 0.08  0.96 3.4e-01    -0.08     0.23 1.27 0.02
## GPA     0.32 0.08  4.12 5.8e-05     0.17     0.47 1.27 0.11
## 
## Residual Standard Error =  0.94  with  183  degrees of freedom
## 
##  Multiple Regression
##          R   R2  Ruw R2uw Shrunken R2 SE of R2 overall F df1 df2        p
## PCGPA 0.36 0.13 0.31 0.09        0.12     0.04      9.13   3 183 1.17e-05
```

```
lm_TMS_via_Uni_HAMNat_2b_corr
```

```
## Call: lmCor(y = PCGPA ~ Gender + Age + TMS_total, data = Cor_Mat_TMS_via_Uni_HAMNat_Incumbents_Corr, 
##     n.obs = nobs(lm_TMS_via_Uni_HAMNat_2b), plot = F)
## 
## Multiple Regression from matrix input 
## 
##  DV =  PCGPA 
##           slope   se     t       p lower.ci upper.ci  VIF Vy.x
## Gender     0.01 0.06  0.16 8.7e-01    -0.12     0.14 1.01 0.00
## Age        0.14 0.07  2.17 3.1e-02     0.01     0.27 1.04 0.03
## TMS_total -0.45 0.07 -6.89 8.9e-11    -0.58    -0.32 1.04 0.21
## 
## Residual Standard Error =  0.88  with  183  degrees of freedom
## 
##  Multiple Regression
##         R   R2  Ruw R2uw Shrunken R2 SE of R2 overall F df1 df2        p
## PCGPA 0.5 0.25 0.39 0.15        0.23     0.05     19.81   3 183 3.63e-11
```

```
lm_TMS_via_Uni_HAMNat_3_corr
```

```
## Call: lmCor(y = PCGPA ~ Gender + Age + GPA + TMS_total, data = Cor_Mat_TMS_via_Uni_HAMNat_Incumbents_Corr, 
##     n.obs = nobs(lm_TMS_via_Uni_HAMNat_3), plot = F)
## 
## Multiple Regression from matrix input 
## 
##  DV =  PCGPA 
##           slope   se     t       p lower.ci upper.ci  VIF Vy.x
## Gender     0.00 0.06  0.07 9.5e-01    -0.12     0.13 1.01 0.00
## Age        0.05 0.07  0.70 4.9e-01    -0.09     0.19 1.28 0.01
## GPA        0.22 0.07  3.00 3.0e-03     0.07     0.36 1.34 0.08
## TMS_total -0.41 0.07 -6.17 4.2e-09    -0.54    -0.28 1.10 0.19
## 
## Residual Standard Error =  0.86  with  182  degrees of freedom
## 
##  Multiple Regression
##          R   R2  Ruw R2uw Shrunken R2 SE of R2 overall F df1 df2        p
## PCGPA 0.53 0.28 0.44  0.2        0.27     0.05     17.77   4 182 2.49e-12
```

```
anova(lm_TMS_via_Uni_HAMNat_1_corr, lm_TMS_via_Uni_HAMNat_2a_corr, lm_TMS_via_Uni_HAMNat_3_corr)
```

```
## Model 1 = lmCor(y = PCGPA ~ Gender + Age, data = Cor_Mat_TMS_via_Uni_HAMNat_Incumbents_Corr, 
##     n.obs = nobs(lm_TMS_via_Uni_HAMNat_1), plot = F)
## Model 2 = lmCor(y = PCGPA ~ Gender + Age + GPA, data = Cor_Mat_TMS_via_Uni_HAMNat_Incumbents_Corr, 
##     n.obs = nobs(lm_TMS_via_Uni_HAMNat_2a), plot = F)
## Model 3 = lmCor(y = PCGPA ~ Gender + Age + GPA + TMS_total, data = Cor_Mat_TMS_via_Uni_HAMNat_Incumbents_Corr, 
##     n.obs = nobs(lm_TMS_via_Uni_HAMNat_3), plot = F)
```

```
## $PCGPA
##   Res Df   Res SS Diff df  Diff SS        F     Pr(F > )
## 1    184 176.7841      NA       NA       NA           NA
## 2    183 161.7926       1 14.99147 20.39696 1.125325e-05
## 3    182 133.7674       1 28.02527 38.13036 4.207382e-09
```

```
anova(lm_TMS_via_Uni_HAMNat_1_corr, lm_TMS_via_Uni_HAMNat_2b_corr, lm_TMS_via_Uni_HAMNat_3_corr)
```

```
## Model 1 = lmCor(y = PCGPA ~ Gender + Age, data = Cor_Mat_TMS_via_Uni_HAMNat_Incumbents_Corr, 
##     n.obs = nobs(lm_TMS_via_Uni_HAMNat_1), plot = F)
## Model 2 = lmCor(y = PCGPA ~ Gender + Age + TMS_total, data = Cor_Mat_TMS_via_Uni_HAMNat_Incumbents_Corr, 
##     n.obs = nobs(lm_TMS_via_Uni_HAMNat_2b), plot = F)
## Model 3 = lmCor(y = PCGPA ~ Gender + Age + GPA + TMS_total, data = Cor_Mat_TMS_via_Uni_HAMNat_Incumbents_Corr, 
##     n.obs = nobs(lm_TMS_via_Uni_HAMNat_3), plot = F)
```

```
## $PCGPA
##   Res Df   Res SS Diff df   Diff SS         F     Pr(F > )
## 1    184 176.7841      NA        NA        NA           NA
## 2    183 140.3972       1 36.386956 49.507032 3.829059e-11
## 3    182 133.7674       1  6.629783  9.020289 3.045807e-03
```

```
#Criterion: M1

#Correcting for range restriction
Cov_Mat_TMS_via_Uni_HAMNat_Incumbents_M1 <- cov((dplyr::select(data_TMS_via_Uni_HAMNat, Gender, Age, GPA, TMS_total, M1)), use = "complete.obs")
Cor_Mat_TMS_via_Uni_HAMNat_Incumbents_Corr_M1 <- lMvrrc(rcov = Cov_Mat_TMS_via_Uni_HAMNat_Incumbents_M1, vnp = Cov_Mat_TMS_via_Uni_HAMNat_Testtakers, as_cor = T)
rownames(Cor_Mat_TMS_via_Uni_HAMNat_Incumbents_Corr_M1) <- colnames(Cor_Mat_TMS_via_Uni_HAMNat_Incumbents_Corr_M1) <- c('Gender', 'Age', 'GPA', 'TMS_total', 'M1')

#Models
lm_TMS_via_Uni_HAMNat_M1_1_corr <- lmCor(M1 ~ Gender + Age, data = Cor_Mat_TMS_via_Uni_HAMNat_Incumbents_Corr_M1, n.obs = nobs(lm_TMS_via_Uni_HAMNat_M1_1), plot = F)
lm_TMS_via_Uni_HAMNat_M1_2a_corr <- lmCor(M1 ~ Gender + Age + GPA, data = Cor_Mat_TMS_via_Uni_HAMNat_Incumbents_Corr_M1, n.obs = nobs(lm_TMS_via_Uni_HAMNat_M1_2a), plot = F)
lm_TMS_via_Uni_HAMNat_M1_2b_corr <- lmCor(M1 ~ Gender + Age + TMS_total, data = Cor_Mat_TMS_via_Uni_HAMNat_Incumbents_Corr_M1, n.obs = nobs(lm_TMS_via_Uni_HAMNat_M1_2b), plot = F)
lm_TMS_via_Uni_HAMNat_M1_3_corr <- lmCor(M1 ~ Gender + Age + GPA + TMS_total, data = Cor_Mat_TMS_via_Uni_HAMNat_Incumbents_Corr_M1, n.obs = nobs(lm_TMS_via_Uni_HAMNat_M1_3), plot = F)

#Results
lm_TMS_via_Uni_HAMNat_M1_1_corr
```

```
## Call: lmCor(y = M1 ~ Gender + Age, data = Cor_Mat_TMS_via_Uni_HAMNat_Incumbents_Corr_M1, 
##     n.obs = nobs(lm_TMS_via_Uni_HAMNat_M1_1), plot = F)
## 
## Multiple Regression from matrix input 
## 
##  DV =  M1 
##        slope   se     t    p lower.ci upper.ci VIF Vy.x
## Gender -0.17 0.11 -1.62 0.11    -0.38     0.04   1 0.03
## Age     0.11 0.11  1.02 0.31    -0.10     0.32   1 0.01
## 
## Residual Standard Error =  0.99  with  87  degrees of freedom
## 
##  Multiple Regression
##      R   R2  Ruw R2uw Shrunken R2 SE of R2 overall F df1 df2     p
## M1 0.2 0.04 0.19 0.04        0.02     0.04      1.74   2  87 0.181
```

```
lm_TMS_via_Uni_HAMNat_M1_2a_corr
```

```
## Call: lmCor(y = M1 ~ Gender + Age + GPA, data = Cor_Mat_TMS_via_Uni_HAMNat_Incumbents_Corr_M1, 
##     n.obs = nobs(lm_TMS_via_Uni_HAMNat_M1_2a), plot = F)
## 
## Multiple Regression from matrix input 
## 
##  DV =  M1 
##        slope   se     t       p lower.ci upper.ci  VIF  Vy.x
## Gender -0.18 0.09 -1.89 6.2e-02    -0.36     0.01 1.00  0.03
## Age    -0.14 0.10 -1.33 1.9e-01    -0.35     0.07 1.27 -0.01
## GPA     0.54 0.10  5.14 1.7e-06     0.33     0.74 1.27  0.25
## 
## Residual Standard Error =  0.87  with  86  degrees of freedom
## 
##  Multiple Regression
##       R   R2  Ruw R2uw Shrunken R2 SE of R2 overall F df1 df2        p
## M1 0.51 0.26 0.38 0.14        0.24     0.07     10.31   3  86 7.25e-06
```

```
lm_TMS_via_Uni_HAMNat_M1_2b_corr
```

```
## Call: lmCor(y = M1 ~ Gender + Age + TMS_total, data = Cor_Mat_TMS_via_Uni_HAMNat_Incumbents_Corr_M1, 
##     n.obs = nobs(lm_TMS_via_Uni_HAMNat_M1_2b), plot = F)
## 
## Multiple Regression from matrix input 
## 
##  DV =  M1 
##           slope  se     t       p lower.ci upper.ci  VIF Vy.x
## Gender    -0.13 0.1 -1.34 1.8e-01    -0.32     0.06 1.01 0.02
## Age        0.03 0.1  0.32 7.5e-01    -0.16     0.22 1.04 0.00
## TMS_total -0.43 0.1 -4.46 2.5e-05    -0.63    -0.24 1.04 0.19
## 
## Residual Standard Error =  0.9  with  86  degrees of freedom
## 
##  Multiple Regression
##       R   R2  Ruw R2uw Shrunken R2 SE of R2 overall F df1 df2        p
## M1 0.47 0.22 0.39 0.15        0.19     0.07      8.04   3  86 8.77e-05
```

```
lm_TMS_via_Uni_HAMNat_M1_3_corr
```

```
## Call: lmCor(y = M1 ~ Gender + Age + GPA + TMS_total, data = Cor_Mat_TMS_via_Uni_HAMNat_Incumbents_Corr_M1, 
##     n.obs = nobs(lm_TMS_via_Uni_HAMNat_M1_3), plot = F)
## 
## Multiple Regression from matrix input 
## 
##  DV =  M1 
##           slope   se     t       p lower.ci upper.ci  VIF  Vy.x
## Gender    -0.14 0.09 -1.64 1.1e-01    -0.31     0.03 1.01  0.02
## Age       -0.16 0.10 -1.65 1.0e-01    -0.35     0.03 1.28 -0.02
## GPA        0.45 0.10  4.52 1.9e-05     0.25     0.65 1.34  0.21
## TMS_total -0.34 0.09 -3.79 2.8e-04    -0.52    -0.16 1.10  0.15
## 
## Residual Standard Error =  0.81  with  85  degrees of freedom
## 
##  Multiple Regression
##       R   R2  Ruw R2uw Shrunken R2 SE of R2 overall F df1 df2        p
## M1 0.61 0.37 0.49 0.24        0.34     0.08     12.51   4  85 4.76e-08
```

```
anova(lm_TMS_via_Uni_HAMNat_M1_1_corr, lm_TMS_via_Uni_HAMNat_M1_2a_corr, lm_TMS_via_Uni_HAMNat_M1_3_corr)
```

```
## Model 1 = lmCor(y = M1 ~ Gender + Age, data = Cor_Mat_TMS_via_Uni_HAMNat_Incumbents_Corr_M1, 
##     n.obs = nobs(lm_TMS_via_Uni_HAMNat_M1_1), plot = F)
## Model 2 = lmCor(y = M1 ~ Gender + Age + GPA, data = Cor_Mat_TMS_via_Uni_HAMNat_Incumbents_Corr_M1, 
##     n.obs = nobs(lm_TMS_via_Uni_HAMNat_M1_2a), plot = F)
## Model 3 = lmCor(y = M1 ~ Gender + Age + GPA + TMS_total, data = Cor_Mat_TMS_via_Uni_HAMNat_Incumbents_Corr_M1, 
##     n.obs = nobs(lm_TMS_via_Uni_HAMNat_M1_3), plot = F)
```

```
## $M1
##   Res Df   Res SS Diff df   Diff SS        F     Pr(F > )
## 1     87 85.56791      NA        NA       NA           NA
## 2     86 65.46021       1 20.107694 30.51332 3.472916e-07
## 3     85 56.01337       1  9.446838 14.33553 2.843547e-04
```

```
anova(lm_TMS_via_Uni_HAMNat_M1_1_corr, lm_TMS_via_Uni_HAMNat_M1_2b_corr, lm_TMS_via_Uni_HAMNat_M1_3_corr)
```

```
## Model 1 = lmCor(y = M1 ~ Gender + Age, data = Cor_Mat_TMS_via_Uni_HAMNat_Incumbents_Corr_M1, 
##     n.obs = nobs(lm_TMS_via_Uni_HAMNat_M1_1), plot = F)
## Model 2 = lmCor(y = M1 ~ Gender + Age + TMS_total, data = Cor_Mat_TMS_via_Uni_HAMNat_Incumbents_Corr_M1, 
##     n.obs = nobs(lm_TMS_via_Uni_HAMNat_M1_2b), plot = F)
## Model 3 = lmCor(y = M1 ~ Gender + Age + GPA + TMS_total, data = Cor_Mat_TMS_via_Uni_HAMNat_Incumbents_Corr_M1, 
##     n.obs = nobs(lm_TMS_via_Uni_HAMNat_M1_3), plot = F)
```

```
## $M1
##   Res Df   Res SS Diff df  Diff SS        F     Pr(F > )
## 1     87 85.56791      NA       NA       NA           NA
## 2     86 69.50589       1 16.06201 24.37402 3.851124e-06
## 3     85 56.01337       1 13.49252 20.47483 1.948338e-05
```

#### 3.3.4.2 HAM-Nat

```
#Criterion: PCGPA

#Correcting for range restriction
Cov_Mat_HAMNat_via_Uni_HAMNat_Incumbents <- cov((dplyr::select(data_HAMNat_via_Uni_HAMNat, Gender, Age, GPA, HAMNat_total, PCGPA)), use = "complete.obs")
Cov_Mat_HAMNat_via_Uni_HAMNat_Testtakers <- cov((dplyr::select(data_HAMNat_Testtakers, Gender, Age, GPA, HAMNat_total)))
Cor_Mat_HAMNat_via_Uni_HAMNat_Incumbents_Corr <- lMvrrc(rcov = Cov_Mat_HAMNat_via_Uni_HAMNat_Incumbents, vnp = Cov_Mat_HAMNat_via_Uni_HAMNat_Testtakers, as_cor = T)
rownames(Cor_Mat_HAMNat_via_Uni_HAMNat_Incumbents_Corr) <- colnames(Cor_Mat_HAMNat_via_Uni_HAMNat_Incumbents_Corr) <- c('Gender', 'Age', 'GPA', 'HAMNat_total', 'PCGPA')

#Models
lm_HAMNat_via_Uni_HAMNat_1_corr <- lmCor(PCGPA ~ Gender + Age, data = Cor_Mat_HAMNat_via_Uni_HAMNat_Incumbents_Corr, n.obs = nobs(lm_HAMNat_via_Uni_HAMNat_1), plot = F)
lm_HAMNat_via_Uni_HAMNat_2a_corr <- lmCor(PCGPA ~ Gender + Age + GPA, data = Cor_Mat_HAMNat_via_Uni_HAMNat_Incumbents_Corr, n.obs = nobs(lm_HAMNat_via_Uni_HAMNat_2a), plot = F)
lm_HAMNat_via_Uni_HAMNat_2b_corr <- lmCor(PCGPA ~ Gender + Age + HAMNat_total, data = Cor_Mat_HAMNat_via_Uni_HAMNat_Incumbents_Corr, n.obs = nobs(lm_HAMNat_via_Uni_HAMNat_2b), plot = F)
lm_HAMNat_via_Uni_HAMNat_3_corr <- lmCor(PCGPA ~ Gender + Age + GPA + HAMNat_total, data = Cor_Mat_HAMNat_via_Uni_HAMNat_Incumbents_Corr, n.obs = nobs(lm_HAMNat_via_Uni_HAMNat_3), plot = F)

#Results
lm_HAMNat_via_Uni_HAMNat_1_corr
```

```
## Call: lmCor(y = PCGPA ~ Gender + Age, data = Cor_Mat_HAMNat_via_Uni_HAMNat_Incumbents_Corr, 
##     n.obs = nobs(lm_HAMNat_via_Uni_HAMNat_1), plot = F)
## 
## Multiple Regression from matrix input 
## 
##  DV =  PCGPA 
##        slope   se     t     p lower.ci upper.ci VIF Vy.x
## Gender -0.03 0.06 -0.45 0.650    -0.14     0.09   1 0.00
## Age     0.14 0.06  2.41 0.016     0.03     0.25   1 0.02
## 
## Residual Standard Error =  0.99  with  303  degrees of freedom
## 
##  Multiple Regression
##          R   R2  Ruw R2uw Shrunken R2 SE of R2 overall F df1 df2      p
## PCGPA 0.14 0.02 0.11 0.01        0.01     0.02      2.99   2 303 0.0516
```

```
lm_HAMNat_via_Uni_HAMNat_2a_corr
```

```
## Call: lmCor(y = PCGPA ~ Gender + Age + GPA, data = Cor_Mat_HAMNat_via_Uni_HAMNat_Incumbents_Corr, 
##     n.obs = nobs(lm_HAMNat_via_Uni_HAMNat_2a), plot = F)
## 
## Multiple Regression from matrix input 
## 
##  DV =  PCGPA 
##        slope   se     t       p lower.ci upper.ci VIF Vy.x
## Gender -0.03 0.05 -0.58 5.6e-01    -0.14     0.07 1.0 0.00
## Age     0.00 0.06 -0.01 9.9e-01    -0.12     0.12 1.2 0.00
## GPA     0.34 0.06  5.70 2.8e-08     0.22     0.46 1.2 0.11
## 
## Residual Standard Error =  0.95  with  302  degrees of freedom
## 
##  Multiple Regression
##          R   R2  Ruw R2uw Shrunken R2 SE of R2 overall F df1 df2        p
## PCGPA 0.34 0.11 0.26 0.07        0.11     0.03     13.05   3 302 4.88e-08
```

```
lm_HAMNat_via_Uni_HAMNat_2b_corr
```

```
## Call: lmCor(y = PCGPA ~ Gender + Age + HAMNat_total, data = Cor_Mat_HAMNat_via_Uni_HAMNat_Incumbents_Corr, 
##     n.obs = nobs(lm_HAMNat_via_Uni_HAMNat_2b), plot = F)
## 
## Multiple Regression from matrix input 
## 
##  DV =  PCGPA 
##              slope   se     t       p lower.ci upper.ci  VIF Vy.x
## Gender        0.07 0.05  1.30 1.9e-01    -0.03     0.17 1.04 0.00
## Age           0.11 0.05  2.21 2.8e-02     0.01     0.21 1.00 0.02
## HAMNat_total -0.46 0.05 -8.92 4.5e-17    -0.56    -0.36 1.04 0.21
## 
## Residual Standard Error =  0.89  with  302  degrees of freedom
## 
##  Multiple Regression
##          R   R2  Ruw R2uw Shrunken R2 SE of R2 overall F df1 df2        p
## PCGPA 0.47 0.22 0.33 0.11        0.22     0.04     29.05   3 302 1.57e-16
```

```
lm_HAMNat_via_Uni_HAMNat_3_corr
```

```
## Call: lmCor(y = PCGPA ~ Gender + Age + GPA + HAMNat_total, data = Cor_Mat_HAMNat_via_Uni_HAMNat_Incumbents_Corr, 
##     n.obs = nobs(lm_HAMNat_via_Uni_HAMNat_3), plot = F)
## 
## Multiple Regression from matrix input 
## 
##  DV =  PCGPA 
##              slope   se     t       p lower.ci upper.ci  VIF Vy.x
## Gender        0.05 0.05  1.02 3.1e-01    -0.05     0.15 1.05 0.00
## Age           0.03 0.05  0.55 5.8e-01    -0.08     0.14 1.21 0.00
## GPA           0.21 0.06  3.67 2.9e-04     0.10     0.32 1.32 0.07
## HAMNat_total -0.40 0.05 -7.60 3.9e-13    -0.51    -0.30 1.15 0.18
## 
## Residual Standard Error =  0.87  with  301  degrees of freedom
## 
##  Multiple Regression
##          R   R2 Ruw R2uw Shrunken R2 SE of R2 overall F df1 df2        p
## PCGPA 0.51 0.26 0.4 0.16        0.25     0.04     26.05   4 301 1.47e-18
```

```
anova(lm_HAMNat_via_Uni_HAMNat_1_corr, lm_HAMNat_via_Uni_HAMNat_2a_corr, lm_HAMNat_via_Uni_HAMNat_3_corr)
```

```
## Model 1 = lmCor(y = PCGPA ~ Gender + Age, data = Cor_Mat_HAMNat_via_Uni_HAMNat_Incumbents_Corr, 
##     n.obs = nobs(lm_HAMNat_via_Uni_HAMNat_1), plot = F)
## Model 2 = lmCor(y = PCGPA ~ Gender + Age + GPA, data = Cor_Mat_HAMNat_via_Uni_HAMNat_Incumbents_Corr, 
##     n.obs = nobs(lm_HAMNat_via_Uni_HAMNat_2a), plot = F)
## Model 3 = lmCor(y = PCGPA ~ Gender + Age + GPA + HAMNat_total, data = Cor_Mat_HAMNat_via_Uni_HAMNat_Incumbents_Corr, 
##     n.obs = nobs(lm_HAMNat_via_Uni_HAMNat_3), plot = F)
```

```
## $PCGPA
##   Res Df   Res SS Diff df  Diff SS        F     Pr(F > )
## 1    303 299.0896      NA       NA       NA           NA
## 2    302 269.9936       1 29.09599 38.65561 1.678817e-09
## 3    301 226.5620       1 43.43161 57.70128 3.880077e-13
```

```
anova(lm_HAMNat_via_Uni_HAMNat_1_corr, lm_HAMNat_via_Uni_HAMNat_2b_corr, lm_HAMNat_via_Uni_HAMNat_3_corr)
```

```
## Model 1 = lmCor(y = PCGPA ~ Gender + Age, data = Cor_Mat_HAMNat_via_Uni_HAMNat_Incumbents_Corr, 
##     n.obs = nobs(lm_HAMNat_via_Uni_HAMNat_1), plot = F)
## Model 2 = lmCor(y = PCGPA ~ Gender + Age + HAMNat_total, data = Cor_Mat_HAMNat_via_Uni_HAMNat_Incumbents_Corr, 
##     n.obs = nobs(lm_HAMNat_via_Uni_HAMNat_2b), plot = F)
## Model 3 = lmCor(y = PCGPA ~ Gender + Age + GPA + HAMNat_total, data = Cor_Mat_HAMNat_via_Uni_HAMNat_Incumbents_Corr, 
##     n.obs = nobs(lm_HAMNat_via_Uni_HAMNat_3), plot = F)
```

```
## $PCGPA
##   Res Df   Res SS Diff df  Diff SS        F     Pr(F > )
## 1    303 299.0896      NA       NA       NA           NA
## 2    302 236.6911       1 62.39847 82.89979 1.211895e-17
## 3    301 226.5620       1 10.12913 13.45710 2.885667e-04
```

```
#Criterion: M1

#Correcting for range restriction
Cov_Mat_HAMNat_via_Uni_HAMNat_Incumbents_M1 <- cov((dplyr::select(data_HAMNat_via_Uni_HAMNat, Gender, Age, GPA, HAMNat_total, M1)), use = "complete.obs")
Cor_Mat_HAMNat_via_Uni_HAMNat_Incumbents_Corr_M1 <- lMvrrc(rcov = Cov_Mat_HAMNat_via_Uni_HAMNat_Incumbents_M1, vnp = Cov_Mat_HAMNat_via_Uni_HAMNat_Testtakers, as_cor = T)
rownames(Cor_Mat_HAMNat_via_Uni_HAMNat_Incumbents_Corr_M1) <- colnames(Cor_Mat_HAMNat_via_Uni_HAMNat_Incumbents_Corr_M1) <- c('Gender', 'Age', 'GPA', 'HAMNat_total', 'M1')

#Models
lm_HAMNat_via_Uni_HAMNat_1_corr_M1 <- lmCor(M1 ~ Gender + Age, data = Cor_Mat_HAMNat_via_Uni_HAMNat_Incumbents_Corr_M1, n.obs = nobs(lm_HAMNat_via_Uni_HAMNat_M1_1), plot = F)
lm_HAMNat_via_Uni_HAMNat_2a_corr_M1 <- lmCor(M1 ~ Gender + Age + GPA, data = Cor_Mat_HAMNat_via_Uni_HAMNat_Incumbents_Corr_M1, n.obs = nobs(lm_HAMNat_via_Uni_HAMNat_M1_2a), plot = F)
lm_HAMNat_via_Uni_HAMNat_2b_corr_M1 <- lmCor(M1 ~ Gender + Age + HAMNat_total, data = Cor_Mat_HAMNat_via_Uni_HAMNat_Incumbents_Corr_M1, n.obs = nobs(lm_HAMNat_via_Uni_HAMNat_M1_2b), plot = F)
lm_HAMNat_via_Uni_HAMNat_3_corr_M1 <- lmCor(M1 ~ Gender + Age + GPA + HAMNat_total, data = Cor_Mat_HAMNat_via_Uni_HAMNat_Incumbents_Corr_M1, n.obs = nobs(lm_HAMNat_via_Uni_HAMNat_M1_3), plot = F)

#Results
lm_HAMNat_via_Uni_HAMNat_1_corr_M1
```

```
## Call: lmCor(y = M1 ~ Gender + Age, data = Cor_Mat_HAMNat_via_Uni_HAMNat_Incumbents_Corr_M1, 
##     n.obs = nobs(lm_HAMNat_via_Uni_HAMNat_M1_1), plot = F)
## 
## Multiple Regression from matrix input 
## 
##  DV =  M1 
##        slope   se     t      p lower.ci upper.ci VIF Vy.x
## Gender -0.05 0.09 -0.56 0.5800    -0.22     0.12   1 0.00
## Age     0.28 0.09  3.23 0.0016     0.11     0.46   1 0.08
## 
## Residual Standard Error =  0.97  with  120  degrees of freedom
## 
##  Multiple Regression
##       R   R2  Ruw R2uw Shrunken R2 SE of R2 overall F df1 df2     p
## M1 0.29 0.08 0.23 0.05        0.07     0.05      5.34   2 120 0.006
```

```
lm_HAMNat_via_Uni_HAMNat_2a_corr_M1
```

```
## Call: lmCor(y = M1 ~ Gender + Age + GPA, data = Cor_Mat_HAMNat_via_Uni_HAMNat_Incumbents_Corr_M1, 
##     n.obs = nobs(lm_HAMNat_via_Uni_HAMNat_M1_2a), plot = F)
## 
## Multiple Regression from matrix input 
## 
##  DV =  M1 
##        slope   se     t       p lower.ci upper.ci VIF Vy.x
## Gender -0.06 0.08 -0.69 4.9e-01    -0.22     0.11 1.0 0.00
## Age     0.12 0.09  1.37 1.7e-01    -0.05     0.30 1.2 0.03
## GPA     0.39 0.09  4.41 2.3e-05     0.22     0.57 1.2 0.17
## 
## Residual Standard Error =  0.9  with  119  degrees of freedom
## 
##  Multiple Regression
##       R   R2 Ruw R2uw Shrunken R2 SE of R2 overall F df1 df2        p
## M1 0.46 0.21 0.4 0.16        0.19     0.06      10.6   3 119 3.15e-06
```

```
lm_HAMNat_via_Uni_HAMNat_2b_corr_M1
```

```
## Call: lmCor(y = M1 ~ Gender + Age + HAMNat_total, data = Cor_Mat_HAMNat_via_Uni_HAMNat_Incumbents_Corr_M1, 
##     n.obs = nobs(lm_HAMNat_via_Uni_HAMNat_M1_2b), plot = F)
## 
## Multiple Regression from matrix input 
## 
##  DV =  M1 
##              slope   se     t       p lower.ci upper.ci  VIF Vy.x
## Gender        0.02 0.08  0.27 7.9e-01    -0.14     0.19 1.04 0.00
## Age           0.26 0.08  3.21 1.7e-03     0.10     0.43 1.00 0.07
## HAMNat_total -0.35 0.08 -4.23 4.7e-05    -0.52    -0.19 1.04 0.13
## 
## Residual Standard Error =  0.9  with  119  degrees of freedom
## 
##  Multiple Regression
##       R  R2  Ruw R2uw Shrunken R2 SE of R2 overall F df1 df2        p
## M1 0.45 0.2 0.37 0.14        0.18     0.06     10.02   3 119 6.15e-06
```

```
lm_HAMNat_via_Uni_HAMNat_3_corr_M1
```

```
## Call: lmCor(y = M1 ~ Gender + Age + GPA + HAMNat_total, data = Cor_Mat_HAMNat_via_Uni_HAMNat_Incumbents_Corr_M1, 
##     n.obs = nobs(lm_HAMNat_via_Uni_HAMNat_M1_3), plot = F)
## 
## Multiple Regression from matrix input 
## 
##  DV =  M1 
##              slope   se     t       p lower.ci upper.ci  VIF Vy.x
## Gender        0.00 0.08  0.00 1.00000    -0.16     0.16 1.05 0.00
## Age           0.14 0.09  1.66 0.10000    -0.03     0.31 1.21 0.04
## GPA           0.31 0.09  3.42 0.00086     0.13     0.49 1.32 0.14
## HAMNat_total -0.27 0.08 -3.19 0.00180    -0.43    -0.10 1.15 0.10
## 
## Residual Standard Error =  0.87  with  118  degrees of freedom
## 
##  Multiple Regression
##       R   R2  Ruw R2uw Shrunken R2 SE of R2 overall F df1 df2        p
## M1 0.52 0.27 0.47 0.22        0.25     0.06     11.11   4 118 1.11e-07
```

```
anova(lm_HAMNat_via_Uni_HAMNat_1_corr_M1, lm_HAMNat_via_Uni_HAMNat_2a_corr_M1, lm_HAMNat_via_Uni_HAMNat_3_corr_M1)
```

```
## Model 1 = lmCor(y = M1 ~ Gender + Age, data = Cor_Mat_HAMNat_via_Uni_HAMNat_Incumbents_Corr_M1, 
##     n.obs = nobs(lm_HAMNat_via_Uni_HAMNat_M1_1), plot = F)
## Model 2 = lmCor(y = M1 ~ Gender + Age + GPA, data = Cor_Mat_HAMNat_via_Uni_HAMNat_Incumbents_Corr_M1, 
##     n.obs = nobs(lm_HAMNat_via_Uni_HAMNat_M1_2a), plot = F)
## Model 3 = lmCor(y = M1 ~ Gender + Age + GPA + HAMNat_total, data = Cor_Mat_HAMNat_via_Uni_HAMNat_Incumbents_Corr_M1, 
##     n.obs = nobs(lm_HAMNat_via_Uni_HAMNat_M1_3), plot = F)
```

```
## $M1
##   Res Df    Res SS Diff df   Diff SS        F     Pr(F > )
## 1    120 112.02953      NA        NA       NA           NA
## 2    119  96.27904       1 15.750496 20.97154 1.155345e-05
## 3    118  88.62290       1  7.656133 10.19402 1.806488e-03
```

```
anova(lm_HAMNat_via_Uni_HAMNat_1_corr_M1, lm_HAMNat_via_Uni_HAMNat_2b_corr_M1, lm_HAMNat_via_Uni_HAMNat_3_corr_M1)
```

```
## Model 1 = lmCor(y = M1 ~ Gender + Age, data = Cor_Mat_HAMNat_via_Uni_HAMNat_Incumbents_Corr_M1, 
##     n.obs = nobs(lm_HAMNat_via_Uni_HAMNat_M1_1), plot = F)
## Model 2 = lmCor(y = M1 ~ Gender + Age + HAMNat_total, data = Cor_Mat_HAMNat_via_Uni_HAMNat_Incumbents_Corr_M1, 
##     n.obs = nobs(lm_HAMNat_via_Uni_HAMNat_M1_2b), plot = F)
## Model 3 = lmCor(y = M1 ~ Gender + Age + GPA + HAMNat_total, data = Cor_Mat_HAMNat_via_Uni_HAMNat_Incumbents_Corr_M1, 
##     n.obs = nobs(lm_HAMNat_via_Uni_HAMNat_M1_3), plot = F)
```

```
## $M1
##   Res Df    Res SS Diff df   Diff SS        F     Pr(F > )
## 1    120 112.02953      NA        NA       NA           NA
## 2    119  97.40004       1 14.629490 19.47893 2.249934e-05
## 3    118  88.62290       1  8.777139 11.68662 8.647961e-04
```

# 4 Subtest analyses

## 4.1 TMS

```
#Criterion: PCGPA

#Correcting for range restriction
Cov_Mat_TMS_Testtakers_Subtests <- cov((dplyr::select(data_TMS_Testtakers, Gender, Age, GPA, BMS, QFP, DT, TC, MRT, FMT, VMT, VST)))
Cov_Mat_TMS_Incumbents_Subtests <- cov((dplyr::select(data_TMS_Incumbents, Gender, Age, GPA, BMS, QFP, DT, TC, MRT, FMT, VMT, VST, PCGPA)), use = "complete.obs")
Cor_Mat_TMS_Incumbents_Subtests <- cor((dplyr::select(data_TMS_Incumbents, Gender, Age, GPA, BMS, QFP, DT, TC, MRT, FMT, VMT, VST, PCGPA)), use = "complete.obs")
Cor_Mat_Corr_TMS_Incumbents_Subtests <- lMvrrc(rcov = Cov_Mat_TMS_Incumbents_Subtests, vnp = Cov_Mat_TMS_Testtakers_Subtests, as_cor = T)
rownames(Cor_Mat_Corr_TMS_Incumbents_Subtests) <- colnames(Cor_Mat_Corr_TMS_Incumbents_Subtests) <- c('Gender', 'Age', 'GPA', 'BMS', 'QFP', 'DT', 'TC', 'MRT', 'FMT', 'VMT', 'VST', 'PCGPA')

#Results
round(Cor_Mat_TMS_Incumbents_Subtests, 2)
```

```
##        Gender   Age   GPA   BMS   QFP    DT    TC   MRT   FMT   VMT   VST PCGPA
## Gender   1.00  0.06  0.10  0.11  0.21  0.16  0.14  0.08 -0.14 -0.12 -0.02 -0.09
## Age      0.06  1.00  0.59 -0.12 -0.19 -0.18 -0.08 -0.06 -0.07 -0.05 -0.07  0.12
## GPA      0.10  0.59  1.00 -0.09 -0.18 -0.17 -0.06  0.02 -0.04  0.00 -0.01  0.14
## BMS      0.11 -0.12 -0.09  1.00  0.47  0.52  0.59  0.26  0.21  0.25  0.16 -0.17
## QFP      0.21 -0.19 -0.18  0.47  1.00  0.57  0.41  0.27  0.17  0.18  0.20 -0.21
## DT       0.16 -0.18 -0.17  0.52  0.57  1.00  0.46  0.27  0.16  0.19  0.21 -0.14
## TC       0.14 -0.08 -0.06  0.59  0.41  0.46  1.00  0.25  0.20  0.23  0.14 -0.12
## MRT      0.08 -0.06  0.02  0.26  0.27  0.27  0.25  1.00  0.37  0.37  0.38 -0.09
## FMT     -0.14 -0.07 -0.04  0.21  0.17  0.16  0.20  0.37  1.00  0.40  0.31 -0.01
## VMT     -0.12 -0.05  0.00  0.25  0.18  0.19  0.23  0.37  0.40  1.00  0.31 -0.07
## VST     -0.02 -0.07 -0.01  0.16  0.20  0.21  0.14  0.38  0.31  0.31  1.00 -0.09
## PCGPA   -0.09  0.12  0.14 -0.17 -0.21 -0.14 -0.12 -0.09 -0.01 -0.07 -0.09  1.00
```

```
round(Cor_Mat_Corr_TMS_Incumbents_Subtests, 2)
```

```
##        Gender   Age   GPA   BMS   QFP    DT    TC   MRT   FMT   VMT   VST PCGPA
## Gender   1.00  0.05  0.03  0.12  0.23  0.18  0.12  0.08 -0.08 -0.09 -0.01 -0.10
## Age      0.05  1.00  0.46 -0.12 -0.18 -0.17 -0.09 -0.09 -0.10 -0.08 -0.09  0.11
## GPA      0.03  0.46  1.00 -0.25 -0.30 -0.28 -0.21 -0.12 -0.13 -0.14 -0.09  0.19
## BMS      0.12 -0.12 -0.25  1.00  0.54  0.59  0.64  0.36  0.27  0.33  0.26 -0.21
## QFP      0.23 -0.18 -0.30  0.54  1.00  0.62  0.49  0.36  0.24  0.28  0.26 -0.25
## DT       0.18 -0.17 -0.28  0.59  0.62  1.00  0.55  0.35  0.26  0.28  0.26 -0.18
## TC       0.12 -0.09 -0.21  0.64  0.49  0.55  1.00  0.36  0.28  0.33  0.25 -0.17
## MRT      0.08 -0.09 -0.12  0.36  0.36  0.35  0.36  1.00  0.43  0.43  0.44 -0.13
## FMT     -0.08 -0.10 -0.13  0.27  0.24  0.26  0.28  0.43  1.00  0.46  0.35 -0.05
## VMT     -0.09 -0.08 -0.14  0.33  0.28  0.28  0.33  0.43  0.46  1.00  0.35 -0.11
## VST     -0.01 -0.09 -0.09  0.26  0.26  0.26  0.25  0.44  0.35  0.35  1.00 -0.12
## PCGPA   -0.10  0.11  0.19 -0.21 -0.25 -0.18 -0.17 -0.13 -0.05 -0.11 -0.12  1.00
```

```
#Criterion: M1

#Correcting for range restriction
Cov_Mat_TMS_Incumbents_M1_Subtests <- cov((dplyr::select(data_TMS_Incumbents, Gender, Age, GPA, BMS, QFP, DT, TC, MRT, FMT, VMT, VST, M1)), use = "complete.obs")
Cor_Mat_TMS_Incumbents_M1_Subtests <- cor((dplyr::select(data_TMS_Incumbents, Gender, Age, GPA, BMS, QFP, DT, TC, MRT, FMT, VMT, VST, M1)), use = "complete.obs")
Cor_Mat_Corr_TMS_Incumbents_M1_Subtests <- lMvrrc(rcov = Cov_Mat_TMS_Incumbents_M1_Subtests, vnp = Cov_Mat_TMS_Testtakers_Subtests, as_cor = T)
rownames(Cor_Mat_Corr_TMS_Incumbents_M1_Subtests) <- colnames(Cor_Mat_Corr_TMS_Incumbents_M1_Subtests) <- c('Gender', 'Age', 'GPA', 'BMS', 'QFP', 'DT', 'TC', 'MRT', 'FMT', 'VMT', 'VST', 'M1')

#Results
round(Cor_Mat_TMS_Incumbents_M1_Subtests, 2)
```

```
##        Gender   Age   GPA   BMS   QFP    DT    TC   MRT   FMT   VMT   VST    M1
## Gender   1.00  0.04  0.11  0.04  0.22  0.11  0.07 -0.04 -0.23 -0.24  0.00 -0.12
## Age      0.04  1.00  0.64 -0.20 -0.19 -0.13 -0.14 -0.03  0.00  0.09  0.00  0.18
## GPA      0.11  0.64  1.00 -0.19 -0.15 -0.15 -0.10 -0.04 -0.08  0.02 -0.01  0.21
## BMS      0.04 -0.20 -0.19  1.00  0.47  0.56  0.59  0.24  0.15  0.25  0.23 -0.30
## QFP      0.22 -0.19 -0.15  0.47  1.00  0.57  0.36  0.28  0.12  0.19  0.20 -0.20
## DT       0.11 -0.13 -0.15  0.56  0.57  1.00  0.44  0.31  0.17  0.25  0.31 -0.24
## TC       0.07 -0.14 -0.10  0.59  0.36  0.44  1.00  0.14  0.13  0.20  0.19 -0.22
## MRT     -0.04 -0.03 -0.04  0.24  0.28  0.31  0.14  1.00  0.36  0.34  0.32 -0.08
## FMT     -0.23  0.00 -0.08  0.15  0.12  0.17  0.13  0.36  1.00  0.37  0.25  0.02
## VMT     -0.24  0.09  0.02  0.25  0.19  0.25  0.20  0.34  0.37  1.00  0.25  0.03
## VST      0.00  0.00 -0.01  0.23  0.20  0.31  0.19  0.32  0.25  0.25  1.00 -0.02
## M1      -0.12  0.18  0.21 -0.30 -0.20 -0.24 -0.22 -0.08  0.02  0.03 -0.02  1.00
```

```
round(Cor_Mat_Corr_TMS_Incumbents_M1_Subtests, 2)
```

```
##        Gender   Age   GPA   BMS   QFP    DT    TC   MRT   FMT   VMT   VST    M1
## Gender   1.00  0.05  0.03  0.12  0.23  0.18  0.12  0.08 -0.08 -0.09 -0.01 -0.15
## Age      0.05  1.00  0.46 -0.12 -0.18 -0.17 -0.09 -0.09 -0.10 -0.08 -0.09  0.12
## GPA      0.03  0.46  1.00 -0.25 -0.30 -0.28 -0.21 -0.12 -0.13 -0.14 -0.09  0.26
## BMS      0.12 -0.12 -0.25  1.00  0.54  0.59  0.64  0.36  0.27  0.33  0.26 -0.35
## QFP      0.23 -0.18 -0.30  0.54  1.00  0.62  0.49  0.36  0.24  0.28  0.26 -0.27
## DT       0.18 -0.17 -0.28  0.59  0.62  1.00  0.55  0.35  0.26  0.28  0.26 -0.30
## TC       0.12 -0.09 -0.21  0.64  0.49  0.55  1.00  0.36  0.28  0.33  0.25 -0.29
## MRT      0.08 -0.09 -0.12  0.36  0.36  0.35  0.36  1.00  0.43  0.43  0.44 -0.15
## FMT     -0.08 -0.10 -0.13  0.27  0.24  0.26  0.28  0.43  1.00  0.46  0.35 -0.05
## VMT     -0.09 -0.08 -0.14  0.33  0.28  0.28  0.33  0.43  0.46  1.00  0.35 -0.04
## VST     -0.01 -0.09 -0.09  0.26  0.26  0.26  0.25  0.44  0.35  0.35  1.00 -0.05
## M1      -0.15  0.12  0.26 -0.35 -0.27 -0.30 -0.29 -0.15 -0.05 -0.04 -0.05  1.00
```

## 4.2 HAM-Nat

```
#Criterion: PCGPA

#Correcting for range restriction
Cov_Mat_HAMNat_Testtakers_Subtests <- cov((dplyr::select(data_HAMNat_Testtakers, Gender, Age, GPA, KT, NRT, VRT)))
Cov_Mat_HAMNat_Incumbents_Subtests <- cov((dplyr::select(data_HAMNat_Incumbents, Gender, Age, GPA, KT, NRT, VRT, PCGPA)), use = "complete.obs")
Cor_Mat_HAMNat_Incumbents_Subtests <- cor((dplyr::select(data_HAMNat_Incumbents, Gender, Age, GPA, KT, NRT, VRT, PCGPA)), use = "complete.obs")
Cor_Mat_Corr_HAMNat_Incumbents_Subtests <- lMvrrc(rcov = Cov_Mat_HAMNat_Incumbents_Subtests, vnp = Cov_Mat_HAMNat_Testtakers_Subtests, as_cor = T)
rownames(Cor_Mat_Corr_HAMNat_Incumbents_Subtests) <- colnames(Cor_Mat_Corr_HAMNat_Incumbents_Subtests) <- c('Gender', 'Age', 'GPA', 'KT', 'NRT', 'VRT', 'PCGPA')

#Results
round(Cor_Mat_HAMNat_Incumbents_Subtests, 2)
```

```
##        Gender   Age   GPA    KT   NRT   VRT PCGPA
## Gender   1.00  0.04  0.09  0.19  0.23 -0.04 -0.03
## Age      0.04  1.00  0.55 -0.05 -0.07 -0.10  0.07
## GPA      0.09  0.55  1.00 -0.08 -0.07 -0.07  0.15
## KT       0.19 -0.05 -0.08  1.00  0.23  0.23 -0.34
## NRT      0.23 -0.07 -0.07  0.23  1.00  0.30 -0.15
## VRT     -0.04 -0.10 -0.07  0.23  0.30  1.00 -0.09
## PCGPA   -0.03  0.07  0.15 -0.34 -0.15 -0.09  1.00
```

```
round(Cor_Mat_Corr_HAMNat_Incumbents_Subtests, 2)
```

```
##        Gender   Age   GPA    KT   NRT   VRT PCGPA
## Gender   1.00  0.02  0.03  0.19  0.24  0.01 -0.03
## Age      0.02  1.00  0.41 -0.03 -0.10 -0.14  0.05
## GPA      0.03  0.41  1.00 -0.27 -0.18 -0.17  0.22
## KT       0.19 -0.03 -0.27  1.00  0.29  0.27 -0.35
## NRT      0.24 -0.10 -0.18  0.29  1.00  0.33 -0.17
## VRT      0.01 -0.14 -0.17  0.27  0.33  1.00 -0.11
## PCGPA   -0.03  0.05  0.22 -0.35 -0.17 -0.11  1.00
```

```
#Criterion: M1

#Correcting for range restriction
Cov_Mat_HAMNat_Incumbents_M1_Subtests <- cov((dplyr::select(data_HAMNat_Incumbents, Gender, Age, GPA, KT, NRT, VRT, M1)), use = "complete.obs")
Cor_Mat_HAMNat_Incumbents_M1_Subtests <- cor((dplyr::select(data_HAMNat_Incumbents, Gender, Age, GPA, KT, NRT, VRT, M1)), use = "complete.obs")
Cor_Mat_Corr_HAMNat_Incumbents_M1_Subtests <- lMvrrc(rcov = Cov_Mat_HAMNat_Incumbents_M1_Subtests, vnp = Cov_Mat_HAMNat_Testtakers_Subtests, as_cor = T)
rownames(Cor_Mat_Corr_HAMNat_Incumbents_M1_Subtests) <- colnames(Cor_Mat_Corr_HAMNat_Incumbents_M1_Subtests) <- c('Gender', 'Age', 'GPA', 'KT', 'NRT', 'VRT', 'M1')

#Results
round(Cor_Mat_HAMNat_Incumbents_M1_Subtests, 2)
```

```
##        Gender   Age   GPA    KT   NRT   VRT    M1
## Gender   1.00  0.09  0.11  0.21  0.15 -0.04 -0.10
## Age      0.09  1.00  0.58 -0.09 -0.06 -0.08  0.24
## GPA      0.11  0.58  1.00 -0.11 -0.05 -0.10  0.28
## KT       0.21 -0.09 -0.11  1.00  0.30  0.20 -0.46
## NRT      0.15 -0.06 -0.05  0.30  1.00  0.18 -0.23
## VRT     -0.04 -0.08 -0.10  0.20  0.18  1.00 -0.09
## M1      -0.10  0.24  0.28 -0.46 -0.23 -0.09  1.00
```

```
round(Cor_Mat_Corr_HAMNat_Incumbents_M1_Subtests, 2)
```

```
##        Gender   Age   GPA    KT   NRT   VRT    M1
## Gender   1.00  0.02  0.03  0.19  0.24  0.01 -0.11
## Age      0.02  1.00  0.41 -0.03 -0.10 -0.14  0.19
## GPA      0.03  0.41  1.00 -0.27 -0.18 -0.17  0.36
## KT       0.19 -0.03 -0.27  1.00  0.29  0.27 -0.47
## NRT      0.24 -0.10 -0.18  0.29  1.00  0.33 -0.25
## VRT      0.01 -0.14 -0.17  0.27  0.33  1.00 -0.15
## M1      -0.11  0.19  0.36 -0.47 -0.25 -0.15  1.00
```
