# Supplementary material for: Predictive validity of admission tests and educational attainment on preclinical academic performance – a multisite study
Source: BMC Med Educ. 2025 Sep 23;25:1255. doi: 10.1186/s12909-025-07974-2 (PMC12455761; doi:10.1186/s12909-025-07974-2)
Supplement: Supplementary file 3 — Supplementary Material 3. [file 12909_2025_7974_MOESM3_ESM.pdf]

Survey (English Translation)

[1] Did you enroll in a study course in human medicine, dentistry, veterinary medicine, or pharmacy at a German state university?

- Yes
- No

[2] In which study course did you enroll?

Answer this question only if the following conditions are met: The answer was "Yes" to question [1]

- Regular study course in human medicine
- Reform study course in human medicine
- Regular study course in dentistry
- Reform study course in dentistry
- Veterinary medicine
- Pharmacy

[3] At which German state university are you currently studying human medicine?

Answer this question only if the following conditions are met: The answer was equal to or less than "Reform study course in human medicine" to question [2] and the answer was "Yes" to question [1]

- Aachen, RWTH
- Augsburg, University of Augsburg
- Berlin, Charité
- Bielefeld, University of Bielefeld
- Bochum, Ruhr University
- Bonn, University of Bonn
- Dresden/Chemnitz, Technical University
- Duisburg-Essen, University
- Düsseldorf, Heinrich Heine University
- Erlangen-Nuremberg, Friedrich-Alexander-University
- Erlangen-Nuremberg/Bayreuth, University of Erlangen
- Frankfurt am Main, Johann Wolfgang Goethe University
- Freiburg, University of Freiburg
- Giessen, Justus Liebig University
- Göttingen, University of Göttingen
- Greifswald, University of Greifswald
- Halle-Wittenberg, University of Halle-Wittenberg
- Hamburg, UKE
- Hanover, Medical School
- Heidelberg, University of Heidelberg
- Heidelberg/Mannheim, University of Mannheim
- Jena, Friedrich Schiller University
- Kiel, University
- Cologne, University of Cologne
- Leipzig, University
- Lübeck, University of Lübeck
- Magdeburg, Otto von Guericke University
- Mainz, Johannes Gutenberg University
- Marburg, Philipps-Universität
- Munich, Ludwig Maximilians University
- Münster, University of Münster
- Oldenburg, Carl von Ossietzky University
- Regensburg, University of Regensburg
- Rostock, University of Rostock
- Saarbrücken, Saarland University
- Tübingen, Eberhardt Karls University
- Ulm, University of Ulm
- Würzburg, Julius-Maximilians-Universität

[4] At which German state university are you currently studying dentistry?

Answer this question only if the following conditions are met: The answer was "Regular study course in dentistry" or "Reform study course in dentistry" to question [2] and the answer was "Yes" to question [1]

- Aachen, RWTH
- Berlin, Charité
- Bonn, University of Bonn
- Dresden/Chemnitz, Technical University
- Düsseldorf, Heinrich Heine University
- Erlangen-Nuremberg, Friedrich-Alexander-University
- Frankfurt am Main, Johann Wolfgang Goethe University
- Freiburg, University of Freiburg
- Giessen, Justus Liebig University
- Göttingen, University of Göttingen
- Greifswald, University of Greifswald
- Halle-Wittenberg, Martin Luther University
- Hamburg, University/UKE
- Hanover, Medical School
- Heidelberg, Ruprecht-Karls-University
- Jena, Friedrich Schiller University
- Kiel, Christian-Albrechts-University
- Cologne, University of Cologne
- Leipzig, University
- Mainz, Johannes Gutenberg University
- Marburg, Philipps-Universität
- Munich, Ludwig-Maximilians-Universität
- Münster, University of Münster
- Regensburg, University of Regensburg
- Rostock, University of Rostock
- Saarbrücken, Saarland University
- Tübingen, Eberhardt Karls University
- Ulm, University of Ulm
- Würzburg, Julius-Maximilians-Universität

[5] At which German state university are you currently studying veterinary medicine?

Answer this question only if the following conditions are met: The answer was "Veterinary medicine" to question [2] and the answer was "Yes" to question [1]

- Berlin, Freie Universität
- Giessen, Justus Liebig University
- Hanover, University of Veterinary Medicine
- Leipzig, University
- Munich, Ludwig-Maximilians-Universität

[6] At which German state university are you currently studying pharmacy?

Answer this question only if the following conditions are met: The answer was "Pharmacy" to question [2] and the answer was "Yes" to question [1]

- Berlin, Freie Universität
- Bonn, University of Bonn
- Braunschweig, University of Technology
- Düsseldorf, Heinrich Heine University
- Erlangen-Nuremberg, Friedrich-Alexander-University
- Frankfurt am Main, Johann Wolfgang Goethe University
- Freiburg, University of Freiburg
- Greifswald, University of Greifswald
- Halle-Wittenberg, Martin Luther University
- Hamburg, University/UKE
- Heidelberg, Ruprecht-Karls-University
- Jena, Friedrich Schiller University
- Kiel, University
- Leipzig, University
- Mainz, Johannes Gutenberg University
- Marburg, Philipps-Universität
- Munich, Ludwig Maximilians University
- Münster, University of Münster
- Regensburg, University of Regensburg
- Saarbrücken, Saarland University
- Tübingen, Eberhardt Karls University
- Würzburg, Julius-Maximilians-Universität

[7] In which semester are you currently enrolled?

Answer this question only if the following conditions are met: Answer was "Yes" to question [1]

- In the 1st semester
- In the 2nd semester
- In the 3rd semester
- In the 4th semester
- In the 5th semester
- In the 6th semester
- In the 7th semester
- In the 8th semester
- In the 9th semester
- 10th semester or higher

[8] Are you currently within the regular period of study?

Answer this question only if the following conditions are met: Answer was "Yes" to question [1]

- Yes
- No, with a delay of *one* semester
- No, with a delay of *two* semesters
- No, with a delay of *three* semesters
- No, with a delay of *more than three* semesters

[9] What is your average grade for all exams taken (excluding the M1 or any substitute exams)? Please provide an estimate if you do not know your exact grade average.

Answer this question only if the following conditions are met: Answer was "Yes" to question [1]

- 1,0
- 1,1
- 1,2
- 1,3
- 1,4
- 1,5
- 1,6
- 1,7
- 1,8
- 1,9
- 2,0
- 2,1
- 2,2
- 2,3
- 2,4
- 2,5
- 2,6
- 2,7
- 2,8
- 2,9
- 3,0
- 3,1
- 3,2
- 3,3
- 3,4
- 3,5
- 3,6
- 3,7
- 3,8
- 3,9
- 4,0
- Provide no information
- I haven't received any grades yet

[10] Have you already completed the M1 or any substitute exam in your current course of study?

Answer this question only if the following conditions are met: Answer was equal to or greater than "In the 3rd semester" to question [7]

- Yes, the M1
- Yes, a substitute exam
- No

[11] Have you already taken the M1 or any substitute exam, but did not yet receive a grade?

Answer this question only if the following conditions are met: Answer was "No" to question [10]

- Yes
- No

[12] What grade did you get in the written M1?

Answer this question only if the following conditions are met: Answer was "Yes, the M1" to question [10]

- 1
- 2
- 3
- 4
- 5
- Provide no information

[13] What grade did you get in the oral M1?

Answer this question only if the following conditions are met: Answer was "Yes, the M1" to question [10]

- 1
- 2
- 3
- 4
- 5
- Provide no information

[14] What percentage of the maximum score did you get in the written M1 substitute exam? Please provide an estimate if you do not know your exact score.

Answer this question only if the following conditions are met: Answer was "Yes, a substitute exam" to question [10]

- 0% - 10%
- 11% - 20%
- 21% - 30%
- 31% - 40%
- 41% - 50%
- 51% - 60%
- 61% - 70%
- 71% - 80%
- 81% - 90%
- 91% - 100%
- Provide no information

[15] What percentage of the maximum score did you get in the oral M1 substitute exam? Please provide an estimate if you do not know your exact score.

Answer this question only if the following conditions are met: Answer was "Yes, a substitute exam" to question [10]

- 0% - 10%
- 11% - 20%
- 21% - 30%
- 31% - 40%
- 41% - 50%
- 51% - 60%
- 61% - 70%
- 71% - 80%
- 81% - 90%
- 91% - 100%
- Provide no information

[16] Have you already taken the M1 or any substitute exam and did not pass?

Answer this question only if the following conditions are met: Answer was "No" to question [10]

- Yes
- No

[17] Did you participate in the TMS?

- Yes
- No

[18] What year did you participate in the TMS?

Answer this question only if the following conditions are met: Answer was "Yes" to question [17]

- 2022
- 2021
- 2020
- 2019
- 2018
- 2017
- 2016
- 2015
- 2014
- 2013
- 2012
- 2011
- 2010
- Before 2010

[19] On the basis of which selection quota did you get accepted for your course of study?

Answer this question only if the following conditions are met: Answer was "Yes" to question [1]

- I don't know exactly.
- Abitur quota
- Specific selection procedure of the university
- Waiting time quota (up to admission year 2019)
- Aptitude quota (from admission year 2020)
- Hardship case
- Second study course
- Rural doctor quota
- Public Health Service Quota
- Other

[20] Where did you obtain your university entrance qualification (Abitur or similar exam)?

- Afghanistan
- Egypt
- Albania
- Algeria
- Andorra
- Angola
- Antigua and Barbuda
- Equatorial Guinea
- Argentina
- Armenia
- Azerbaijan
- Ethiopia
- Australia
- Bahamas
- Bahrain
- Bangladesh
- Barbados
- Belarus
- Belgium
- Belize
- Benin
- Bhutan
- Bolivia
- Bosnia and Herzegovina
- Botswana
- Brazil
- Brunei Darussalam
- Bulgaria
- Burkina Faso
- Burundi
- Cabo Verde
- Chile
- China
- Cook Islands
- Costa Rica
- Côte d'Ivoire
- Denmark
- Germany
- Dominica
- Dominican Republic
- Djibouti
- Ecuador
- El Salvador
- Eritrea
- Estonia
- Eswatini

- Fiji
- Finland
- France
- Gabon
- Gambia
- Georgia
- Ghana
- Grenada
- Greece
- Great Britain
- Guatemala
- Guinea
- Guinea-Bissau
- Guyana
- Haiti
- Honduras
- India
- Indonesia
- Iraq
- Iran
- Ireland
- Iceland
- Israel
- Italy
- Jamaica
- Japan
- Yemen
- Jordan
- Cambodia
- Cameroon
- Canada
- Kazakhstan
- Qatar
- Kenya
- Kyrgyzstan
- Kiribati
- Colombia
- Comoros
- Congo
- Korea, Democratic People's Republic of
- Korea, Republic of
- Kosovo
- Croatia
- Cuba
- Kuwait
- Laos
- Lesotho
- Latvia
- Lebanon
- Liberia
- Libya
- Liechtenstein
- Lithuania
- Luxembourg
- Madagascar
- Malawi
- Malaysia
- Maldives
- Mali
- Malta
- Morocco
- Marshall Islands
- Mauritania
- Mauritius
- Mexico
- Micronesia
- Moldova
- Monaco
- Mongolia
- Montenegro
- Mozambique
- Myanmar
- Namibia
- Nauru
- Nepal
- New Zealand
- Nicaragua
- Netherlands
- Niger
- Nigeria
- North Macedonia
- Norway
- Oman
- Austria
- Pakistan
- Palau
- Panama
- Papua New Guinea
- Paraguay
- Peru
- Philippines
- Poland
- Portugal
- Rwanda
- Romania
- Russian Federation
- Solomon Islands
- Zambia
- Samoa
- San Marino
- São Tomé and Príncipe
- Saudi Arabia
- Sweden
- Switzerland
- Senegal
- Serbia
- Seychelles
- Sierra Leone
- Zimbabwe
- Singapore
- Slovakia
- Slovenia
- Somalia
- Spain
- Sri Lanka
- Saint Kitts and Nevis
- Saint Lucia
- Saint Vincent and the Grenadines
- South Africa
- Sudan
- South Sudan
- Suriname
- Syria
- Tajikistan
- Taiwan
- Tanzania
- Thailand
- East Timor
- Togo
- Tonga
- Trinidad and Tobago
- Chad
- Czech Republic
- Tunisia
- Turkey
- Turkmenistan
- Tuvalu
- Uganda
- Ukraine
- Hungary
- Uruguay
- Uzbekistan
- Vanuatu
- Vatican City
- Venezuela
- U.A.E
- United States
- Viet Nam
- Central African Republic
- Cyprus

[21] In which German federal state did you acquire your university entrance qualification (Abitur or similar exam)?

Answer this question only if the following conditions are met: Answer was “Germany” to question [20]

- Baden-Württemberg
- Bavaria
- Berlin
- Brandenburg
- Bremen
- Hamburg
- Hesse
- Mecklenburg-Western Pomerania
- Lower Saxony
- North Rhine-Westphalia
- Rhineland-Palatinate
- Saarland
- Saxony
- Saxony-Anhalt
- Schleswig-Holstein
- Thuringia

[22] What final grade did you get (Abitur or similar exam)?

- 0,9
- 1,0
- 1,1
- 1,2
- 1,3
- 1,4
- 1,5
- 1,6
- 1,7
- 1,8
- 1,9
- 2,0
- 2,1
- 2,2
- 2,3
- 2,4
- 2,5
- 2,6
- 2,7
- 2,8
- 2,9
- 3,0
- 3,1
- 3,2
- 3,3
- 3,4
- 3,5
- 3,6
- 3,7
- 3,8
- 3,9
- 4,0

[23] Have you completed any vocational training?

- No
- Yes, in a non-medical field
- Yes, in a medical field
- Yes, in a dental field
- Yes, in a veterinary field
- Yes, in the pharmaceutical sector

[24] What is your gender?

- Female
- Male
- Gender-Divers

[25] What year were you born?

- 2006
- 2005
- 2004
- 2003
- 2002
- 2001
- 2000
- 1999
- 1998
- 1997
- 1996
- 1995
- 1994
- 1993
- 1992
- 1991
- 1990
- 1989
- 1988
- 1987
- 1986
- 1985
- 1984
- 1983
- 1982
- 1981
- 1980
- 1979
- 1978
- 1977
- 1976
- 1975
- 1974
- 1973
- 1972
- 1971
- 1970
- 1969
- 1968
- 1967
- 1966
- 1965
- Provide no information
